# CAGI, the Critical Assessment of Genome Interpretation, establishes progress and prospects for computational genetic variant interpretation methods

**DOI:** 10.1186/s13059-023-03113-6

**Published:** 2024-02-22

**Authors:** The Critical Assessment of Genome Interpretation Consortium, Shantanu Jain, Constantina Bakolitsa, Steven E. Brenner, Predrag Radivojac, John Moult, Susanna Repo, Roger A. Hoskins, Gaia Andreoletti, Daniel Barsky, Ajithavalli Chellapan, Hoyin Chu, Navya Dabbiru, Naveen K. Kollipara, Melissa Ly, Andrew J. Neumann, Lipika R. Pal, Eric Odell, Gaurav Pandey, Robin C. Peters-Petrulewicz, Rajgopal Srinivasan, Stephen F. Yee, Sri Jyothsna Yeleswarapu, Maya Zuhl, Ogun Adebali, Ayoti Patra, Michael A. Beer, Raghavendra Hosur, Jian Peng, Brady M. Bernard, Michael Berry, Shengcheng Dong, Alan P. Boyle, Aashish Adhikari, Jingqi Chen, Zhiqiang Hu, Robert Wang, Yaqiong Wang, Maximilian Miller, Yanran Wang, Yana Bromberg, Paola Turina, Emidio Capriotti, James J. Han, Kivilcim Ozturk, Hannah Carter, Giulia Babbi, Samuele Bovo, Pietro Di Lena, Pier Luigi Martelli, Castrense Savojardo, Rita Casadio, Melissa S. Cline, Greet De Baets, Sandra Bonache, Orland Díez, Sara Gutiérrez-Enríquez, Alejandro Fernández, Gemma Montalban, Lars Ootes, Selen Özkan, Natàlia Padilla, Casandra Riera, Xavier De la Cruz, Mark Diekhans, Peter J. Huwe, Qiong Wei, Qifang Xu, Roland L. Dunbrack, Valer Gotea, Laura Elnitski, Gennady Margolin, Piero Fariselli, Ivan V. Kulakovskiy, Vsevolod J. Makeev, Dmitry D. Penzar, Ilya E. Vorontsov, Alexander V. Favorov, Julia R. Forman, Marcia Hasenahuer, Maria S. Fornasari, Gustavo Parisi, Ziga Avsec, Muhammed H. Çelik, Thi Yen Duong Nguyen, Julien Gagneur, Fang-Yuan Shi, Matthew D. Edwards, Yuchun Guo, Kevin Tian, Haoyang Zeng, David K. Gifford, Jonathan Göke, Jan Zaucha, Julian Gough, Graham R. S. Ritchie, Adam Frankish, Jonathan M. Mudge, Jennifer Harrow, Erin L. Young, Yao Yu, Chad D. Huff, Katsuhiko Murakami, Yoko Nagai, Tadashi Imanishi, Christopher J. Mungall, Julius O. B. Jacobsen, Dongsup Kim, Chan-Seok Jeong, David T. Jones, Mulin Jun Li, Violeta Beleva Guthrie, Rohit Bhattacharya, Yun-Ching Chen, Christopher Douville, Jean Fan, Dewey Kim, David Masica, Noushin Niknafs, Sohini Sengupta, Collin Tokheim, Tychele N. Turner, Hui Ting Grace Yeo, Rachel Karchin, Sunyoung Shin, Rene Welch, Sunduz Keles, Yue Li, Manolis Kellis, Carles Corbi-Verge, Alexey V. Strokach, Philip M. Kim, Teri E. Klein, Rahul Mohan, Nicholas A. Sinnott-Armstrong, Michael Wainberg, Anshul Kundaje, Nina Gonzaludo, Angel C. Y. Mak, Aparna Chhibber, Hugo Y. K. Lam, Dvir Dahary, Simon Fishilevich, Doron Lancet, Insuk Lee, Benjamin Bachman, Panagiotis Katsonis, Rhonald C. Lua, Stephen J. Wilson, Olivier Lichtarge, Rajendra R. Bhat, Laksshman Sundaram, Vivek Viswanath, Riccardo Bellazzi, Giovanna Nicora, Ettore Rizzo, Ivan Limongelli, Aziz M. Mezlini, Ray Chang, Serra Kim, Carmen Lai, Robert O’Connor, Scott Topper, Jeroen van den Akker, Alicia Y. Zhou, Anjali D. Zimmer, Gilad Mishne, Timothy R. Bergquist, Marcus R. Breese, Rafael F. Guerrero, Yuxiang Jiang, Nikki Kiga, Biao Li, Matthew Mort, Kymberleigh A. Pagel, Vikas Pejaver, Moses H. Stamboulian, Janita Thusberg, Sean D. Mooney, Nuttinee Teerakulkittipong, Chen Cao, Kunal Kundu, Yizhou Yin, Chen-Hsin Yu, Michael Kleyman, Chiao-Feng Lin, Mary Stackpole, Stephen M. Mount, Gökcen Eraslan, Nikola S. Mueller, Tatsuhiko Naito, Aliz R. Rao, Johnathan R. Azaria, Aharon Brodie, Yanay Ofran, Aditi Garg, Debnath Pal, Alex Hawkins-Hooker, Henry Kenlay, John Reid, Eliseos J. Mucaki, Peter K. Rogan, Jana M. Schwarz, David B. Searls, Gyu Rie Lee, Chaok Seok, Andreas Krämer, Sohela Shah, ChengLai V. Huang, Jack F. Kirsch, Maxim Shatsky, Yue Cao, Haoran Chen, Mostafa Karimi, Oluwaseyi Moronfoye, Yuanfei Sun, Yang Shen, Ron Shigeta, Colby T. Ford, Conor Nodzak, Aneeta Uppal, Xinghua Shi, Thomas Joseph, Sujatha Kotte, Sadhna Rana, Aditya Rao, V. G. Saipradeep, Naveen Sivadasan, Uma Sunderam, Mario Stanke, Andrew Su, Ivan Adzhubey, Daniel M. Jordan, Shamil Sunyaev, Frederic Rousseau, Joost Schymkowitz, Joost Van Durme, Sean V. Tavtigian, Marco Carraro, Manuel Giollo, Silvio C. E. Tosatto, Orit Adato, Liran Carmel, Noa E. Cohen, Tzila Fenesh, Tamar Holtzer, Tamar Juven-Gershon, Ron Unger, Abhishek Niroula, Ayodeji Olatubosun, Jouni Väliaho, Yang Yang, Mauno Vihinen, Mary E. Wahl, Billy Chang, Ka Chun Chong, Inchi Hu, Rui Sun, William Ka Kei Wu, Xiaoxuan Xia, Benny C. Zee, Maggie H. Wang, Meng Wang, Chunlei Wu, Yutong Lu, Ken Chen, Yuedong Yang, Christopher M. Yates, Anat Kreimer, Zhongxia Yan, Nir Yosef, Huying Zhao, Zhipeng Wei, Zhaomin Yao, Fengfeng Zhou, Lukas Folkman, Yaoqi Zhou, Roxana Daneshjou, Russ B. Altman, Fumitaka Inoue, Nadav Ahituv, Adam P. Arkin, Federica Lovisa, Paolo Bonvini, Sarah Bowdin, Stefano Gianni, Elide Mantuano, Velia Minicozzi, Leonore Novak, Alessandra Pasquo, Annalisa Pastore, Maria Petrosino, Rita Puglisi, Angelo Toto, Liana Veneziano, Roberta Chiaraluce, Mad P. Ball, Jason R. Bobe, George M. Church, Valerio Consalvi, David N. Cooper, Bethany A. Buckley, Molly B. Sheridan, Garry R. Cutting, Maria Chiara Scaini, Kamil J. Cygan, Alger M. Fredericks, David T. Glidden, Christopher Neil, Christy L. Rhine, William G. Fairbrother, Aileen Y. Alontaga, Aron W. Fenton, Kenneth A. Matreyek, Lea M. Starita, Douglas M. Fowler, Britt-Sabina Löscher, Andre Franke, Scott I. Adamson, Brenton R. Graveley, Joe W. Gray, Mary J. Malloy, John P. Kane, Maria Kousi, Nicholas Katsanis, Max Schubach, Martin Kircher, Angel C. Y. Mak, Paul L. F. Tang, Pui-Yan Kwok, Richard H. Lathrop, Wyatt T. Clark, Guoying K. Yu, Jonathan H. LeBowitz, Francesco Benedicenti, Elisa Bettella, Stefania Bigoni, Federica Cesca, Isabella Mammi, Cristina Marino-Buslje, Donatella Milani, Angela Peron, Roberta Polli, Stefano Sartori, Franco Stanzial, Irene Toldo, Licia Turolla, Maria C. Aspromonte, Mariagrazia Bellini, Emanuela Leonardi, Xiaoming Liu, Christian Marshall, W. Richard McCombie, Lisa Elefanti, Chiara Menin, M. Stephen Meyn, Alessandra Murgia, Kari C. Y. Nadeau, Susan L. Neuhausen, Robert L. Nussbaum, Mehdi Pirooznia, James B. Potash, Dago F. Dimster-Denk, Jasper D. Rine, Jeremy R. Sanford, Michael Snyder, Atina G. Cote, Song Sun, Marta W. Verby, Jochen Weile, Frederick P. Roth, Ryan Tewhey, Pardis C. Sabeti, Joan Campagna, Marwan M. Refaat, Julianne Wojciak, Soren Grubb, Nicole Schmitt, Jay Shendure, Amanda B. Spurdle, Dimitri J. Stavropoulos, Nephi A. Walton, Peter P. Zandi, Elad Ziv, Wylie Burke, Flavia Chen, Lawrence R. Carr, Selena Martinez, Jodi Paik, Julie Harris-Wai, Mark Yarborough, Stephanie M. Fullerton, Barbara A. Koenig, Gregory McInnes, Dustin Shigaki, John-Marc Chandonia, Mabel Furutsuki, Laura Kasak, Changhua Yu, Rui Chen, Iddo Friedberg, Gad A. Getz, Qian Cong, Lisa N. Kinch, Jing Zhang, Nick V. Grishin, Alin Voskanian, Maricel G. Kann, Elizabeth Tran, Nilah M. Ioannidis, Jesse M. Hunter, Rupa Udani, Binghuang Cai, Alexander A. Morgan, Artem Sokolov, Joshua M. Stuart, Giovanni Minervini, Alexander M. Monzon, Serafim Batzoglou, Atul J. Butte, Marc S. Greenblatt, Reece K. Hart, Ryan Hernandez, Tim J. P. Hubbard, Scott Kahn, Anne O’Donnell-Luria, Pauline C. Ng, John Shon, Joris Veltman, Justin M. Zook

**Affiliations:** https://genomeinterpretation.org

## Abstract

**Background:**

The Critical Assessment of Genome Interpretation (CAGI) aims to advance the state-of-the-art for computational prediction of genetic variant impact, particularly where relevant to disease. The five complete editions of the CAGI community experiment comprised 50 challenges, in which participants made blind predictions of phenotypes from genetic data, and these were evaluated by independent assessors.

**Results:**

Performance was particularly strong for clinical pathogenic variants, including some difficult-to-diagnose cases, and extends to interpretation of cancer-related variants. Missense variant interpretation methods were able to estimate biochemical effects with increasing accuracy. Assessment of methods for regulatory variants and complex trait disease risk was less definitive and indicates performance potentially suitable for auxiliary use in the clinic.

**Conclusions:**

Results show that while current methods are imperfect, they have major utility for research and clinical applications. Emerging methods and increasingly large, robust datasets for training and assessment promise further progress ahead.

**Supplementary Information:**

The online version contains supplementary material available at 10.1186/s13059-023-03113-6.

## Background

Rapidly accumulating data on individual human genomes hold the promise of revolutionizing our understanding and treatment of human disease [1, 2]. Effectively leveraging these data requires reliable methods for interpreting the impact of genetic variation. The DNA of unrelated individuals differs at millions of positions [3], most of which make negligible contribution to disease risk and phenotypes. Therefore, interpretation approaches must be able to identify the small number of variants with phenotypic significance, including those causing rare disease such as cystic fibrosis [4], those contributing to increased risk of cancer [5] or acting as cancer drivers [6], those contributing to complex traits such as type II diabetes [7], and those affecting the response of individuals to drugs such as warfarin [8]. Identifying the relationship between variants and phenotype can also lead to new biological insights and new therapeutic strategies. Until recently, interpretation of the role of specific variants has either been acquired by empirical observations in the clinic, thus slowly accumulating robust knowledge [9], or by meticulous and often indirect in vitro experiments whose interpretation may be challenging. Computational methods offer a third and potentially powerful approach, and over one hundred have been developed [10], but their power, reliability, and clinical utility have not been established. Some computational approaches aim to directly relate sequence variation to disease or other organismal phenotypes; for example, using evolutionary conservation [11]. Other methods suggest impact on disease only secondarily, as they aim to relate genetic variants to functional properties such as effects on protein stability [12], intermolecular interactions [13], splicing [14], expression [15], or chromatin organization [16].

The Critical Assessment of Genome Interpretation (CAGI) is an organization that conducts community experiments to assess the state-of-the-art in computational interpretation of genetic variants. CAGI experiments are modeled on the protocols developed in the Critical Assessment of Structure Prediction (CASP) program, [17] adapted to the genomics domain. The process is designed to assess the accuracy of computational methods, highlight methodological innovation and reveal bottlenecks, guide future research, contribute to the development of new guidelines for clinical practice, and provide a forum for the dissemination of research results. Participants are periodically provided with sets of genetic data and asked to relate these to unpublished phenotypes. Independent assessors evaluate the anonymized predictions, promoting a high level of rigor and objectivity. Assessment outcomes together with invited papers from participants have been published in special issues of the journal *Human Mutation* [18, 19]. Since CAGI has stewardship of genetic data from human research participants, an essential part of the organizational structure is its Ethics Forum composed of ethicists and researchers, together with patient advocates. Further details are available at https://genomeinterpretation.org/.

**Fig. 1:**
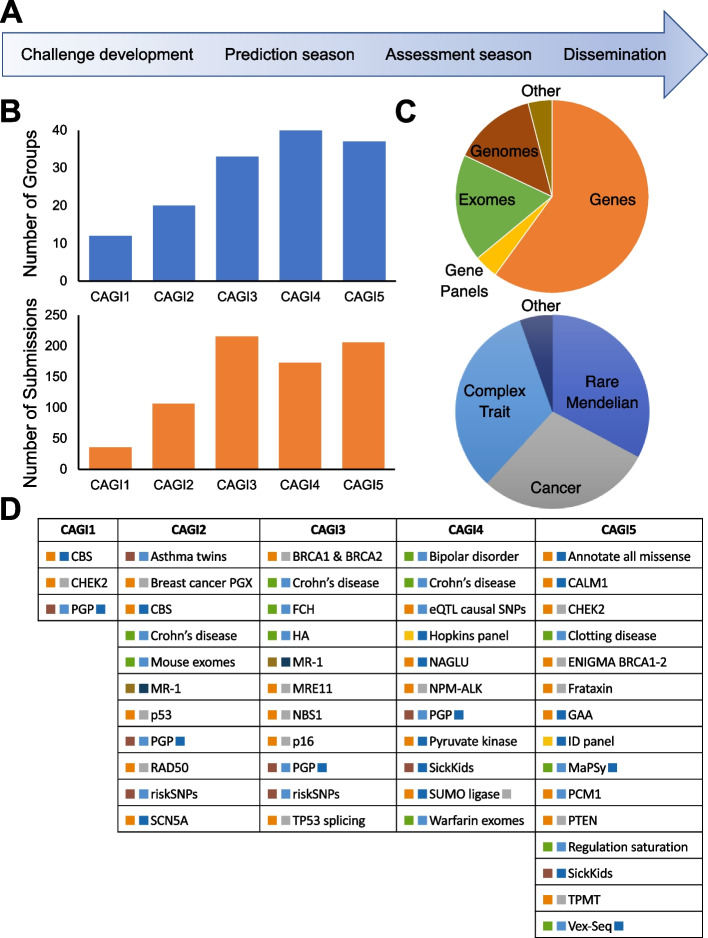
CAGI timeline, participation, and range of challenges.** A** Stages in a round of CAGI, typically extending over 2 years. Each round includes a set of challenges with similar timelines. **B** Number of participating unique groups (in blue) and submissions (in orange) across CAGI rounds. **C** Scale of the genetic data (top) and phenotypic characterization (bottom) of CAGI challenges. Some challenges belong to more than one category and are included more than once. **D** CAGI challenges, listed by round. Coloring is by scale of genetic data and phenotypic characterization according to **C**. See Supplemental Table 1 for more details

Over a period of a decade, CAGI has conducted five rounds of challenges, 50 in all, attracting 738 submissions worldwide (Fig. 1, Additional file 1: Table S1, and Additional file 1). Challenge datasets have come from studies of variant impact on protein stability [20, 21] and functional phenotypes such as enzyme activity [22, 23], cell growth [24], and whole-organism fitness [25], with examples relevant to rare monogenic disease [26], cancer [27], and complex traits [28, 29]. Variants in these datasets have included those affecting protein function, gene expression, and splicing and have comprised single base changes, short insertions or deletions (indels), as well as structural variation. Genomic scale has ranged from single nucleotides to complete genomes, with inclusion of some complementary multiomic and clinical information (Fig. 1).

In this work, we analyze the first decade of CAGI challenges in a consistent clinically relevant framework, and we identify emergent themes and unifying principles across the range of genome variation interpretation. Results are presented from three perspectives to provide (i) the clinical community with an assessment of the usefulness and limitations of computational methods, (ii) the biomedical research community with information on the current state-of-the-art for predicting variant impact on a range of biochemical and cellular phenotypes, and (iii) the developers of computational methods with data on method performance with the aim of spurring further innovation. This latter perspective is particularly important because of the recent successes of artificial intelligence approaches in related fields [30, 31]. For each theme, specific examples of performance are provided, based on particular ranking criteria. As always in CAGI, these should not be interpreted as identifying winners and losers––other criteria might result in different selections. Further, these selections were made by authors of this paper, some of whom have been CAGI participants, rather than by independent assessors. However, the examples shown are consistent with the assessors’ earlier rankings.

## Results

### Biochemical effect predictions for missense variants are strongly correlated with the experimental data, but individual predicted effect size accuracy is limited

The pathogenicity of missense variants implicated in monogenic disease and cancer is often supported by in vitro experiments that measure effects on protein activity, cell growth, or various biochemical properties [32]. Thirteen CAGI challenges have assessed the ability of computational methods to estimate these functional effects using datasets from both high- and low-throughput experimental assays, and ten of these have been reanalyzed here.

**Fig. 2:**
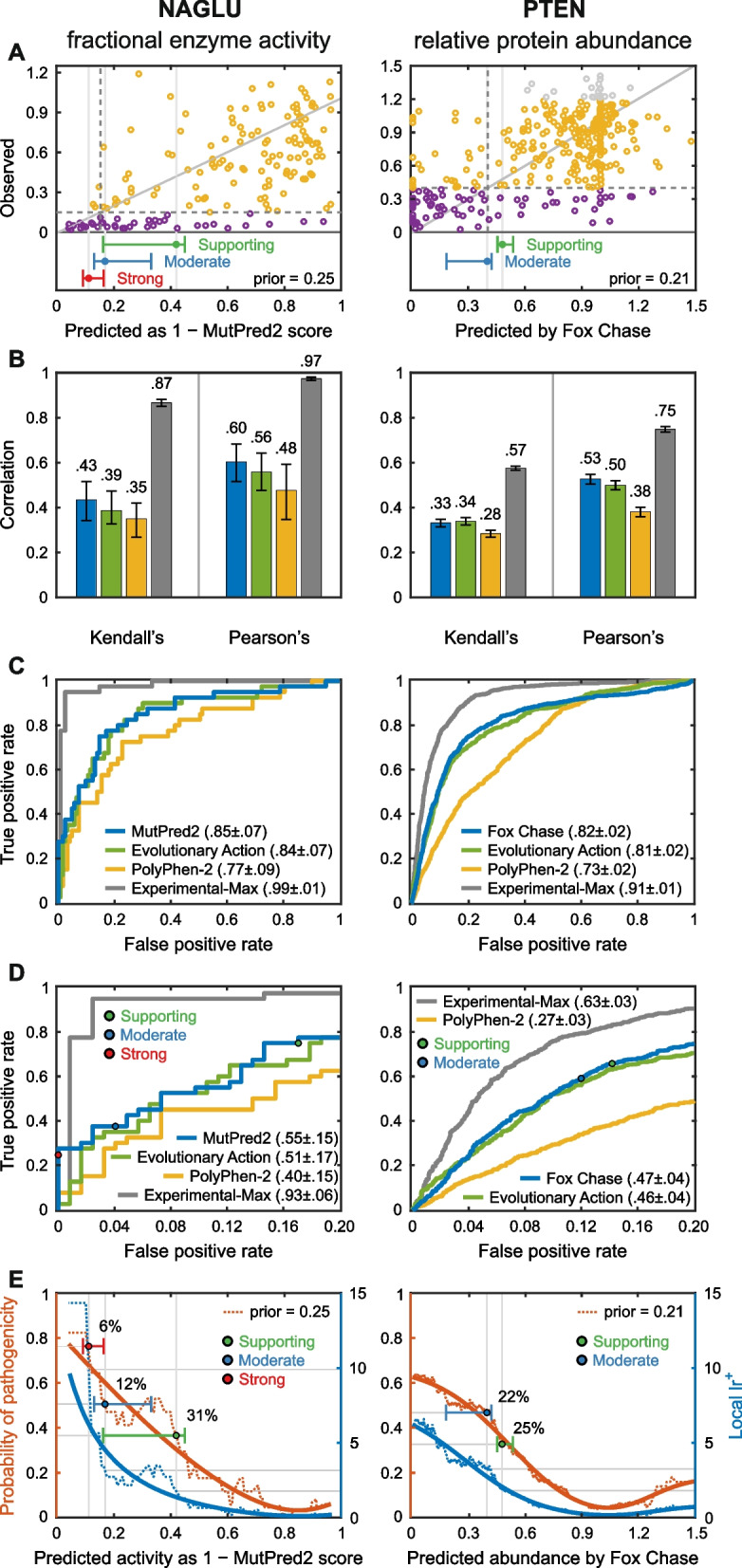
Predicting the effect of missense variants on protein properties: Results for two example CAGI challenges. Each required estimation of continuous phenotype values, enzyme activity in a cellular extract for NAGLU and intracellular protein abundance for PTEN, for a set of missense variants. Selection of methods is based on the average ranking over four metrics for each participating method: Pearson’s correlation, Kendall’s tau, ROC AUC, and truncated ROC AUC; see “Methods” for definitions. **A** Relationship between observed and predicted values for the selected method in each challenge. “Benign” variants are yellow and “pathogenic” are purple (see text). The diagonal line represents exact agreement between predicted and observed values. Dashed lines show the thresholds for pathogenicity for observed (horizontal) and predicted biochemical values (vertical). For NAGLU, below the pathogenicity threshold, there are 12 true positives (lower left quadrant) and three false positives (upper left quadrant), suggesting a clinically useful performance. Bars below each plot show the boundaries for accuracy meeting the threshold for Supporting (green), Moderate (blue), and Strong (red) clinical evidence, with 95% confidence intervals. **B** Two measures of overall agreement between computational and experimental results, for the two selected performing methods and positive and negative controls, with 95% confidence intervals. An older method, PolyPhen-2, provides a negative control against which to measure progress over the course of the CAGI experiments. Estimated best possible performance is based on experimental uncertainty and provides an empirical upper limit positive control. The color code for the selected methods is shown in panel **C**. **C** ROC curves for the selected methods with positive and negative controls, using estimated pathogenicity thresholds. **D** Truncated ROC curves showing performance in the high true positive region, most relevant for identifying clinically diagnostic variants. The true positive rate and false positive rate thresholds for the Supporting, Moderate, and Strong evidential support are shown for one selected method. **E** Estimated probability of pathogenicity (left *y*-axis) and positive local likelihood ratio (right *y*-axis) as a function of one selected method’s score. Predictions with probabilities over the red, blue, and green thresholds provide Strong, Moderate, and Supporting clinical evidence, respectively. Solid lines show smoothed trends. Prior probabilities of pathogenicity are the estimated probability that any missense variant in these genes will be pathogenic. For NAGLU, the probabilities of pathogenicity reach that needed for a clinical diagnosis of “likely pathogenic.” For predicted enzyme activity less than 0.11, the probability provides Strong evidence, below 0.17 Moderate evidence, and below 0.42, Supporting evidence. The percent of variants encountered in the clinic expected to meet each threshold are also shown. Performance for PTEN shows that the results are consistent with providing Moderate and Supporting evidence levels for some variants

Figure 2 shows selected results for two challenges, each with a different type of biochemical effect. In the NAGLU challenge [22], participants were asked to estimate the relative enzyme activity of 163 rare missense variants in N-acetyl-glucosaminidase found in the ExAC database [33]. In the PTEN challenge [20], participants were asked to estimate the effects of a set of 3716 variants in the phosphatase and tensin homolog on the protein’s stability as measured by relative intracellular protein abundance in a high-throughput assay [34]. For both challenges, the relationship between estimated and observed phenotype values shows high scatter (Fig. 2A). There is modest improvement with respect to a well-established older method, PolyPhen-2 [35], which we consider a baseline. This is a trend consistently seen in other missense challenges (Additional file 2: Table S2). How much of this improvement is due to the availability of larger and more reliable training sets rather than methodological improvements is unknown. Consistent with the scatter plots, there is moderate agreement between predicted and experimental values as measured by Pearson’s correlation and Kendall’s tau (Fig. 2B).

Over all ten analyzed missense functional challenges (Additional file 3: Table S3, Additional file 1: Figures S1-S6), Pearson’s correlation for the selected methods ranges between 0.24 and 0.84 (average correlation $\overline{r }$ = 0.55) and Kendall’s tau ranges between 0.17 and 0.63 ($\overline{\tau }$ = 0.40), both showing strong statistical significance over the random model ($\overline{r }$ = 0, $\overline{\tau }$ = 0). The PolyPhen-2 baseline achieves $\overline{r }$ = 0.36 and $\overline{\tau }$ = 0.23. Direct agreement between observed and predicted values is measured by ${R}^{2}$, which is 1 for a perfect method and 0 for a control method that assigns the mean of the experimental data for every variant. For NAGLU, the highest ${R}^{2}$ achieved is 0.16, but for PTEN it is only − 0.09. Over the ten biochemical challenges, the highest ${R}^{2}$ value ranges between − 0.94 and 0.40, with an average of − 0.19. The relatively poorer performance shown by this criterion compared with Pearson’s and Kendall’s correlation metrics suggests that the methods are often not well calibrated to the experimental value, reflecting the fact that they are rarely designed for predictions of continuous values and scales of this kind. Overall, performance is far above random but modest in terms of absolute accuracy.

#### Diversity of methods

A diverse set of methods was used to address the biochemical effect challenges, varying in the type of training data, input features, and statistical framework. Most were trained on pathogenic versus presumed benign variants [10, 36]. At first glance, a binary classification approach appears ill-suited to challenges which require prediction across a full range of phenotype values. In practice, function and pathogenicity are related [37], and so these methods performed as well as the few trained specifically to identify alteration of function [38].

Many methods are based on measures that reflect the evolutionary fitness of substitutions and population dynamics, rather than pathogenicity or functional properties. The relationship between fitness, pathogenicity, and function is complex, perhaps limiting performance. To partly address this, some methods also exploit functional roles of specific sequence positions, particularly by utilizing UniProtKB annotations and predicted structural and functional properties [38–41].

Current methods typically address the effect of single variants in isolation from possible epistatic factors although many apparently monogenic diseases are influenced by modifier variants. For example, severity of cystic fibrosis is affected by several genes beyond CFTR [4], and studies of loss of function variants in general populations revealed cases where a strong disease phenotype is expected but not observed, implying the presence of compensating variants [42].

Despite the broad range of algorithms, training data, features, and the learning setting, there is a strong correlation between results of the leading methods (Pearson’s correlation ranges from 0.6 to 0.9), almost always stronger than the correlation between specific methods and experiment (Additional file 1: Figure S8). The level of inter-method correlation is largely unrelated to the level of correlation with experiment, which varies widely from about 0.24 (CALM1) to 0.6 (NAGLU). Why correlation between methods is stronger than with experiment is unclear, though it may be affected by the relatedness of functional disruption, evolutionary conservation, and pathogenicity as well as common training data and experimental bias. The assessor for the NAGLU challenge identified 10 variants where experiment disagrees strongly with predicted values for all methods [22]. When these are removed, the correlation between the leading methods’ results and experiment increases from 0.6 to 0.73 (Additional file 1: Figure S8), although it is still lower than the correlation between the two leading methods (0.82), of which, surprisingly, one is supervised [40] and the other is not [43]. It could be that these 10 variants are cases where the computational methods systematically fail, or it could be that most are some form of experimental artifact. In situations like this, follow-up experiments are needed.

#### Structure-informed approaches

Some methods use only biophysical input features, and in some cases are trained on the effect of amino acid substitutions on protein stability, rather than pathogenicity or functional impact. Benchmarking suggests that a large fraction of rare disease-causing and cancer driver missense mutations act through destabilization [44, 45], so there is apparently considerable potential for these approaches. These methods have been effective on challenges directly related to stability, being selected as first and second for the PTEN and TMPT protein abundance challenges and first for the Frataxin change of free energy of folding challenge. They have been among top performers in a few other challenges, sometimes in combination with sequence feature methods, for example, cancer drivers in CDKN2A and rescue mutations in TP53 [21]. Generally, however, these methods, along with the structure-based machine learning methods, have not been as successful as expected compared to the methods that are primarily sequence-based. Three factors may improve their performance in future. First, better combination with the sequence methods will likely mitigate the problem of false negatives; that is, pathogenic variants that are not stability related. Second, until recently, use of structure has been restricted by low experimental structural coverage of the human proteome (only about 20% of residues). Because of recent dramatic improvements in the accuracy of computed protein structures [46], variants in essentially all residues in ordered structural regions are now amenable to this approach. Third, better estimation of the effect of missense mutations on stability [47] should improve accuracy. An advantage of biophysical and related methods is that they can sometime provide greater insight into underlying molecular mechanisms (Additional file 1: Figure S13).

Domain-level information had the potential to deliver improved performance in other instances, such as CBS, where the heme-binding domain present in humans was absent from the yeast ortholog, and RAD50, for which assessment showed that restricting predictions of deleteriousness to the specific domain involved in DNA repair would have substantially improved the accuracy of several methods.

### Computational methods can substantially enhance clinical interpretation of missense variants

The most direct test of the clinical usefulness of computational methods is to assess their ability to correctly assign pathogenic or benign status for clinically relevant variants. CAGI challenges have addressed this for rare disease variant annotations and for germline variants related to cancer risk.

#### Results for predicting the biochemical effects of missense mutations inform clinical applications

For some biochemical challenges, it is possible to relate the results to clinical utility of the methods. For NAGLU, some rare variants in the gene cause recessive Sanfilippo B disease. Disease strongly correlates with variants conferring less than 15% enzyme activity [22], allowing variants in the study to be classified as pathogenic or benign on that basis (purple and yellow circles in Fig. 2A). Figure 2A shows that 12 out of the 15 variants with less than 15% predicted activity using the selected method also have less than 15% experimental activity, suggesting high positive predictive value and clinical usefulness for assigning pathogenicity. On the other hand, 28 of the 40 variants with measured activity below 15% are predicted to have higher activity so there are also false negatives. For PTEN, information on the relationship to disease is less well established, but data fall into low and high abundance distributions [20], and the assessor suggested a pathogenicity threshold at the distribution intersection.

Performance in correctly classifying variants as pathogenic is often represented by ROC curves (Fig. 2C), showing the tradeoff between true positive (*y*-axis) and false positive (*x*-axis) rates as a function of the threshold used to discretize the phenotype value returned by a prediction method, and summarized by the area under that curve (AUC). The selected methods return AUCs greater than 0.8 for both challenges. Over all reanalyzed biochemical effect challenges, the top AUC ranges from 0.68 to 1.0, with an average of $\overline{{\text{AUC}} }$ = 0.83, and with high statistical significance over a random model (${\text{AUC}}$ = 0.5). The PolyPhen-2 baseline has $\overline{{\text{AUC}} }=0.74$, see Additional file 3: Table S3. However, all models fall well short of the empirical limit ($\overline{{\text{AUC}} }$ = 0.98) estimated from variability in experimental outputs. Because the experimental uncertainties are based on technical replicates, the experimental AUCs are likely overestimated, so it is difficult to judge how much further improvement might be possible. The full ROC curve areas provide a useful metric to measure the ability of the methods to separate pathogenic from other variants. In a clinical setting though, the left portion of the curve is often the most relevant; that is, the fraction of pathogenic variants identified without incurring too high a level of false positives, where the level of tolerated false positives is application dependent. Figure 2D uses truncated ROC curves to show the performance in this region, with the selected methods’ AUCs reaching 0.55 for NAGLU and 0.47 for PTEN. The smaller value for the PTEN truncated ROC curve AUC reflects the higher fraction of false positives at the left of the PTEN scatter plot, particularly those variants predicted to have near-zero protein abundance but with high observed values.

For use in the clinic, the quantity of most interest is the probability that a variant is diagnostic of disease (i.e., can be considered pathogenic), given the available evidence. In addition to the information provided by a computational method, initial evidence is also provided by knowledge of how likely any variant in a particular gene is to be diagnostic of the disease of interest [48]. For example, for NAGLU, about 25% of the rare missense variants in ExAC were found to have less than 15% enzyme activity, [49] suggesting that there is an approximately 25% prior probability that any rare missense variant found in the gene will be pathogenic (a prior odds of pathogenicity of 1:3). To obtain the desired posterior probability of pathogenicity, which is also the local positive predictive value at the score $s$ returned by a method, one can use a standard Bayesian odds formulation [50]

$$\mathrm{posterior}\;\mathrm{odds}\;\mathrm{of}\;\mathrm{pathogenicity}=\text{lr}^+\times\mathrm{prior}\;\mathrm{odds}\;\mathrm{of}\;\mathrm{pathogenicity},$$

where the local positive likelihood ratio, ${{\text{lr}}}^{+}$, is the slope of the ROC curve at the score value $s$; see “Methods” for a formal discussion.

Figure 2E shows ${{\text{lr}}}^{+}$ and the posterior probability of pathogenicity for NAGLU and PTEN. For NAGLU, at low predicted enzyme activities ${{\text{lr}}}^{+}$ rises sharply to about 15. The corresponding posterior probability is 0.8. For PTEN, ${{\text{lr}}}^{+}$ reaches a value of about 6. Using a pathogenicity prior of 0.21 (Additional file 1), the corresponding posterior probability of pathogenicity is 0.6. ACMG/AMP sequence variant interpretation guidelines recommend a probability of pathogenicity of ≥ 0.99 to label a variant “pathogenic” and ≥ 0.90 to label one “likely pathogenic”, the thresholds for clinical action [32, 51, 52]. So for these and other biochemical challenges, the computational evidence alone is not sufficiently strong to classify the variants other than as variant of uncertain significance.

However, the clinical guidelines integrate multiple lines of evidence to contribute to meeting an overall probability of pathogenicity threshold, so that it is not necessary (or indeed possible) for computational methods alone to provide a pathogenicity assignment. The guidelines provide rules that classify each type of evidence as Very Strong, Strong, Moderate, and Supporting [32]. For example, a null mutation in a gene where other such mutations are known to cause disease is considered Very Strong evidence, while at the other extreme, a computational assignment of pathogenicity for a missense mutation is currently considered only Supporting. Although these guidelines were originally defined in terms of evidence types, Tavtigian et al. [52] have shown that the rules can be approximated using a Bayesian framework, with each threshold corresponding to reaching a specific positive likelihood ratio; e.g., ${{\text{lr}}}^{+}=2.08$ for Supporting evidence when the prior probability is 0.1 (Methods). The resulting thresholds for each level of evidence are shown below the scatter plots in Fig. 2A and in the posterior probability of pathogenicity plots in Fig. 2E. For NAGLU, for the selected method, predicted enzyme activities lower than 0.11 correspond to Strong evidence, below 0.17 to Moderate, and below 0.42 to Supporting. These thresholds correspond to approximately 31% of rare variants in this gene providing Supporting evidence, 12% Moderate, and 6% Strong. The top-performing methods for the ten biochemical missense challenges all reach Supporting, and sometimes Moderate and Strong evidential support (Additional file 1: Figures S1-S6, Additional file 2: Table S2 and Additional file 3: Table S3). These results are encouraging in that they suggest this framework can supply a means of quantitatively evaluating the clinical relevance of computational predictions and that under appropriate circumstances, computational evidence can be given more weight than at present. The next section explores these properties further.

#### Identifying rare disease variants

The ClinVar [9] and HGMD [53] databases provide an extensive source of rare disease-associated variants against which to test computational methods. A limitation is that most methods have used some or all of these data in training, making it difficult to perform unbiased assessments. The prospective “Annotate All Missense” challenge assessed the accuracy of those predictions on all missense variants that were annotated as pathogenic or benign in ClinVar and HGMD after May 2018 when predictions were recorded, through December 2020, so avoiding training contamination. All predictions directly submitted for the challenge as well as all precomputed predictions deposited in the dbNSFP database [54] before May 2018 (dbNSFP v3.5) were evaluated, predictions from a total of 26 groups.

**Fig. 3:**
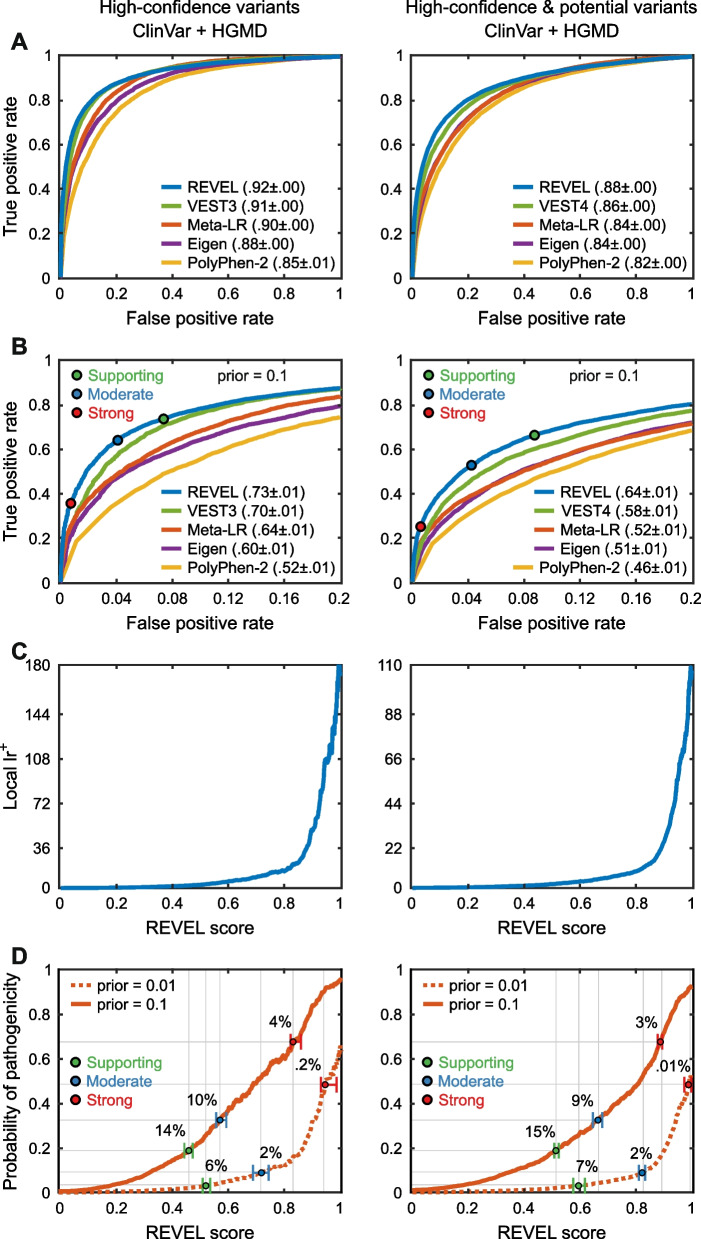
Performance of computational methods in correctly identifying pathogenic variants in the two principal rare disease variant databases, HGMD and ClinVar. The left panels show data for variants labeled as “pathogenetic” in ClinVar and “DM” in HGMD together with “benign” in ClinVar. The right panels add variants labeled as “likely pathogenic” and “likely benign” in ClinVar as well as “DM?” in HGMD. Meta and single method examples were selected on the basis of the average ranking of each method for the ROC and truncated ROC AUCs. See Additional file 1 for more details and selection criteria. **A** ROC curves for the selected metapredictors and single methods, together with a baseline provided by PolyPhen-2. Particularly for pathogenic variants alone, impressively high ROC areas are obtained, above 0.9, and there is a substantial improvement over the older method’s performance. **B** Blowup of the left-hand portion of the ROC curves, most relevant to high confident identification of pathogenic variants. Clinical thresholds for Supporting, Moderate, and Strong clinical evidence are shown. **C** Local positive likelihood ratio as a function of the confidence score returned by REVEL. Very high values (> 100) are obtained for the most confident pathogenic assignments. **D** Local posterior probability of pathogenicity; that is, probability that a variant is pathogenic as a function of the REVEL score for the two prior probability scenarios. For a prior probability of 0.1, typical of a single candidate gene situation (solid line) and database pathogenic and benign variants (left panel) the highest-scoring variants reach posterior probability above 0.9, strong enough evidence for a clinical assignment of “likely pathogenic.” In both panels, variants with a score greater than 0.45 provide Supporting clinical evidence (green threshold), and scores greater than 0.8 provide Strong evidence (red threshold). The estimated % of variants encountered in a clinical setting expected to meet each threshold are also shown. For example, about 14% of variants provide Supporting evidence. Dotted lines show results obtained with a prior probability of 0.01

All selected methods, including PolyPhen-2, achieved high AUCs, ranging from 0.85 to 0.92 for separating “pathogenic” from “benign” variants, and only slightly lower values (maximum AUC 0.88) when “likely pathogenic” and “likely benign” are included (Fig. 3A). The two metapredictors, REVEL [55] and Meta-LR [56], tools that incorporate predictions from multiple other methods, perform slightly better than primary methods, although VEST3 and VEST4 [39] outperformed Meta-LR. There is a substantial improvement over the performance of PolyPhen-2, especially in the left part of the ROC curve (Fig. 3B), though as with the biochemical effect challenges, some of that may be due to the availability of larger and more reliable training sets. Additional file 4: Table S4 shows slightly higher performance on ClinVar pathogenic variants than HGMD; however, these resources use different criteria for assigning pathogenicity.

The lower panels in Fig. 3 show positive likelihood ratios and posterior probability of pathogenicity for a selected metapredictor, REVEL [55]. With a prior probability of pathogenicity of 0.1 (approximating the prior when examining possible pathogenic variants in one or a few genes in a diagnostic setting; see “Methods”) 14, 10, and 4% of variants reach the Supporting, Moderate, and Strong evidence thresholds, respectively. With the much smaller prior of 0.01, representative of screening for possible secondary variants, about 6% of variants will provide Supporting evidence and 2% reach Moderate. These estimates are not exact since there may be significant differences between the distribution and properties of variants in these databases and those encountered in the clinic. For example, some genes have only benign variant assignments in the databases, and these might be excluded from consideration in the clinic. Performing the analysis on only genes with both pathogenic and benign assignments slightly reduced performance—highest AUC on the confident set of variants is 0.89 instead of 0.92. The selected methods also change slightly; see Additional file 1: Figure S9. In spite of this and other possible caveats, the overall performance of the computational methods is encouraging and, as with biochemical effect challenges, suggests that the computational methods can provide greater benefit in the clinic than recognized by the current standards.

#### Identifying germline cancer risk variants

About a quarter of CAGI experiments have involved genes implicated in cancer (Fig. 1) and have included variants in BRCA1, BRAC2, PTEN, TPMT, NSMCE2 (coding for SUMO-ligase), CHEK2, the MRN complex (RAD50, MRE11, and NBS1), FXN, NPM-ALK, CDKN2A, and TP53. An additional challenge addressed breast cancer pharmacogenomics. From a cancer perspective, the most informative of these is a challenge provided and assessed by members of the ENIGMA consortium [27], using a total of 321 germline BRCA1/BRCA2 missense and in-frame indel variants. Performance on this challenge was impressively high, with four groups providing submissions that gave AUCs greater than 0.9 and two with AUCs exceeding 0.97. In the other BRCA1/BRCA2 variant challenge, the highest AUC is 0.88 on a total of 10 missense variants. The strong results may reflect the fact these are highly studied genes. More and larger scale challenges with a variety of genes are required in order to draw firm conclusions. Further details of cancer challenges are provided in Additional file 1: Figure S10 and Additional file 5: Table S5.

### Assessing methods that estimate the effect of variants on expression and splicing is difficult, but results show these can contribute to variant interpretation

Variants that regulate the abundance and isoforms of mRNA either through altered splicing or through altered rates of transcription play a significant role in disease, particularly complex traits. CAGI has included four challenges using data from high-throughput assays of artificial gene constructs, two for splicing and two for expression. For all four, evaluation of the results is limited by a combination of small effect sizes (changes larger than twofold in splicing or expression are rare in these challenges) and experimental uncertainty, but some interesting properties can be identified.

#### Splicing

The CAGI splicing challenges used data from high-throughput minigene reporter assays [57].

**Fig. 4:**
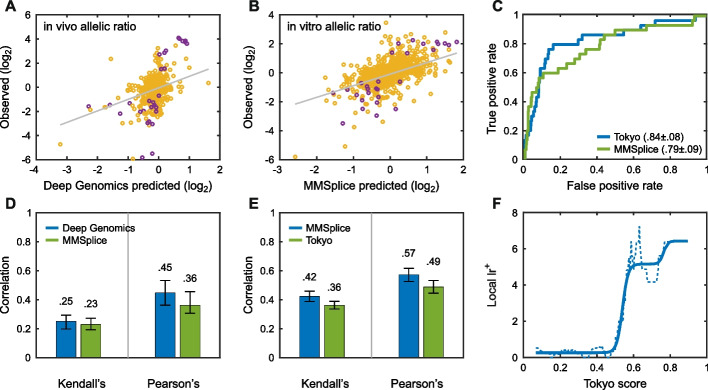
Performance of computational methods in identifying variants that affect splicing in the MaPSy challenge. Methods were selected based on the average ranking over three metrics: Pearson’s correlation, Kendall’s tau, and ROC AUC. Scatter plots, Kendell’s tau, and Pearson’s correlation results are shown for in vivo (**A**, **D**) and in vitro assays (**B**, **E**) separately. The small number of purple points in the scatter plots represent splicing fold changes greater than 1.5-fold. The ROC curve (**C**) shows performance in variant classification for the two selected methods. The maximum local positive likelihood ratio (${{\text{lr}}}^{+}$, **F**) may be large enough for use as auxiliary information, see “ Discussion” (solid line is smoothed fit to the data)

The MaPSy challenge asked participants to identify which of a set of 797 exonic single-nucleotide HGMD disease variants affect splicing and by how much. Two experimental assays were available, one in vitro on a cell extract and the other by transfection into a cell line. Only variants that produced a statistically robust change of at least 1.5-fold were considered splicing changes. Figure 4 summarizes the results. The top-performing groups achieved moderately high AUCs of 0.84 and 0.79 and the highest ${{\text{lr}}}^{+}$ is about 6. Notably, very few variants qualify as significant splicing changes, and there are inconsistences between the two assays, with a number of variants appearing to have a fold change substantially greater than 1.5 in one assay but not the other. Additionally, the experimental noise significantly overlaps with many splicing differences. For these reasons, it is unclear what maximum AUC could be achieved by a perfect method.

The Vex-Seq challenge required participants to predict the extent of splicing change introduced by 1098 variants in the vicinity of known alternatively spliced exons [57]. Additional file 1: Figure S11 shows that performance was rather weak for identification of variants that increase splicing (top AUC = 0.71), but that may be because many points classified as positive are experimental noise. There are more variants that show a statistically robust decrease in splicing, and prediction performance is correspondingly stronger (top AUC = 0.78). The assessor noted additional nuances in performance [57].

The selected high-performing method for both challenges (MMSplice [58]) decomposes the sequence surrounding alternatively spliced exons into distinct regions and evaluates each region using separate neural networks [58]. More detailed splicing results are provided in Additional file 6: Table S6.

#### Transcription

Several CAGI challenges have assessed the ability of computational methods to identify single base changes that affect the expression level of specific genes. The CAGI4 eQTL challenge assessed whether methods could find causative variants in a set of eQTL loci, using a massively parallel reporter assay [59]. Because of linkage disequilibrium and sparse sampling, variants associated with an expression difference in an eQTL screen are usually not those directly causing the observed expression change. Rather, a nearby variant will be. The challenge had two parts. Participants were asked to predict whether insertion of the section of genomic DNA around each variant position into the experimental construct produced any expression. Additional file 1: Figure S12A shows that the top-performing methods were effective at this—the largest AUC is 0.81. The second part of the challenge required participants to predict which variants affect expression levels. Here the results are much less impressive (Additional file 1: Figure S12B). The scatter plot shows a weak relationship between observed and predicted expression change and Pearson’s or Kendall’s correlation are also small. The best AUC is only 0.66 and the maximum ${{\text{lr}}}^{+}$ is about 5. Most of the experimental expression changes are small (less than twofold) and may be largely experimental noise, partly accounting for the apparent poor performance. But as the scatter plot shows, a subset of the variants with largest effects could not be identified by the top-performing method. A combination of experimental and computational factors contributed to poor performance, and more challenges of this sort are needed.

**Fig. 5:**
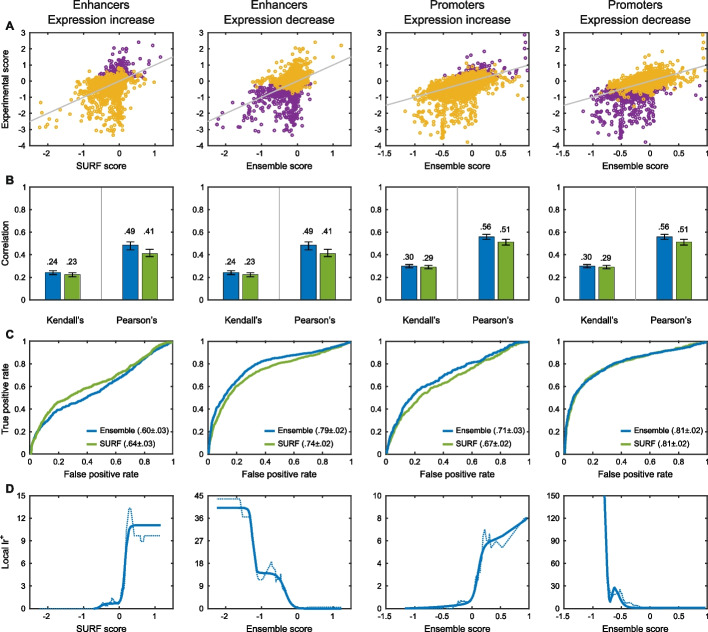
Performance on the regulation saturation expression challenge. The two left columns show performance in predicting increased (left) and decreased (right) expression in a set of enhancers (purple points represent variants that significantly change expression). The right pair of columns show equivalent results for promoters. The scatter plots (**A**) show strong performance in identifying decreases in expression (purple points), but weaker results for expression increases. Performance on promoters is stronger than on enhancers. Overlap of changed and non-changed experimental expression points suggests that experimental uncertainty reduces the apparent performance of the computational methods. Panel **B** shows correlation coefficients for selected methods. Panel **C** shows ROC curves for predicting under and overexpression. Panel **D** shows local ${{\text{lr}}}^{+}$, where the solid lines are smoothed fits to the data

The CAGI5 regulation saturation mutagenesis challenge examined the impact on expression of every possible base pair variant within five disease-associated human enhancers and nine disease-associated promoters [60]. As shown in Fig. 5, performance is stronger in promoters than enhancers and stronger for decreases in expression compared with increases. Fewer variants show experimental increases and these tend to be less well distinguished from noise. Performance for small expression changes is hard to evaluate because of overlap with experimental noise. Nevertheless, the highest AUC for promoter impact prediction is 0.81 while the highest AUC for enhancer impact prediction is 0.79, relatively respectable values. In addition, the scatter plots show that large decreases in expression are well predicted, suggesting the methods are quite informative for the most significant effects.

The CAGI splicing and expression challenges are not as directly mappable to disease and clinical relevance as in some other challenge areas. Variants have been a mixture of common and rare and the use of artificial constructs in high-throughput experiments limits relevance of challenge performance in the whole-genome context. Nevertheless, the results do have potential applications. In complex trait disease genome-wide association studies (GWAS), the variants found to be associated with a phenotype are usually not those causing the effect. Identifying the functional variants is not straightforward, and current regulatory prediction methods can provide hypotheses as to possible effects on expression or splicing.

### CAGI participants identified diagnostic variants that were not found by clinical laboratories

A major goal of CAGI is to test the performance of computational methods under as close to clinical conditions as possible. In the area of rare disease diagnosis, four challenges have addressed this by requiring participants to identify diagnostic variants in sets of clinical data. The Johns Hopkins and intellectual disability (ID) challenges employed diagnostic panels, covering a limited set of candidate genes in particular disease areas. As compared with genome-wide data, diagnostic panels inherently restrict the search to only variants belonging to a known set of relevant genes.

For a number of genetically undiagnosed cases in the Johns Hopkins panel, CAGI participants found high-confidence deleterious variants in genes associated with a different disease from that reported, suggesting physicians may have misdiagnosed the symptoms [61]. However, because of the clinical operating procedures of the diagnostic laboratory, it has not been possible to further investigate these cases. In the ID panel, some plausible calls were made on novel variants that had not been reported to the patient partly because the majority of standard computational methods returned assignments of “benign” [62].

The two SickKids challenges (SickKids4 and SickKids5) were based on whole-genome sequence data for children with rare diseases from the Hospital for Sick Children in Toronto. These are all cases that were undiagnosed by the state-of-the-art SickKids pipeline [26], and so were particularly challenging compared with those normally encountered in the clinic. In the SickKids4 challenge, variants proposed by challenge participants were deemed diagnostic by the referring physicians for two of the cases in part due to matching detailed phenotypes. This was the first instance of the CAGI community directly contributing in the clinic. In SickKids5, two of the highest confidence nominated diagnostic variants provided correct genome-patient matches. While not meeting ACMG/AMP criteria for pathogenicity [32], these were considered interesting candidates for further investigation, again potentially resolving previously intractable cases.

These clinical challenges required participants to develop full analysis pipelines, including quality assessment for variant calls, proper inclusion of known pathogenetic variants from databases such as HGMD [53] and ClinVar [9], and an evaluation scheme for weighing the evidence. The SickKids challenges also required compilation of a set of candidate genes. Varying success in addressing these factors will have influenced the results, so it is not possible to effectively compare the core computational methods. Overall, current approaches have limitations in this setting––they tend to ignore or fail to reliably evaluate synonymous and noncoding variants; if the relevant gene is not known its variants will usually not be examined; and data for epigenetic causes are not available. Nevertheless, the CAGI results for these challenges again make it clear that current state-of-the-art computational approaches can make valuable contributions in real clinical settings.

### Complex trait interpretation is often complicated by confounders in the data

Many common human diseases, such as Alzheimer’s disease, asthma, and type II diabetes, are complex traits and as with monogenic disorders, genetic information should in principle be useful for both diagnosis and prognosis. Individual response to drugs (pharmacogenomics) also often has a complex trait component. Complex traits have relatively small contributions from each of many variants, collectively affecting a broad range of molecular mechanisms, including gene expression, splicing, and multiple aspects of protein function. Environmental factors also play a substantial role so that phenotype prediction based on genetic information alone has inherently limited accuracy. Also, most CAGI complex trait challenges have been based on exome data, whereas at many GWAS risk loci lie outside coding regions [63]. To some extent, the status of relevant common variants not present in the exome data can be imputed on the basis of linkage disequilibrium, but this places an unclear limit on achievable accuracy. Limited or no availability of training data also restricted method performance and phenotypes tend to be less precise than for other types of disease. Altogether, these factors make this a difficult CAGI area. Nevertheless, these challenges have been informative and have drawn new investigators into the rapidly developing area of Polygenic Risk Score (PRS) estimation [64]. One challenge, CAGI4 Crohn’s, has yielded apparently robust conclusions on the performance of methods in this area.

#### Crohn’s disease (CAGI4)

**Fig. 6:**
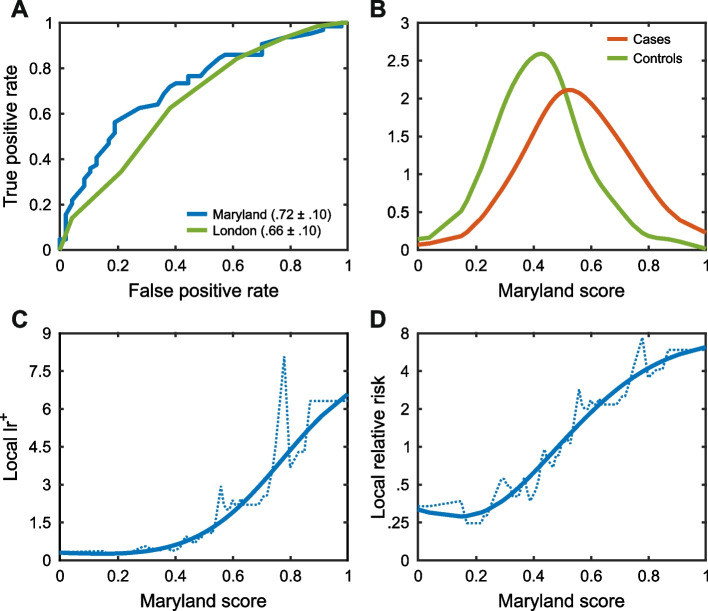
Identifying which of a set of individuals are most at risk for Crohn’s disease, given exome data. Examples were selected on the basis of ranking by ROC AUC. **A** ROC curves for two selected methods. Statistically significant but relatively low ROC areas are obtained. **B** Distributions of disease prediction scores for individuals with the disease (red) and without (green) for the method with the highest AUC (kernel density representation of the data). **C** Local positive likelihood ratio (${{\text{lr}}}^{+}$) as a function of prediction score for the method with the highest AUC. **D** Relative risk of disease (log_2_ scale), compared to that in the general population as a function of prediction score. Individuals with the lowest risk scores have approximately 1/3 the average population risk, while those with the highest scores have risk exceeding fourfold the average, a 12-fold total range. Depending on the disease, identifying individuals with higher than threefold the average risk may be sufficient for clinical action

Participants were provided with exome data for 111 individuals and asked to identify the 64 who had been diagnosed with Crohn’s disease. A variety of computational approaches were used, including clustering by genotypes, analysis of variants in pathways related to the disease, and evaluation of SNPs in known disease-associated loci. The highest-scoring method (AUC 0.72; Fig. 6A) used the latter approach together with conventional machine learning, and trained on data from an earlier GWAS [65]. Fig. 6B shows case and control score distributions for that method. A perfect method would have no distribution overlap. These results are far from that, but there is clear signal at the extremes, and as Fig. 6C shows, that translates into a positive likelihood ratio with an approximately 20-fold range (0.3 to 6), only a little lower than that obtained for the biochemical effect and clinical missense challenges. With a prior probability of disease of 1.3% [66], relative risk (see “Methods”) also has a range of about 20-fold (Fig. 6D), with the highest-risk individuals having sixfold higher risk compared to that estimated for the population average. For some complex trait diseases, for example coronary heart disease [67], this is discriminatory enough to support clinical action, and for many diseases would provide a valuable additional factor to more standard risk measures such as age and sex. Newer PRS methods, which aim to incorporate many weak contributions from SNPs, were not evaluated in this CAGI challenge.

Other complex trait CAGI challenges (Additional file 1) revealed batch effects (CAGI2 Crohn’s and bipolar disorder) and population structure effects (CAGI3 Crohn’s) in the data, leaked clinical data (Warfarin and VET challenges), or discrepancies between self-reported traits and those predicted from genetic data (PGP challenges), thereby complicating assessment. Performance in matching genomes and disease phenotypes, an additional component in some challenges, was poor. Additional complex trait challenge results are provided in Additional file 7: Table S7.

### The CAGI Ethics Forum has guided responsible data governance

Data used in CAGI challenges are diverse in terms of sensitivity (e.g., with respect to participant reidentification risk, potential for stigmatization, potential impact of pre-publication data disclosure), collected under a broad variety of participant consent understandings and protection frameworks, and analyzed by predictors with varying degrees of familiarity with local and international biomedical regulations. This heterogeneity calls for a nuanced approach to data access and the tailored vetting of CAGI experiments. The CAGI Ethics Forum was launched in 2015 to proactively address these concerns. Incorporating input from bioethicists, researchers, clinicians, and patient advocates, it has developed policies for responsible data governance (e.g., assisting in revision of the general CAGI data use agreement, to safeguard human data and also protect all CAGI participants, including data providers for unpublished data), cautioned against overinterpretation of findings (e.g., highlighting the contribution of social and environmental risk factors to disease, and the potential negative consequences, such as stigma, of associating particular disease variants with a specific population), and provided input on a variety of guidelines and procedures, including CAGI’s participant vetting process (e.g., how to identify a bona fide researcher) and a system of tiered access conditions for datasets, depending on their sensitivity. Future directions include investigating the scalability of current user validation and data access models, exploring implications for family members of unexpected challenge findings, discussing policies to ensure proper credit attribution for constituent primary methods used by metapredictors, and identifying additional means of ensuring accountability options with respect to responsible data sharing.

## Discussion

Over CAGI’s first decade, five rounds of CAGI challenges have provided a picture of the current state-of-the-art in interpreting the impact of genetic variants on a range of phenotypes and provided a basis for the development of improved methods as well as for more calibrated use in clinical settings.

A key finding is that for most missense challenges it is possible to relate phenotype values to a pathogenicity threshold, and so deduce potential performance in a clinical setting, particularly for rare Mendelian diseases. The results suggest that such computational methods are generally more reliable than recognized in the current clinical guidelines [32]. Several challenges have directly assessed the usefulness of variant impact prediction under clinical conditions, highlighting the fact that successful application in the clinic requires integration of the computational methods into a comprehensive pipeline.

Computational challenges and methods for identifying the effects of splicing and regulatory variants have been less well represented and issues with data availability have limited insights. Nevertheless, the results suggest potential for providing evidence for informing pathogenicity and offering mechanistic insights.

CAGI relies on the same factors as other critical assessment community experiments: a willingness of the relevant research groups to participate, clearly defined metrics for success, the availability of large enough and accurate enough sets of experimental data to provide a gold standard, and independent objective assessment of the predictions. Participation in CAGI has been strong in most areas and a vibrant and interactive community has developed. New researchers have been attracted to the field and new collaborations have resulted in the development of creative algorithms with broad applicability [68–70].

The biggest obstacle to clear assessment has been and continues to be data diversity and quality, a key difference between CAGI and related community endeavors. Other initiatives, such as CASP [71], deal primarily with one type of data (protein structure) and the data are usually of high quality and directly relevant to the goals of the computational methods. By contrast, CAGI deals with many different settings, including studies of biochemical effects with a broad range of phenotypes, the pathogenicity of variants both germline and somatic, clinical phenotypes, and statistical relationships. Also, while genome variant calling is reliable, it does have limits [72]. For example, in the SickKids challenges, some variants suggested as diagnostic by CAGI participants had been found to be incorrect calls and so eliminated in the clinical pipeline, using sequence validation data CAGI participants did not have access to. Adapting available data to form suitable challenges is difficult and compromises are sometimes needed to devise a challenge where the results can be objectively assessed. For example, in one of the SickKids and in the Johns Hopkins clinical challenges, assessment hinged on requiring participants to match genomes to phenotypes. But that makes it much harder to identify diagnostic variants than in the real-life situation. Conversely, challenge providers have sometimes benefited from the detailed scrutiny of their data by CAGI participants prior to its publication. In some cases, interpretation of clinical challenge results is also complicated by there being no conclusive diagnoses. Numerical assessments can also have limitations, as clinicians may often use other considerations while evaluating patients. CAGI participants have similarly sometimes used unanticipated information to improve performance on challenges. For example, in a PGP challenge requiring matching of full genome sequences to extensive phenotype profiles, a participant made use of information in the PGP project blog [73]. Though these sometimes subvert the intended challenge, in some ways this reflects what happens in a clinical setting—all relevant information is used, however obscure.

Experimental biotechnology platforms have become widely available [74] and the genomic data collection in the clinic has greatly increased. While the next generation of computational tools should benefit from these developments, they also pose new challenges. Deeper characterization of experimental approaches is needed to address data uncertainty and biases. Potential circularity between computation-assisted variant annotation and method assessment also needs to be considered. CAGI has committed to employing a diversity of evaluation metrics rather than any single primary performance indicator, and new assessment metrics may need to be introduced [75–77]. Future rounds of CAGI will address these issues by using assessment methods that mitigate or eliminate problems with data, by developing and promoting practices and standards for application of methods, by working with experimental groups to provide sufficiently large and high-quality datasets, and by effectively following up on disagreements at the intersection of high-throughput functional experiments, genetic association studies, and outputs of computational prediction methods. CAGI also plans to increase evaluation of the combinatorial effect of variants, either in single-molecule biochemical assays or in clinical applications with whole-genome studies for both rare and complex phenotypes.

## Conclusions

Results from the first decade of CAGI have highlighted current abilities and limitations of computational methods in genome interpretation and indicate future research directions. The current performance levels for missense variation in Mendelian disorders, combined with rapidly accumulating data and a recent breakthrough in protein structure prediction [71], suggest that upcoming methods should consistently achieve Strong and potentially Very Strong clinical evidence levels. Progress is also expected in method performance for other types of genome variation and complex disorders, assisted by improvements in experimental and statistical methodologies, as well as new clinical standards [78]. CAGI’s independent assessment rigorously ascertains the performance characteristics of computational methods for variant interpretation; this assessment approach therefore offers a model framework for evaluating clinical validity of diagnostics and screening. Genomic science has been tremendously advanced by policies ensuring rapid release of data, and to help promote the development and assessment of analytical methods these must be crafted to support portions of the datasets being incorporated into evaluations like CAGI. These developments will enable computational approaches to further narrow the gap between basic and clinical research, advancing our understanding over the entire breadth of genome variation.

## Methods

We describe different evaluation scenarios considered in the Critical Assessment of Genome Interpretation (CAGI), motivate the selection of performance measures, and discuss ways to interpret the results.

### Terminology and notation

Let $\left(x,y\right)\in$ $\mathcal{X}\times \mathcal{Y}$ be a realization of an input–output random vector $(X,Y)$. The input space $\mathcal{X}$ may describe variants, gene panels, exomes, or genomes in different CAGI scenarios. Similarly, the output space $\mathcal{Y}$ can describe discrete or continuous targets; e.g., it can be a binary set {pathogenic, benign} when the task is to predict variant pathogenicity, or a continuous set $[0, \infty )$ representing a percent of enzymatic activity of the wildtype protein (a mutated molecule can have increased activity compared to the wildtype) or a cell growth rate relative to that with the wildtype gene.

Let $s:\mathcal{X}\to {\mathbb{R}}$ be a predictor that assigns a real-valued score to each input and $f:\mathcal{X}\to \mathcal{Y}$ be a predictor that maps inputs into elements of the desired output space; i.e., $f$ is real-valued when predicting a continuous output and discrete when predicting a discrete output. When predicting binary outcomes (e.g., $\mathcal{Y}=\left\{0, 1\right\}$), $s(x)$ is often a soft prediction score between 0 and 1, whereas $f(x)$ can be obtained by discretizing $s(x)$ based on some decision threshold $\tau$. Scores $s(x)$ can also be discretized based on a set of thresholds ${{\{\tau }_{j}\}}_{j=1}^{m}$, as discussed later. In a binary classification scenario, $f\left(x\right)=1$ (pathogenic prediction) when $s\left(x\right)\ge \tau$ and $f\left(x\right)=0$ (benign prediction) otherwise. We shall sometimes denote a discretized binary model as ${f}_{\tau }\left(x\right)$ to emphasize that the predictor was obtained by thresholding a soft scoring function $s\left(x\right)$ at $\tau$. The target variable $Y$ can similarly be obtained by discretizing the continuous space $\mathcal{Y}$ using a set of thresholds $\{{\tau }_{k}^{\prime}\}$, that are different from ${\{\tau }_{j}\}$ used for the scoring function. In one such case, discretizing the continuous space $\mathcal{Y}$ of functional impact of an amino acid variant into {damaging, nondamaging} transforms a regression problem into classification, which may provide additional insights during assessment. With a minor abuse of notation, we will refer to both continuous and the derived discrete space as $\mathcal{Y}$. The exact nature of the target variable $Y$ and the output space will be clear from the context.

Finally, let $\mathcal{D}=\{{({x}_{i},{y}_{i})\}}_{i=1}^{n}$ be a test set containing $n$ input–output pairs on which the predictors are evaluated. Ideally, this data set is representative of the underlying data distribution and non-overlapping with the training data for each evaluated predictor. Similarly, we assume the quality of the measurement of the ground truth values $\{{{y}_{i}\}}_{i=1}^{n}$ is high enough to ensure reliable evaluation. While we took multiple steps to ensure reliable experiments and blind assessments, it is difficult to guarantee complete enforcement of either of these criteria. For example, an in vitro assay may be an imperfect model of an in vivo impact or there might be uncertainty in collecting experimental read-outs. Additionally, the notion of a representative test set may be ambiguous and cause difficulties when evaluating a model that was developed with application objectives different from those used to assess its performance in CAGI.

### Evaluation for continuous targets

Evaluating the prediction of continuous outputs is performed using three primary measures (${R}^{2}$, Pearson’s correlation coefficient, and Kendall’s tau) and two secondary measures (root mean square error and Spearman’s correlation coefficient). ${R}^{2}$ is defined as the difference between the variance of the experimental values and the mean-squared error of the predictor, normalized by the variance of the experimental values. It is also referred to as the fraction of variance of the target that is explained by the model. ${R}^{2}\in (-\infty ,1]$ is estimated on the test set $\mathcal{D}$ as

1 $${R}^{2}=1-\frac{{\sum }_{i=1}^{n}{\left(f\left({x}_{i}\right)-{y}_{i}\right)}^{2}}{{\sum }_{i=1}^{n}{\left({y}_{i}-\overline{y }\right)}^{2}},$$

where $\overline{y }=\frac{1}{n}{\sum }_{i=1}^{n}{y}_{i}$ is the mean of the target values in $\mathcal{D}$ (observe that each value ${y}_{i}$ may itself be an average over technical or biological replicates, if available in the experimental data). The ${R}^{2}$ values above 0 indicate that the predictor is better than the trivial predictor—one that always outputs the mean of the target variable—and values close to 1 are desired. The values below 0 correspond to models with inferior performance to the trivial predictor. Maximizing the ${R}^{2}$ metric requires calibration of output scores; that is, a high correlation between predictions and target values as well as the proper scaling of the prediction outputs. For example, a predictor outputting a linear transformation of the target such as $f\left(X\right)=10\cdot Y$ or a monotonic nonlinear transformation of the target such as $f\left(X\right)={\text{log}}Y$ may have a high correlation, but a low ${R}^{2}$. ${R}^{2}$, therefore, can be seen as the strictest metric used in CAGI. However, this metric can adversely impact methods outputting discretized prediction values. Such methods are preferred by some tool developers as they simplify interpretation by clinicians, experimental scientists, or other users.

In some cases, it may be useful to also report the root mean square error (RMSE), estimated here as

2 $${\text{RMSE}}=\sqrt{\frac{1}{n}{\sum }_{i=1}^{n}{\left(f({x}_{i}\right)-{y}_{i})}^{2}}.$$

RMSE can offer a useful interpretation of the performance and is provided as a secondary measure in CAGI evaluations.

The correlation coefficient between the prediction $f\left(X\right)$ and target $Y$ is defined as a normalized covariance between the prediction output and the target variable. Pearson’s correlation coefficient $-1\le r\le 1$ is estimated on $\mathcal{D}$ as

3 $$r=\frac{{\sum }_{i=1}^{n}({f}_{i}-\overline{f })({y}_{i}-\overline{y })}{\sqrt{{\sum }_{i=1}^{n}{\left({f}_{i}-\overline{f }\right)}^{2}{\sum }_{i=1}^{n}{\left({y}_{i}-\overline{y }\right)}^{2}}},$$

where ${f}_{i}=f\left({x}_{i}\right)$ and $\overline{f }=\frac{1}{n}{\sum }_{i=1}^{n}{f}_{i}$ is the mean of the predictions. Pearson’s correlation coefficient does not depend on the scale of the prediction, but it is affected by the extent of a linear relationship between predictions and the target. That is, a predictor outputting a linear transformation of the target such as $f\left(X\right)=10\cdot Y$ will have a perfect correlation. However, a monotonic nonlinear transformation of the target such as $f\left(X\right)={\text{log}}Y$ may have a relatively low $r$. Although not our main metric, we also explored Spearman’s rank correlation as a secondary metric. Spearman’s correlation is defined as Pearson’s correlation on the rankings.

We also computed Kendall’s tau, which is the probability of a concordant pair of prediction-target points linearly scaled to the $[-1, 1]$ interval instead of [0, 1]. Assuming that all prediction and target values are distinct, a pair of points $\left({f(x}_{i}),{y}_{i}\right)$ and $({f(x}_{j}),{y}_{j})$ is concordant if either ($f({x}_{i})>{f(x}_{j})$ and ${y}_{i}>{y}_{j}$) or ($f\left({x}_{i}\right)<{f(x}_{j})$ and ${y}_{i}<{y}_{j}$). Otherwise, a pair of points is discordant. Kendall’s tau was estimated on $\mathcal{D}$ as

4 $$\tau =\frac{2}{n(n-1)}{\sum }_{i=1}^{n-1}{\sum }_{j=i+1}^{n}{\text{sign}}({f(x}_{i})-{f(x}_{j}))\cdot {\text{sign}}({y}_{i}-{y}_{j}).$$

It ranges between $-1$ and 1, with 1, indicating that all pairs are concordant, 0 indicating half of the concordant pairs (e.g., a random ordering) and $-1$ indicating that all pairs are discordant. A predictor outputting a linear transformation of the target $f\left(X\right)=10\cdot Y$ and a monotonic nonlinear transformation of the target $f\left(X\right)={\text{log}}Y$ will both have a perfect tau of 1. Compared to Pearson’s correlation, Kendall’s tau can be seen as less sensitive to the scale but more sensitive to the ordering of predictions. Equation 4 is defined under the assumption that both the predictions and the outputs are unique. However, this assumption is not satisfied by all biological datasets and predictors. To address this issue, we use Kendall’s tau-b, a widely accepted correction for ties,

5 $${\tau }_{b}=\frac{{\sum }_{i=1}^{n-1}{\sum }_{j=i+1}^{n}{\text{sign}}({f(x}_{i})-{f(x}_{j}))\cdot {\text{sign}}({y}_{i}-{y}_{j})}{\sqrt{\left(\beta \left(n\right)-{\sum }_{i=1}^{T}\beta \left({u}_{i}\right)\right)\left(\beta \left(n\right)-{\sum }_{i=1}^{S}\beta \left({v}_{i}\right)\right)}},$$

where $$\beta \left(n\right)=n\left(n-1\right)/2$$, $${u}_{i}$$ $$(v_i)$$ is the size of the $$i$$ th group of ties in the predictions (outputs) and $$T$$ ($$S)$$ is the number of such groups in the predictions (outputs) [79].

### Evaluation for binary targets

Evaluating binary outputs is performed using standard protocols in binary classification [80]. We compute the Receiver Operating Characteristic (ROC) curve, which is a 2D plot of the true positive rate $\gamma =P({f}_{\tau }\left(X\right)=1|Y=1)$ as a function of the false positive rate $\eta =P({f}_{\tau }\left(X\right)=1|Y=0)$, where $\tau$ is varied over the entire range of prediction scores. The area under the ROC curve can be mathematically expressed as ${\text{AUC}}={\int }_{0}^{1}\gamma d\eta$ and is the probability that a randomly selected positive example ${x}_{+}$ will be assigned a higher score than a randomly selected negative example ${x}_{-}$ by the model [81]. That is, assuming no ties in prediction scores ${\text{AUC}}=P(s({X}_{+})>s({X}_{-}))$. In the presence of ties, AUC is given by $P(s\left({X}_{+}\right)>s({X}_{-}))+\frac{1}{2}P(s({X}_{+})=s({X}_{-}))$ [82]. The ${\text{AUC}}$ is estimated on the test set $\mathcal{D}$ using the standard numerical computation that allows for ties [83]. Although ${\text{AUC}}$ does not serve as a metric that directly translates into clinical decisions, it is useful in that it shows the degree of separation of the examples from the two groups of data points (positive vs. negative). Another useful property of the ${\text{AUC}}$ is its insensitivity to class imbalance.

Though AUC is a useful measure for capturing the overall performance of a classifier’s score function, it has limitations when applied to a decision-making setting such as the one encountered in the clinic. Typically, clinically relevant score thresholds that determine the variants satisfying Supporting, Moderate or Strong evidence [32] lie in a region of low false positive rate (FPR). A measure well-suited to capture clinical significance of a predictor ought to be sensitive to the variations in the classifier’s performance in the low FPR region (when predicting pathogenicity). However, the contribution of the low FPR region to AUC is relatively small. This is because it not only represents a small fraction of the entire curve, but also because the TPR values in that region are relatively small. Thus, AUC is not sensitive enough to the variation in a predictor’s performance in the low FPR region. To mitigate this problem, we also provide area under the ROC curve truncated to the $[0, 0.2]$ FPR interval. What constitutes low FPR is not well defined; however, it appears that the $[0, 0.2]$ FPR interval combined with the [0, 1] TPR interval is a reasonable choice in CAGI applications; see Figs. 2 and 3. We normalize the truncated AUC to span the entire [0, 1] range by dividing the observed value by 0.2, the maximum possible area below the ROC truncated at FPR = 0.2.

CAGI evaluation of binary classifiers also involves calculation of the Matthews correlation coefficient [84]. The Matthews correlation coefficient (${\text{MCC}}$) was computed as Pearson’s correlation coefficient between binary predictions and target values on the test set $\mathcal{D}$. Efficient MCC estimation was carried out from the confusion matrix [84].

### Evaluation for clinical significance

Current guidelines from the American College for Medical Genetics and Genomics (ACMG) and Association for Molecular Pathology (AMP) established a qualitative framework for combining evidence in support of or against variant pathogenicity for clinical use [32]. These guidelines point to five different levels of pathogenicity and (effectively) nine distinct types of evidence in support of or against variant pathogenicity. The five pathogenicity levels involve classifications into pathogenic, likely pathogenic, variant of uncertain significance (VUS), likely benign, and benign variants, whereas the nine levels of evidential support are grouped into Very Strong, Strong, Moderate, and Supporting for either pathogenicity or benignity, as well as indeterminate evidence that supports neither pathogenicity nor benignity.

Richards et al. [32] have manually categorized different types of evidence and also listed twenty rules for combining evidence for a variant to be classified into one of the five pathogenicity-benignity groups. For example, variants that accumulate one Very Strong and one Strong line of evidence of pathogenicity lead to the classification of the variant as pathogenic; variants that accumulate one Strong and two Supporting lines of evidence lead to the classification of the variant as likely pathogenic, etc. [32] The guidelines allow for the use of computational evidence such that a computational prediction of pathogenicity can be considered as the weakest (Supporting) line of evidence. Thus, combined with other evidence, these methods can presently contribute to a pathogenicity assertion for a variant, but in a restricted and arbitrary way [50]. Since Supporting evidence is qualitatively the smallest unit of contributory evidence in the ACMG/AMP guidelines, we refer to any computational model that reaches the prediction quality equivalent of Supporting evidence and higher as a model that provides contributory evidence in the clinic.

Numerically, a variant that is classified as pathogenic should have at least a 99% probability of being pathogenic given all available evidence, whereas a variant that is likely pathogenic should have at least a 90% probability of being pathogenic given the evidence [32, 52]. Variants that cross the 90% probability threshold for pathogenicity are considered clinically actionable [32]. Analogously, variants with sufficient support for benignity will typically be ruled out from being diagnostic in a clinical laboratory. Note that, though the guidelines provide a probabilistic interpretation of the pathogenicity assertions, they do not provide any general quantitative interpretation of the evidence. Consequently, any framework designed to express the evidence levels quantitatively, must tie such quantitative evidential support to the pathogenicity probabilities, mediated by the ACMG/AMP rules for combining evidence.

The possibility of incorporating computational methods into clinical decision making in a properly calibrated manner presents interesting opportunities and unique challenges. In particular, since the evidence levels are only described qualitatively, it is not obvious how to determine what values of a predictor’s output score qualify as providing a given level of evidence. Thus, to apply a computational line of evidence in the clinic in a principled manner, and consistent with the guidelines, there is a need for a framework that assigns a quantitative interpretation to each evidence level.

Tavtigian et al. [52] proposed such a framework to provide numerical support for each type of evidential strength for its use in ACMG/AMP guidelines for or against variant pathogenicity. This approach is based on the relationship between prior and posterior odds of pathogenicity as well as on independence of all lines of evidential support for a given variant. We briefly review this approach.

Let $E$ be a random variable indicating evidence that can be used in support of or against variant pathogenicity. The positive likelihood ratio (${{\text{LR}}}^{+}$) given concrete evidence $e$ is defined as

6 $$\text{LR}^+=\frac{\mathrm{posterior}\;\mathrm{odds}\;\mathrm{of}\;\mathrm{pathogenicity}}{\mathrm{prior}\;\mathrm{odds}\;\mathrm{of}\;\mathrm{pathogenicity}}$$

or equivalently

7 $${{\text{LR}}}^{+}\left(e\right)=\frac{P\left(Y=1|E=e\right)}{1-P\left(Y=1|E=e\right)}\cdot \frac{1-P\left(Y=1\right)}{P\left(Y=1\right)},$$

where the first term on the right corresponds to the posterior odds of pathogenicity given the evidence and the second term on the right corresponds to the reciprocal of the prior odds of pathogenicity. The prior odds of pathogenicity depend solely on the class prior $P(Y=1)$; that is, the fraction of pathogenic variants in the selected reference set. The expression for ${{\text{LR}}}^{+}$ also allows for an easy interpretation as the increase in odds of pathogenicity given evidence $e$ compared to the situation when no evidence whatsoever is available. The likelihood ratio of 2, for example, states that a variant with evidence $e$ is expected to have twice as large odds of being pathogenic than a variant picked uniformly at random from a reference set. As CAGI only considers computational evidence, we will later replace the posterior probability $P(Y=1|E=e)$ by $P\left(Y=1|f\left(X\right)=1\right)$ for discretized predictors or by $P(Y=1|s\left(X\right)=s)$ for the predictors that output a soft numerical score $s$. The probability $P\left(Y=1|f\left(X\right)=1\right)$ is the positive predictive value (or precision) of a binary classifier, whereas the probability $P(Y=1|s\left(X\right)=s)$ can be seen as the local positive predictive value, defined here in a manner analogous to the local false discovery rate [85].

It can be shown [86] that the positive likelihood ratio can also be stated as

8 $${{\text{LR}}}^{+}\left(e\right)=\frac{P\left(E=e|Y=1\right)}{P\left(E=e|Y=0\right)}$$

thus clarifying that ${{\text{LR}}}^{+}$ can be seen as the ratio of the true positive rate and false positive rate when $P\left(E=e|Y=1\right)$ is replaced by $P\left(f\left(X\right)=1|Y=1\right)$ and $P\left(E=e|Y=0\right)$ by $P\left(f\left(X\right)=1|Y=0\right)$.

Tavtigian et al. [52] give an expression relating the posterior $P(Y=1|E=e)$ to ${{\text{LR}}}^{+}$ and the prior $P(Y=1)$ as

9 $$P\left(Y=1|E=e\right)=\frac{{{\text{LR}}}^{+}\left(e\right)P\left(Y=1\right)}{\left({{\text{LR}}}^{+}\left(e\right)-1\right)P\left(Y=1\right)+1}$$

which itself is obtained from Eq. 7. They also present a framework that allows for assigning probabilistic interpretations to different types of evidential strength (Supporting, Moderate, Strong, and Very Strong) and combining them in a manner consistent with the rules listed in Richards et al. [32] and the probabilistic interpretation of likely pathogenic and pathogenic classes. Their formulation is given in terms of the positive likelihood ratio ${{\text{LR}}}^{+}$ in an exponential form. We restate this model using a notion of the total (or combined) positive likelihood ratio ${{\text{LR}}}_{{\text{T}}}^{+}$, based on all available evidence, ${E}_{{\text{T}}}$, of a variant that is expressed as a product of ${{\text{LR}}}^{+}$ factors from different strengths of evidence as

10 $${{\text{LR}}}_{{\text{T}}}^{+}={{\text{c}}}^{\frac{{n}_{{\text{su}}}}{8}+\frac{{n}_{{\text{mo}}}}{4}+\frac{{n}_{{\text{st}}}}{2}+\frac{{n}_{{\text{vs}}}}{1}},$$

where ${n}_{{\text{su}}}$, ${n}_{{\text{mo}}}$, ${n}_{{\text{st}}}$, and ${n}_{{\text{vs}}}$ are the counts of Supporting (su), Moderate (mo), Strong (st), and Very Strong (vs) lines of evidence present in ${E}_{{\text{T}}}$, and $c$ is the ${{\text{LR}}}^{+}$ value assigned to a single line of Very Strong evidence. It is easy to show that $\sqrt[8]c$, $\sqrt[4]c$, and $\sqrt[2]c$ correspond to the ${{\text{LR}}}^{+}$ for a single line of Supporting, Moderate, and Strong line of evidence, respectively. In other words, the model from Eq. 10 enforces that if a Very Strong piece of evidence increases ${{\text{LR}}}_{{\text{T}}}^{+}$ by a multiplicative factor of $c$, then a Supporting, Moderate, or a Strong piece of evidence increases ${{\text{LR}}}_{{\text{T}}}^{+}$ by a factor of $\sqrt[8]c$, $\sqrt[4]c$, and $\sqrt[2]c$, respectively. For a reasonable consistency with Richards et al. [32] this model also explicitly encodes that one line of Very Strong evidence is equal to the two lines of Strong evidence, four lines of Moderate evidence, and eight lines of Supporting evidence.

The appropriate value of $c$, however, depends on the class prior. It is the smallest number for which the ${{\text{LR}}}_{{\text{T}}}^{+}$ values computed for the qualitative criteria from the likely pathogenic class in Richards et al. [32] reach $P(Y=1|{E}_{{\text{T}}}=e)$ values of at least 0.9 and, similarly, for those in the pathogenic class, reach a $P(Y=1|{E}_{{\text{T}}}=e)$ value of at least 0.99. The dependence on the class prior is due to the conversion between ${{\text{LR}}}_{{\text{T}}}^{+}$ and $P(Y=1|{E}_{{\text{T}}}=e)$ governed by Eq. 9. If the class prior is small, a larger value of ${{\text{LR}}}_{{\text{T}}}^{+}$ will be required to achieve the same posterior level, thereby requiring a larger value of $c$ (Additional file 1: Figure S14).

Tavtigian et al. [52] also proposed that two rules from Richards et al. [32] be revised; that is, one of the rules was proposed to be “demoted” from pathogenic to likely pathogenic, whereas another rule was proposed to be “promoted” from the likely pathogenic to pathogenic. For a class prior of 0.1 that was selected based on the experience from the clinic, the value $c=350$ was found to be suitable. This, in turn, suggests that the Supporting, Moderate, and Strong lines of evidence should require the likelihood ratio values of $\sqrt[8]c=2.08$, $\sqrt[4]c=4.32$, and $\sqrt[2]c=18.7$, respectively. However, note again that for different priors, these values will be different; see next section and Additional file 1: Figure S14. Moreover, while the level of posterior for the combined evidence (Eq. 13) is required to be at least 0.9 to satisfy the likely pathogenic rule and 0.99 for pathogenic, this does not mean that the posterior level for a single line of evidence is the same for all values of $c$. This is a consequence of the fact that the framework provides intuitive interpretation only at the level of the combined posterior.

When drawing evidence from a pathogenicity predictor, it is necessary to further clarify what evidence is in the first place. At least two options are available: (i) the evidence is the score $s(x)$; that is, a raw prediction of pathogenicity, or (ii) the evidence is a discretized prediction ${f}_{\tau }\left(x\right)$, obtained by thresholding $s(x)$. These approaches, referred to here as local and global, respectively, lead to different interpretations because all evaluation metrics hold only on average, either over all variants with a score $s(x)$ or all variants satisfying ${f}_{\tau }\left(x\right)=1$; i.e., having a score above $\tau$. When both $s(x)$ and ${f}_{\tau }(x)$ are available, this leads to difficulties in interpreting the results of the global approach because all scores $s(x)$ that map into ${f}_{\tau }\left(x\right)=1$ will be treated identically. Unfortunately, this implies that scores $s$ greater than but still close to $\tau$ most likely do not meet the levels of evidential strength for the interval. At the same time, scores close to the high end of the range almost certainly make the levels of evidential strength above the designated level. This means that a clinician seeing a variant with score slightly above $\tau$ would have to interpret this prediction as contributory to pathogenicity, yet this interpretation would almost certainly be incorrect. Based on the recommendations from the ClinGen’s Sequence Variant Interpretation group, [50] we focus on the local view as well as local performance criteria to define levels of evidential strength and assess whether methods achieve these levels. In the end, however, we also provide global estimates to understand the performance of each tool more comprehensively.

We define the local positive likelihood ratio as

11 $${{\text{lr}}}^{+}\left(s\right)=\frac{p\left(s|Y=1\right)}{p\left(s|Y=0\right)},$$

where $p\left(s|Y=y\right)$, for $y\in \{0, 1\}$ are class-conditional densities.

We obtain an estimate of the local positive likelihood ratio ${\widehat{{\text{lr}}}}^{+}$ from the test data as described in the section titled “Computing clinically relevant measures.” Now, the threshold to determine the variants with Supporting level of evidence is given as the minimum score above which all variants achieve local positive likelihood ratio value greater than or equal to $\sqrt[8]c$; i.e.,

12 $$\tau_\text{su}=\text{min}\left\{\tau:\forall s\geq\tau,\widehat{\text{lr}}^+\left(s\right)\geq\sqrt[8]c\right\}$$

though we note that Pejaver et al. [50] incorporated an additional factor based on the confidence interval for ${\widehat{{\text{lr}}}}^{+}\left(s\right)$ to result in more stringent recommendations for score thresholding. Similarly, the thresholds for variants with Moderate, Strong, and Very Strong evidence are given by $\tau_\text{mo}=\text{min}\left\{\tau:\forall s\geq\tau,\widehat{\text{lr}}^+\left(s\right)\geq\sqrt[4]c\right\}$, $\tau_\text{st}=\text{min}\left\{\tau:\forall s\geq\tau,\widehat{\text{lr}}^+\left(s\right)\geq\sqrt[2]c\right\}$ and ${\tau }_{{\text{vs}}}={\text{min}}\left\{\tau : \forall {\text{s}}\ge \tau , {\widehat{{\text{lr}}}}^{+}\left(s\right)\ge {\text{c}}\right\}$.

Once the threshold set $\{{\tau }_{{\text{su}}},{\tau }_{{\text{mo}}},{\tau }_{{\text{st}}},{\tau }_{{\text{vs}}}\}$ is determined, we can compute either the global ${{\text{LR}}}^{+}$ (e.g., $s\ge {\tau }_{{\text{su}}}$) or the ${{\text{LR}}}^{+}$ corresponding to an interval of scores (e.g., ${\tau }_{{\text{su}}}\le s<{\tau }_{{\text{mo}}}$) by computing the true positive rate and false positive rate for a given set of scores. A global positive predictive value can be similarly estimated once the class prior is known.

In all CAGI evaluations, a predictor is considered to provide contributory evidence in a clinical setting if it reaches any one of the evidence levels according to the ACMG/AMP guidelines, and according to the model by Tavtigian et al. [52] and recommendations by Pejaver et al. [50]. Among predictors that reach the desired levels of evidential support, the ones that reach higher levels are generally considered favorably. However, we have not considered any criterion to rank the predictors that reach the same levels of evidential support.

### Selection of class priors for variant pathogenicity

Different clinical scenarios require the use of different class priors of variant pathogenicity. We generally distinguish between two clinical situations.

In the first setting, a clinician is presented with a proband with specific phenotypic expression and the objective is to find variants responsible for the clinical phenotype. In certain monogenic disorders with Mendelian inheritance patterns, the fraction of rare variants found to be pathogenic can be as high as 25%, as in the case of the NAGLU challenge. Similarly, Tavtigian et al. [52] report an experience-based prior of 10% based on their work with BRCA genes, which we adopted in this work.

The second setting reflects situations such as screening for potential secondary variants. Here we have used an estimate by Pejaver et al. [40] that up to 1.5% of missense variants in an apparently healthy individual could be disease-causing.

Overall, prior probability of pathogenicity was set to 1 and 10% to demonstrate the distinction in the level of evidential support necessary. These resulted in $c=8511$ and $c=351$, respectively (note that $c=351$ was selected instead of $c=350$ to avoid rounding errors in finding a $c$ that best models ACMG/AMP rules). In each functional missense challenge, the level of prior probability observed for each gene based on experimental data was further considered. For large class priors such as 50% or above, the Tavtigian et al. [52] framework holds only when an additional rule from Richards et al. [32] is removed; that is, we ignored that two Supporting lines of evidence for benignity assert a likely benign variant.

### Performance measures for clinical application

#### Diagnostic odds ratio

The diagnostic odds ratio (${\text{DOR}}$) is commonly used in biomedical sciences to measure the increase in odds of pathogenicity in the presence of evidence $e$ compared to the odds of pathogenicity in the absence of $e$; [86] that is,

13 $${\text{DOR}}\left(e\right)=\frac{P\left(Y=1|E=e\right)}{1-P\left(Y=1|E=e\right)}\cdot \frac{1-P\left(Y=1|E\ne e\right)}{P\left(Y=1|E\ne e\right)}.$$

The difference between Eq. 7 and Eq. 13 is that the prior odds, those governed by the prior $P\left(Y=1\right)$ and used in Eq. 7, are replaced by the odds governed by the probability $P\left(Y=1|E\ne e\right)$; that is, odds of pathogenicity when the evidence $e$ was not the one that was observed. The quantity $P\left(Y=0|E\ne e\right)=1-P\left(Y=1|E\ne e\right)$ is referred to as the negative predictive value when the observed evidence is $f\left(X\right)=0$. ${\text{DOR}}\in [0,\infty )$ can also be expressed as

14 $${\text{DOR}}\left(e\right)=\frac{{{\text{LR}}}^{+}\left(e\right)}{{{\text{LR}}}^{-}\left(e\right)},$$

where ${{\text{LR}}}^{+}(e)$ is defined in Eq. 8 and

15 $${{\text{LR}}}^{-}(e)=\frac{P\left(E\ne e|Y=1\right)}{P\left(E\ne e|Y=0\right)}.$$

In contrast to typical studies of variant risk assessment [87] and polygenic risk scores [64], ${\text{DOR}}$ was calculated without adjustments for usual confounders such as race and ethnicity that are generally not available in CAGI challenges and, technically, produce conditional odds ratios [86]. However, the ${\text{DOR}}$ values estimated in our experiments have an identical interpretation as the results of logistic regression run with a single independent variable (co-variate) at a time. Glas et al. [86] give a broader coverage of diagnostic odds ratios that further connect some of the quantities discussed here (e.g., ${\text{AUC}}$ vs. ${\text{DOR}}$).

We only consider DOR with the computational evidence of the “global” type; that is, when $s\left(x\right)\ge \tau$. Consequently, DOR at $\tau$ can be expressed as

16 $${\text{DOR}}\left(\tau \right)=\frac{{{\text{LR}}}^{+}\left(\tau \right)}{{{\text{LR}}}^{-}\left(\tau \right)}=\frac{P\left(s\left(X\right)\ge \tau |Y=1\right)}{P\left(s\left(X\right)\ge \tau |Y=0\right)}\frac{P\left(s\left(X\right)<\tau |Y=0\right)}{P\left(s\left(X\right)<\tau |Y=1\right)}.$$

Unlike positive likelihood ratio, DOR does not have a “local” version. This is because one cannot define a local negative likelihood ratio.

#### Percent of variants predicted as pathogenic

In addition to finding whether a method reaches Supporting, Moderate, or Strong levels of evidence, it is important to also quantify the proportion of variants in the reference set for which a given evidence level is reached. To this end, for a given score threshold $\tau$, we define the percent of variants in the reference set that the method assigns a score as high as or higher than $\tau$, and refer to it as “probability of pathogenic (positive) predictions,” or PPP. Mathematically, it can be expressed as the following probability

17 $${\text{PPP}}\left(\tau \right)=P\left(s\left(X\right)\ge \tau \right).$$

The probability (equivalently, percent) of variants reaching a given level of evidence can now be quantified as ${\text{PPP}}\left(\tau \right)$, where $\tau$ is the score threshold at which a variant is declared to meet the desired evidential support.

#### Posterior probability of pathogenicity

Given a method, the posterior probability of pathogenicity or the absolute risk for a variant is defined as the probability that the variant is pathogenic based on the score it is assigned by the method. It is expressed as

18 $$\rho \left(s\right)=P\left(Y=1|s\left(X\right)=s\right).$$

We also refer to this quantity as a local positive predictive value or local precision.

#### Relative risk

Given a method, the relative risk (RR) of pathogenicity of a variant is defined as the posterior probability of pathogenicity (based on the score assigned by the method) relative to the prior probability of pathogenicity. It is expressed as the following ratio

19 $${\text{RR}}\left(s\right)=\frac{P\left(Y=1|s\left(X\right)=s\right)}{P\left(Y=1\right)}.$$

The prior probability of pathogenicity can also be interpreted as the average of the posterior probability over all variants in the reference set; that is

20 $$\begin{aligned}{\mathbb{E}}\left[P\left(Y=1|s\left(x\right)=s\right)\right] &= {\int }_{\mathcal{X}}P\left(Y=1|s\left(x\right)=s\right)p\left(x\right)dx \\ &= {\int }_{\mathbb{R}}P\left(Y=1|s\right)p\left(s\right) ds\\ &= {\int }_{\mathbb{R}}p\left(s|Y=1\right)P\left(Y=1\right) ds\\ &= P\left(Y=1\right),\end{aligned}$$

where the last step follows since $p\left(s|Y=1\right)$ is a density function and its integral over ${\mathbb{R}}$ is 1. Observe that our definition of relative risk is an extension of the “global” version used in clinical applications where the denominator would be $P\left(Y=1|s\left(X\right)\ne s\right)$, which effectively equals $P\left(Y=1\right)$ for all predictors outputting continuous scores.

#### Computing clinically relevant measures

We show here how the measures for evaluation of binary targets and clinically relevant measures are computed from the test data $\mathcal{D}$. It is necessary to be cautious when making decisions on a reference (target) population based on the measures computed on the test set $\mathcal{D}$. Some of the measures computed on $\mathcal{D}$ accurately represent the corresponding values on the target population. However, other measures are biased because the test data set for many challenges is not representative of the target population. In particular, the proportions of positives (e.g., pathogenic variants) in the test set ${\alpha }_{\mathcal{D}}={P}_{\mathcal{D}}(Y=1)$ may be vastly different from that in the target population $\alpha =P\left(Y=1\right)$. Consequently, the class-prior dependent measures, when estimated directly from the test set, are incorrectly calibrated to the test set class priors.

Fortunately, the class-prior dependent measures can be corrected using an estimate of the target population’s class priors if known or if estimated using a principled approach [88, 89]. The correction is derived under the assumption that the reference population and the test set are distributionally identical, except for the differences in class priors. To elaborate, the target distribution of inputs $p\left(x\right)$ can be expressed in terms of the class-conditional distributions, $p\left(x|Y=y\right)$ for $y\in \left\{0, 1\right\}$, and the class priors as follows

21 $$p\left(x\right)=\alpha \cdot p\left(x|Y=1\right)+\left(1-\alpha \right)\cdot p\left(x|Y=0\right).$$

We assume that the test set distribution of inputs might have different class priors, but the same class-conditional distributions as the target population. Precisely,

22 $$\begin{aligned}{p}_{\mathcal{D}}\left(x\right) &= {\alpha }_{\mathcal{D}}{p}_{\mathcal{D}}\left(x|Y=1\right)+\left(1-{\alpha }_{\mathcal{D}}\right){p}_{\mathcal{D}}\left(x|Y=0\right)\\ &= {\alpha }_{\mathcal{D}}p\left(x|Y=1\right)+\left(1-{\alpha }_{\mathcal{D}}\right)p\left(x|Y=0\right).\end{aligned}$$

It is easy to see that any of the clinical and non-clinical measures that only depend on the class-conditional distributions, but not class priors, when computed on the test set is an unbiased estimate of the measure on the target population. However, if a measure also depends on the class priors, it needs to be corrected to reflect the reference population’s class prior. All the class-prior independent measures used in this paper can be expressed in terms of class-conditional derived quantities such as the true positive rate (TPR), the false positive rate (FPR), and the local positive likelihood ratio ${{\text{lr}}}^{+}\left(s\right)$. The class-prior dependent measures additionally have the class-prior in their expressions.

#### ***TPR, FPR, and ***${{\varvec{l}}{\varvec{r}}}^{+}\left({\varvec{s}}\right)$

Formally, TPR is defined as the proportion of positive inputs that are correctly predicted to be positive. Mathematically,

23 $${\text{TPR}}\left(\tau \right)=P\left(s\left(x\right)\ge \tau |Y=1\right),$$

where $s\left(x\right)$ is a continuous score function of a classifier and $\tau$ is a threshold such that an input scoring above $\tau$ is predicted to be positive. Similarly, FPR is defined as the proportion of negative inputs that are incorrectly predicted to be positive. Mathematically,

24 $${\text{FPR}}\left(\tau \right)=P\left(s\left(x\right)\ge \tau |Y=0\right).$$

TPR and FPR can be computed from the test data as the proportion of positive and negative test inputs scoring $\ge \tau$, respectively. That is,

25 $$\begin{aligned}\widehat{{\text{TPR}}}\left(\tau \right)&= \frac{{\sum }_{x\in {\mathcal{D}}_{+}}I\left[s\left(x\right)\ge \tau \right]}{|{\mathcal{D}}_{+}|}\\ \widehat{{\text{FPR}}}\left(\tau \right)&= \frac{{\sum }_{x\in {\mathcal{D}}_{-}}I\left[s\left(x\right)\ge \tau \right]}{|{\mathcal{D}}_{-}|},\end{aligned}$$

where ${\mathcal{D}}_{+}$ and ${\mathcal{D}}_{-}$ are the subsets of points in the test set $\mathcal{D}$ labeled as positive and negative, respectively.

Some of the clinically relevant measures used in our study are “local” in nature in the sense that they are derived from a local neighborhood around a score value instead of the entire range of scores above (or below) the threshold. Such measures can be expressed in terms of the local positive likelihood ratio ${{\text{lr}}}^{+}\left(s\right)$. To compute ${{\text{lr}}}^{+}\left(s\right)$, we exploit its relationship to the posterior probability at score $s$; that is,

26 $$\begin{aligned}P\left(Y=1|s\left(X\right)=s\right) &= \frac{p\left(s\left(X\right)=s|Y=1\right)P\left(Y=1\right)}{p\left(s\left(X\right)=s\right)}\\ &= \frac{p\left(s\left(X\right)=s|Y=1\right)P\left(Y=1\right)}{p\left(s\left(X\right)=s|Y=1\right)P\left(Y=1\right)+p\left(s\left(X\right)=s|Y=0\right)P\left(Y=0\right)}\\ &= \frac{{{\text{lr}}}^{+}\left(s\right)P\left(Y=1\right)}{{{\text{lr}}}^{+}\left(s\right)P\left(Y=1\right)+P\left(Y=0\right)}\\ &= \frac{{{\text{lr}}}^{+}\left(s\right)P\left(Y=1\right)}{\left({{\text{lr}}}^{+}\left(s\right)-1\right)P\left(Y=1\right)+1}.\end{aligned}$$

Similarly, the test data posterior probability can be expressed as

27 $${P}_{\mathcal{D}}\left(Y=1|s\left(X\right)=s\right)=\frac{{{\text{lr}}}^{+}\left(s\right){P}_{\mathcal{D}}\left(Y=1\right)}{\left({{\text{lr}}}^{+}\left(s\right)-1\right){P}_{\mathcal{D}}\left(Y=1\right)+1}.$$

Note that since ${{\text{lr}}}^{+}\left(s\right)$ only depends on the class-conditional distribution, it does not change when defined on the target population. Unlike the target population’s posterior, the test data posterior can be estimated from the test data as described below. Once the test posterior is estimated, the equation above can be inverted to estimate ${{\text{lr}}}^{+}$ as

28 $${\widehat{{\text{lr}}}}^{+}\left(s\right)=\frac{{\widehat{P}}_{\mathcal{D}}\left(Y=1|s\left(X\right)=s\right)}{1-{\widehat{P}}_{\mathcal{D}}\left(Y=1|s\left(X\right)=s\right)}\cdot \frac{{1-P}_{\mathcal{D}}\left(Y=1\right)}{{P}_{\mathcal{D}}\left(Y=1\right)},$$

where the ${\widehat{P}}_{\mathcal{D}}\left(Y=1|s\left(X\right)=s\right)$ is an estimate of the test data posterior and ${P}_{\mathcal{D}}\left(Y=1\right)$ is the proportion of positives in the test data, which may differ from the true prior for a randomly picked variant in the gene of interest or another reference sample. Note that though the formula above expresses ${\widehat{{\text{lr}}}}^{+}\left(s\right)$ in terms of the prior odds, suggesting a dependence on the class prior, ${\widehat{{\text{lr}}}}^{+}\left(s\right)$ is class-prior independent, as discussed earlier. In theory, ${P}_{\mathcal{D}}\left(Y=1|s\left(X\right)=s\right)$ is the proportion of pathogenic variants among all variants in $\mathcal{D}$ having a score $s$. Therefore, estimating the local posterior efficiently would require observing the same score many times in the set of variants with known labels. This is unlikely since we only have scores for a finite set of variants and thus the posterior cannot be estimated without making further assumptions. However, assuming that the posterior is a smooth function of the score—similar scores correspond to similar local posterior values—we estimate the posterior as the proportion of pathogenic variants in a small window around the score; that is, $\left[s-\epsilon ,s+\epsilon \right]$, where $\epsilon$ was selected to be 5% of the range of the predictor’s outputs, with the range considered to be an interval between the 5th and 95th percentile of predicted values on the dataset, selected as such to minimize the influence of outliers. In addition, for stable estimates, we required that at least 10% of the variants, up to a maximum of 50 variants, from the data set are within a window; therefore, the final window size was dependent on score $s$ and data set $\mathcal{D}$.

#### Measures that do not require correction

Among the measures considered in this paper, TPR, FPR, ROC curve, AUC, ${{\text{LR}}}^{+}$, ${{\text{LR}}}^{-}$, DOR, and ${{\text{lr}}}^{+}$ do not require correction. Class-prior independence of TPR, FPR, and lr^+^ is obvious from their definitions as discussed earlier. ROC curve is obtained by plotting TPR against FPR and consequently, it is also class-prior independent. By extension AUC, being the area under the ROC curve, is also class-prior independent. The global positive likelihood ratio ${{\text{LR}}}^{+}$, formulated with the evidence of the type $s\left(x\right)\ge \tau$, is given by ${\text{TPR}}(\uptau)/{\text{FPR}}(\uptau)$. Similarly, the global ${{\text{LR}}}^{-}$ is given by $(1-{\text{TPR}}(\uptau ))/(1-{\text{FPR}}(\uptau ))$. Since DOR is the ratio of ${{\text{LR}}}^{+}$ and ${{\text{LR}}}^{-}$, it is by extension class-prior independent.

#### Measures that require correction

Among the measures considered in this paper, probability of pathogenic predictions (PPP), positive predictive value (PPV), posterior probability ($\rho$), and relative risk (${\text{RR}}$), being class-prior dependent, require corrections to be properly applied to the target population. To show that the measures are indeed class-prior dependent, we re-formulate them by separating the class-prior from the class-conditional dependent terms.

29 $$\begin{aligned}{\text{PPP}}\left(\tau \right)&= P\left(s\left(X\right)\ge \tau \right)\\ &= P\left(s\left(X\right)\ge \tau |Y=1\right)P\left(Y=1\right)+P\left(s\left(X\right)\ge \tau |Y=0\right)P\left(Y=0\right)\\ &= \alpha {\text{TPR}}\left(\tau \right)+\left(1-\alpha \right){\text{FPR}}\left(\tau \right)\end{aligned}$$

30 $$\begin{aligned}{\text{PPV}}\left(\uptau \right)&= P\left(Y=1|S\left(X\right)\ge \tau \right)\\ &= \frac{P\left(s\left(X\right)\ge \tau |Y=1\right)P\left(Y=1\right)}{P\left(s\left(X\right)\ge \tau \right)}\\ &= \frac{\alpha {\text{TPR}}\left(\tau \right)}{\alpha {\text{TPR}}\left(\tau \right)+\left(1-\alpha \right){\text{FPR}}\left(\tau \right)}\end{aligned}$$

31 $$\begin{aligned}\rho \left(s\right)&= P\left(Y=1|s\left(X\right)=s\right)\\ &= \frac{{\mathrm{\alpha lr}}^{+}\left(s\right)}{\alpha \left({{\text{lr}}}^{+}\left(s\right)-1\right)+1},\end{aligned}$$

where the derivation is the same as that for Eq. 26.

32 $$\begin{aligned}{\text{RR}}\left(s\right)&= \frac{P\left(Y=1|s\left(X\right)=s\right)}{\alpha }\\ &= \frac{{{\text{lr}}}^{+}\left(s\right)}{\alpha \left({{\text{lr}}}^{+}\left(s\right)-1\right)+1}.\end{aligned}$$

We use the expressions above to correctly calculate class-prior dependent metrics on the target domain by first computing the class-conditional dependent terms (TPR, FPR, or ${{\text{lr}}}^{+}$) using the test data $\mathcal{D}$ and then using an estimate of the class prior of the target distribution in the corresponding expression.

### Statistical significance and confidence interval estimation

All *p*-values and confidence intervals in CAGI evaluations were estimated using bootstrapping with 1000 iterations [90].

### Supplementary Information

**Additional file 1.** Description of the analysis framework, implementation details, analyzed and non-analyzed CAGI challenges and the corresponding data. It also contains all supplementary figures and a description of the supplementary tables [92–166].

**Additional file 2: Table S2.** Analysis of all biochemical effect challenges.

**Additional file 3: Table S3.** Meta-analysis of all biochemical effect challenges.

**Additional file 4: Table S4.** Analysis of the Annotate All Missense challenge.

**Additional file 5: Table S5.** Analysis of cancer challenges.

**Additional file 6: Table S6.** Analysis of splicing and transcription challenges.

**Additional file 7: Table S7.** Analysis of the complex trait challenge: Crohn’s disease (CAGI4).

**Additional file 8.** Review history.

## Data Availability

The code used for the analyses in this paper is available on GitHub (https://github.com/genomeinterpretation/CAGI50) and zenodo (doi.org/10.5281/zenodo.8436229)  [91] under an MIT Open Source license. **Data availability**: The answer keys for all reanalyzed CAGI challenges are available on the CAGI website. Access requires registration including acceptance of a data use agreement. The CAGI website is an archival venue that has been in operation longer than resources such as Zenodo. Papers containing publicly available answer keys are also referenced in the table.

**Table:** 

Challenges	CAGI Year	Data source type	DOI
NAGLU	CAGI 4	Publication	10.1371/journal.pone.0200008
PTEN/TPMT	CAGI 5	Publication	10.1038/s41588-018-0122-z
Annotate All Missense	CAGI 5	CAGI website^*,**^	
CALM1	CAGI 5	Publication	10.15252/msb.20177908
GAA	CAGI 5	CAGI website	
CBS	CAGI 1 & 2	CAGI website	
SUMO-ligase	CAGI 4	CAGI website	
PCM1	CAGI 5	Publication	10.1038/s41467-020-19637-5
L-PYK	CAGI 4	CAGI website	
p53 rescue	CAGI 2	Data file (training set) CAGI website (answer key)	10.24432/C5T89H
Frataxin	CAGI 5	Publication	10.1002/humu.23843
p16	CAGI 3	Publication	10.1002/humu.22550
ENIGMA	CAGI 5	CAGI website	
BRCA	CAGI 3	CAGI website	
Vex-Seq	CAGI 5	CAGI website	
eQTL	CAGI 4	Publication	10.1002/humu.23197
MaPSy	CAGI 5	Publication	10.1186/s13059-019-1653-z
Regulation-Satutation	CAGI 5	CAGI website	
Crohn’s	CAGI 4	Publication^***^ CAGI website (answer key)	10.1097/MIB.0000000000001235 (pediatric IBD cohort). 10.1016/j.ebiom.2016.08.037 (healthy controls) https://portal.popgen.de
SickKids	CAGI 4 & 5	Publication^***^	10.1002/humu.23874

^*^ The CAGI website URL is https://genomeinterpretation.org

^**^ A subset of variants used in the Annotate All Missense challenge were obtained from the a proprietary version of the HGMD, database. These variants (those in HGMD 2020.4 but not HGMD 2019) are excluded from the answer key on the CAGI website but can be obtained under the HGMD Professional license (https://www.hgmd.cf.ac.uk).

^***^ The full patient data for the Crohn’s and SickKids challenges are not maintained on the CAGI website because of patient privacy issues. However, approved users may obtain these from the authors of the corresponding publications in the above table. Data file (training set) CAGI website (answer key) Publication^***^ CAGI website (answer key) 10.1097/MIB.0000000000001235 (pediatric IBD cohort). 10.1016/j.ebiom.2016.08.037 (healthy controls) https://portal.popgen.de ^*^ The CAGI website URL is https://genomeinterpretation.org ^**^ A subset of variants used in the Annotate All Missense challenge were obtained from the a proprietary version of the HGMD, database. These variants (those in HGMD 2020.4 but not HGMD 2019) are excluded from the answer key on the CAGI website but can be obtained under the HGMD Professional license (https://www.hgmd.cf.ac.uk). ^***^ The full patient data for the Crohn’s and SickKids challenges are not maintained on the CAGI website because of patient privacy issues. However, approved users may obtain these from the authors of the corresponding publications in the above table.
