# Supplementary material for: CAGI, the Critical Assessment of Genome Interpretation, establishes progress and prospects for computational genetic variant interpretation methods
Source: Genome Biol. 2024 Feb 22;25:53. doi: 10.1186/s13059-023-03113-6 (PMC10882881; doi:10.1186/s13059-023-03113-6)
Supplement: Supplementary file 1 — Additional file 1. Description of the analysis framework, implementation details, analyzed and non-analyzed CAGI challenges and the corresponding data. It also contains all supplementary figures and a description of the supplementary tables [92–166]. [file 13059_2023_3113_MOESM1_ESM.docx]

**Supplementary Information**

**Table of contents**

[1. Unified Analysis Framework 2](#_Toc148184864)

[2. Analyzed challenges 5](#_Toc148184865)

[2.1. Coding Challenges 5](#_Toc148184866)

[NAGLU 5](#_Toc148184867)

[PTEN and TPMT 5](#_Toc148184868)

[GAA 7](#_Toc148184869)

[CBS 7](#_Toc148184870)

[SUMO ligase 8](#_Toc148184871)

[CALM1 9](#_Toc148184872)

[Frataxin 10](#_Toc148184873)

[PCM1 10](#_Toc148184874)

[L-PYK 11](#_Toc148184875)

[p16 12](#_Toc148184876)

[p53 rescue 13](#_Toc148184877)

[BRCA 13](#_Toc148184878)

[ENIGMA 14](#_Toc148184879)

[Annotate All Missense 15](#_Toc148184880)

[2.2. Expression and Splicing Challenges 16](#_Toc148184881)

[Vex-seq 16](#_Toc148184882)

[MaPSy 17](#_Toc148184883)

[eQTL 18](#_Toc148184884)

[Regulation-Saturation 19](#_Toc148184885)

[2.3. Complex disease challenges 20](#_Toc148184886)

[Crohn’s disease 20](#_Toc148184887)

[3. Non-analyzed challenges 22](#_Toc148184888)

[4. Implementation Details 31](#_Toc148184889)

[Experimental-Max 31](#_Toc148184890)

[ROC from discrete class labels 31](#_Toc148184891)

[log-log AUC 31](#_Toc148184892)

[Bootstrap 32](#_Toc148184893)

[4.1. Handling infinity and indeterminate values 33](#_Toc148184894)

[4.2. Handling ties in scores 33](#_Toc148184895)

[5. Supplementary Figures 34](#_Toc148184896)

[6. Supplementary Tables 59](#_Toc148184897)

**List of figures**

[Figure S1: TPMT, CALM1 and GAA](#Fig2)

[Figure S2A: CBS CAGI 1](#Fig3A)

[Figure S2B: CBS CAGI 2](#Fig3B)

[Figure S3: SUMO Ligase](#Fig4)

[Figure S4: PCM1 and L-PYK](#Fig5)

[Figure S5: p53 Rescue](#Fig6)

[Figure S6: Frataxin and p16](#Fig7)

[Figure S7: log-log ROC for NAGLU, PTEN and Annotate all Missense](#Fig1)

[Figure S8: prediction-experimental value correlation and prediction-prediction correlation comparison](#Fig8)

[Figure S9: Annotate all Missense bi-class genes](#Fig14)

[Figure S10A: ENIGMA](#Fig9A)

[Figure S10B: BRCA](#Fig9B)

[*Figure S11: Vex-seq*](#Fig10)

[*Figure S12A: eQTL Regulatory hit*](#Fig11A)

[*Figure S12B: eQTL log2 allelic skew and emVar*](#Fig11B)

[*Figure S13: Examples of structure-based explanations of variant impact.*](#Fig12)

[*Figure S14: The local* $lr^{+}$ *cuttoffs versus prior for different clinical evidence levels*](#Fig13)

# Unified Analysis Framework

All selected CAGI challenges were analyzed using a unified framework. The analysis includes all the biochemical effect challenges considered to be of high-quality, the Annotate All Missense challenge, the high-throughput splicing challenges, the eQTL challenges, and the third Crohn’s challenge (see main text figure 1 for further detail). Here we summarize the data processing pipelines and evaluation approaches. We begin with a high-level description of the commonalities and differences between different datasets and their evaluation.

The datasets were evaluated on regression and/or binary classification type tasks. The goal of the regression type task is to measure the performance of a method on predicting a continuous measurement (functional activity, growth, etc.) made in the experiment that directly or indirectly measures a quantity of interest. For the purpose of the description that follows we will refer such continuous measurements as *experimental values*. Scatter plots and measures of R-squared, RMSE, Pearson’s correlation, Spearman’s correlation and Kendall’s $\tau$ were used for evaluating the prediction of experimental values; see Methods. In many challenges the data was generated with experimental replicates that allowed recording the standard deviation of the experimental values. When available, the standard deviation was used to generate the predictions of a positive control that gives an upper limit on the method performance given the experimental variability. We refer to the positive control as the *Experimental-Max* (sec. Implementation Details).

In many challenges, the experimental values were further used to define classes for the classification task. This was based on either thresholding the experimental values using reasonable thresholds or a combination of threshold, confidence or statistical significance used by previous assessors. Although some of the challenges originally defined more than one class from the experimental values, in such cases we either removed or merged one or more classes to create a binary classification dataset. By convention, the numeric values 1 and 0 are used to represent the positive and negative class, respectively. For some non-coding challenges, we created two binary classification datasets; e.g., one where over-splicing is used as the positive class and the other where under-splicing is used as the positive class. ROC curve, AUC and local $lr^{+}$ curve were used for evaluating classification tasks. Additionally, for many challenges we also provide a clinically relevant classification analysis with Truncated AUC, log-log AUC (see Implementation Details), along with the corresponding ROC curves, local posterior probability of pathogenicity ($\rho)$ curve and measurements PPP, TPR, FPR, $LR^{+}$ (global), $LR^{-},$DOR, MCC and PPV at clinically relevant evidence (Supporting, Moderate and Strong) thresholds; see Methods. Additionally, for complex traits dataset on Crohn’s disease, we also provide a relative risk (RR) curve.

In many challenges (mostly functional challenges), the predictions for the experimental values also serve as the continuous method score for binary classification. In some of these cases, where a low experimental value corresponds to the positive class, the predictions are negated (multiplied by −1) to create the method score. This ensures that a high method score corresponds to the positive class. This transformation is necessary to correctly compute the classification measures. However, note that when the results are presented in figures (posterior probability and $lr^{+}$ curves) the unnegated values are used for consistency with the scatter plots. As a side-effect of this presentation, in some figures the evidence thresholds represent a region towards its left, whereas in other cases it represents a region towards its right depending on whether the predicted experimental values required negation or not, respectively. As a rule of thumb, for a method that captures some signal for the classification task, the evidence thresholds represent the region towards the direction of increasing $lr^{+}$ or posterior curve. An Experimental-Max baseline for the classification measures is also reported if the standard deviation of the experimental values is available.

In some challenges (most expression and splicing challenges) the participants were asked to submit a method score for the classification task in addition to the prediction for the experimental values. In such cases the method score for the classification is used for the classification related analysis and experimental value predictions are used in the regression related analysis.

The CAGI cancer challenges and the Annotate All Missense dataset analyzed in this paper use curated ground truth variants; that is, they do not fall in the framework of running experimental assays and, consequently, do not have an experimental value to be predicted. Only classification-related analysis is provided for these datasets.

Some biochemical effect challenges, in spite of measuring an experimental value, only required participants to submit discrete labels for the classification task. Here we only report the classification related analysis. The predicted binary class labels are converted to a numeric method score: 1 for a positive prediction and 0 for a negative prediction. Since the resultant method score is binary and discrete, local $lr^{+}$and posterior probability of pathogenicity are only defined at the two values taken by the method score, instead of being a continuous curve. Furthermore, the local $lr^{+}$ and the global$LR^{+}$ coincide in this case.

# Analyzed challenges

## Coding Challenges

### NAGLU

The NAGLU dataset contains missense variants responsible for the production of N-acetyl-glucosaminidase (NAGLU, NP_000254.2). Deficiency of NAGLU is indicated in neurodegenerative diseases Mucopolysaccharidosis IIIB (MPS IIIB) or Sanfilippo B disease (OMIM #252920). BioMarin functionally assessed the enzymatic activity of 165 novel missense mutations in the ExAC dataset by transfecting plasmids containing cDNAs encoding each of the mutant proteins into HEK293 cells. The activity levels were normalized to represent the fraction of wildtype NAGLU activity.^49^ A value of 0 represents no activity, 1 represents wildtype-level of activity and >1 represents an activity greater than the wildtype activity. For example, 0.7 means 70% of wildtype activity and 1.3 means 130% of wildtype activity. Each mutant was assayed in at least three independent transfection experiments. The results from these three determinations were averaged to give the mean activity and the standard deviation was also calculated.

In the NAGLU challenge of CAGI4, participants were asked to submit predictions for the fractional enzymatic activity of the variants. A total of 10 teams participated in the NAGLU challenge, submitting predictions from 17 different models.

We first compared the predictions to experimental fractional activity in a regression type analysis. For a classification type analysis, each variant was assigned either “pathogenic” or “benign” label based on its experimental fractional activity. Variants having activity value below 0.15 were deemed “pathogenic” and the remaining variants were considered to be “benign”. The threshold is consistent with that observed from previously identified pathogenic mutations as described by Clark et al,^49^ and also used in the CAGI5 assessment paper for NAGLU.^22^ Out of the 165 variants, the experimental values could not be measured for 2 variants. Out of the remaining 163 variants, 40 were assigned pathogenic label, giving an overall 25% of pathogenic variants (data prior). As stated in the Main file, this appears to be a good estimate of the prior probability of pathogenicity in a diagnostic setting. For the clinical analysis, we used the data prior to compute the clinically relevant thresholds and all class-prior dependent measures. The results of the clinical analysis with the general diagnostic (0.1) and screening (0.01) priors are also reported. The predicted fractional activity was also used as the method score for classification and clinical analysis. Our evaluation for the NAGLU challenge is summarized in Main file: Figure 2 and Figure S7 and Additional file 2: Table S2.

### PTEN and TPMT

The gene PTEN (Phosphatase and TEnsin Homolog) encodes for a protein that is an important secondary messenger molecule promoting cell growth and survival. Thiopurine S-methyl transferase (TPMT) is a key enzyme involved in the metabolism of thiopurine drugs. A library of 4002 PTEN and 3952 TPMT mutations was assessed to measure the stability of the variant protein using a multiplexed Variant Stability Profiling (VSP) assay. The VSP assay exploits a fluorescent reporter system to measure steady-state abundance of missense protein variants. Here, each cell expresses a protein variant fused to EGFP. The stability of the variant protein dictates the abundance of the fusion protein and thus the EGFP level of the cell. As a reporter of transcriptional abundance, mCherry is either co-transcriptionally or co-translationally expressed from the same construct. Cells are flow-sorted into bins according to their EGFP/mCherry ratio, and deep sequencing is used to quantify each variant’s frequency in each bin. The EGFP/mCherry ratios of cells harboring each library spanned the range previously characterized for the wildtype (WT) and known destabilized variants. Finally, a stability score is calculated as the relative protein abundance based on the bin wise frequency. The relative protein abundance was computed, with 0 meaning unstable, 1 meaning wildtype stability, and >1 meaning more stable than the wildtype protein. The mean, standard deviation, upper and lower confidence intervals (CI) of the relative abundance was also recorded for each variant using replicates.

In the CAGI5 challenges for PTEN and TPMT, participants were asked to predict the relative protein abundance of the variants. A total of 8 teams participated in the challenge, submitting 16 different predictors.

In our analysis, we separately evaluated the performance on the PTEN and TPMT variants. For each gene, the predictions were compared against the experimental relative protein abundance means in a regression type analysis. Wildtype, synonymous and nonsense variants were removed from the dataset to limit our assessment to the missense variants only. Variants with mean relative abundance below 0, being outside the interpretable range, were also excluded from our analysis. For the classification type analysis, each variant was assigned either “pathogenic/destabilizing” or “benign/wildtype stabilizing” label based on its mean relative abundance. Variants having a value below 0.4 and between 0.4 (inclusive) and 1.2 were deemed “pathogenic” and “benign”, respectively. The scores above 1.2 were interpreted as more stabilizing than the wildtype and were not considered in the classification analysis. The previous assessment paper for the two challenges, used a different threshold based on the lower CI, upper CI and the mean.^20^ However, in our analysis, we use a simpler threshold based on the mean only. It is obtained after modeling the distributions of functional and nonfunctional variants using a multi-sample Gaussian mixture model (MSGMM) as proposed by Jain et al.^88^ We further refer the reader to Figure 1 from Pejaver et al.^20^ for the details of MSGMM modeling. Out of the total number of PTEN (TPMT) variants assayed, 3,716 (3,484) variants remained after removing the non-missense variants and those for which experimental values could not be measured. Out of these variants, 3,537 (3,228) variants made it to the classification set for PTEN (TPMT), having a proportion of 0.21 (0.12) pathogenic variants (data prior). For the clinical analysis, we used the data prior to compute the clinically relevant thresholds and all class-prior dependent measures. Although we have no evidence that the data prior probability reflects a population-based prior (as in the NAGLU challenge, where variants were selected from ExAC), this is still a useful quantity because the reference set of variants is essentially a set of all possible variants for the two genes. The results of the clinical analysis with the general diagnostic (0.1) and screening (0.01) priors are also reported. The predicted relative protein abundance was also used as a method score for the classification and clinical analysis. Our evaluation for the PTEN challenge is summarized in Main file: Figure 2, Figure S7 and Additional file 2: Table S2; for TPMT in Figure S1 and Additional file 2: Table S2.

### GAA

Acid alpha-glucosidase (GAA) is a lysosomal enzyme involved in the breakdown of glycogen. Some mutations in GAA cause Pompe disease (Glycogen Storage Disease II), a rare autosomal recessive metabolic disorder. BioMarin has functionally assessed the enzymatic activity of the 356 rare and novel missense mutations in from ExAC with transfected cell lysates. The fractional enzyme activity of each mutant protein compared to the wildtype enzyme was recorded such that a score of 0 means no activity, 1 means a wildtype-level of activity and >1 means greater than wildtype activity. Each mutant was assayed in at least three independent transfection experiments. The results from these determinations were averaged, and the standard deviation of experimental read-outs was calculated.

In the CAGI5 challenge for GAA, participants were asked to submit predictions for the fractional enzymatic activity of the variants. A total of 8 teams participated in the challenge, submitting predictions for 26 different models. The predictions were compared against the experimentally determined activity values in a regression type analysis. The experimental activity values were divided by 100 to scale them between 0 and 1 for consistency with other challenges. The predictions were already scaled between 0 and 1. In the most severe case zero GAA activity is observed in the patient’s fibroblasts; however, in less severe cases the activity is in the range of 25-30%. Taking a more conservative approach, we considered a variant “pathogenic” if its experimentally measured activity was less than 0.1, otherwise it was considered “benign”. The choice of the threshold is consistent with one of the thresholds used in the previous assessment of a submitted method for GAA.^92^ Based on the threshold of 0.1, 18% of the variants were labeled as pathogenic (data prior). For the clinical analysis, we used the data prior to compute the clinically relevant thresholds and all class-prior dependent measures. The results of the clinical analysis with the general diagnostic (0.1) and screening (0.01) priors are also reported. The predicted fractional activity was also used as the method score for the classification and clinical analysis. Our evaluation for the challenge is summarized in Figure S1 and Additional file 2: Table S2.

### CBS

CBS is a vitamin-dependent enzyme involved in cysteine biosynthesis. The human CBS requires two cofactors for function, vitamin B6 and heme. Homocystinuria due to CBS deficiency (OMIM #236200) is a recessive inborn error of sulfur amino acid metabolism. More than 90 different disease-associated mutations have been identified in the CBS gene.^93^ About one half of homocystinuric patients respond to high doses of pyridoxine and several alleles are clearly pyridoxine remediable: A114V, R266K, R366H, K384E, L539S and the frequent I278T which accounts for 20% of all CBS mutant alleles.

Jasper Rine’s lab at UC Berkeley collected 51 synthetic single amino acid variants for the CAGI1 challenge and 84 variants that had been observed in patients with homocystinuria for the CAGI2 challenge. The functionality of the variants was tested in an *in vivo* yeast complementation assay. The level of mutant human CBS function is measured in terms of the yeast growth in the assay. The rates are normalized as a percentage relative to wildtype (human) growth with the same amount of exogenous pyridoxine supplementation, plus and minus the standard deviation. Two concentrations of pyridoxine, high (400 ng/ml) and low (2 ng/ml), were used. A value of 0 indicates no growth, whereas 1 indicates a wildtype growth rate. In both CAGI1 and CAGI2 challenges for CBS, participants were asked to submit predictions for the effect of the variants in the function of CBS both in high co-factor (pyridoxine) concentration (400ng/ml) and in low co-factor concentration (2ng/ml). A total of 19 different predictors were submitted by 12 teams for the CAGI1 challenge and the same number of predictors were submitted by 9 teams for the CAGI2 challenge.

For our analysis, we created four datasets, CBS1Low, CBS1High, CBS2Low and CBS2High, separating CAGI 1 and 2 and the two co-factor concentration levels. Each of the four datasets was analyzed separately. For the regression type analysis, we evaluated the predictions against the experimentally measured normalized growth rates. The experimental and predicted growth rates were divided by 100 to scale them between 0 and 1 for consistency with other challenges. For the classification type analysis, a variant was interpreted to have “no growth” (positive class) if its experimental normalized growth rate was below 0.2, otherwise it was interpreted have “growth detected” (negative class). The threshold of 0.2 was determined as the lowest average growth observed in remediation variants in the CAGI 1 CBS dataset.^94^ Out of the 51 CAGI 1 CBS variants, 33% (39%) fraction were labeled “no growth” in presence of high (low) pyridoxine concentration. Out of the 84 CAGI 2 CBS variants, measurements could only be made on 78, out of which 51% (68%) were labeled “no growth” in presence of high (low) pyridoxine concentration. These percentages are the data priors for the four datasets. For the clinical analysis, we used the data prior to compute the clinically relevant thresholds and all class-prior dependent measures. The results of the clinical analysis with the general diagnostic (0.1) and screening (0.01) priors are also reported. The predicted relative growth rate was also used as the method score for the classification and clinical analysis. Our evaluation for the challenge is summarized in Figure S2A and S2B and Additional file 2: Table S2.

### SUMO ligase

The human genome encodes several small ubiquitin-like modifier proteins (SUMOs) that collectively ‘tag’ and modulate the functions of hundreds of proteins, including proteins implicated in cancer, neurodegeneration, and other diseases. As the only human SUMO-conjugating protein (SUMO E2 ligase), UBE2l is solely responsible for identifying target proteins and covalently attaching SUMO,^95^ thereby serving a very important function.

The Roth Lab has generated a library of over 6,000 UBE2I clones. These clones collectively express nearly 2,000 unique amino acid changes in various combinations, including several single substitutions. They have also implemented a yeast-based complementation assay in which expression of human UBE2I in *S. cerevisiae* rescues a temperature-sensitive mutant version of yeast UBC9. A library expressing mutant human UBE2I clones in yeast is grown competitively and quantified via DNA barcode sequencing to assess the functional impact of individual UBE2I variants. The growth scores are normalized relative to the growth of the clone considered to be wildtype. A growth score of 0 means that the mutant clone was completely ineffective, whereas a score of 1 means that it was as effective as the wildtype clones. The final growth score was obtained as the mean of the technical replicates for each variant; standard deviation was also recorded.

In the CAGI4 challenge for the SUMO ligase, participants were asked to submit predictions for the effect of the variants on the competitive growth. To help participants calibrate their numeric values appropriately, the experimental distribution of numeric growth scores was also provided. The variants were divided in three subsets; Subset 1: the high-accuracy subset of 219 single amino acid variants for which at least three independent barcoded clones are represented, providing internal replicates of the experiment; Subset 2: the remaining 463 (of 682 total) single amino acid variants; Subset 3: the additional 4,427 alleles corresponding to clones containing two or more amino acid variants. Participants were allowed to submit predictions for multiple subsets. A total of 16 different predictors were submitted by 9 teams for subset 1, 13 predictors from 7 teams for subset 2 and 12 predictors from 6 teams for subset 3.

We ran separate analyses on subsets 1 (SUMO1), 2 (SUMO2) and 3 (SUMO3). For each set, the predictions were assessed against the experimentally determined normalized growth rates. The previous assessment paper for the SUMO challenge^23^ labeled a variant as “deleterious”, if its growth rate was below 0.3; “intermediate”, if between 0.3 and 0.7; “wildtype”, if between 0.7 and 1.3 and “advantageous”, if above 1.3. In our assessment, we merged the “intermediate”, “wildtype” and “advantageous” variants into a single class “non deleterious”. In effect, if a variant had growth rate below 0.3, it was labeled “deleterious” (positive), otherwise it was labeled “non deleterious” (negative). Since the experimental growth rate could not be measured for many variants across the three subsets, the effective dataset size for the three subsets was 215 (subset 1), 410 (subset 2) and 3880 (subset 3). The proportion of “deleterious” variants was 41%, 48% and 67%, respectively (data prior). For the clinical analysis, we used the data prior to compute the clinically relevant thresholds and all class-prior dependent measures. The results of the clinical analysis with the general diagnostic (0.1) and screening (0.01) priors are also reported. The predicted growth rate was also used as method score for the classification and clinical analysis. Our evaluation for the challenge is summarized in Figure S3 and Additional file 2: Table S2.

### CALM1

Calmodulin is a calcium-sensing protein that modulates the activity of a large number of proteins in the cell. It is involved in many different cellular processes and is especially important for neuron and muscle cell function. Variants that affect calmodulin function have been found to be causally associated with two cardiac arrhythmias.

A team in Fritz Roth’s Lab at the University of Toronto and Lunenfeld Tanenbaum Research Institute (Sinai Health Systems), led by Jochen Weile and Song Sun, has assessed a large library of calmodulin variants using a high-throughput yeast complementation assay. The variants are assessed based on their ability to rescue a yeast strain carrying a temperature-sensitive allele of the yeast calmodulin orthologue CMD1.^96^ A fitness score was computed for each variant as competitive growth rate on a log scale and then normalized relative to the wildtype and nonsense variant scores such that a score of 0 means no growth at a restrictive temperature, whereas a score of 1 means wildtype growth. Technical replicates were used to measure the standard deviations.

In the CAGI 5 challenge for CALM1, participants were asked to submit predictions for the competitive growth scores of 1,813 variants. To help participants calibrate their numeric values appropriately, the experimental distribution of numeric growth scores was also provided. A total of 7 different predictors were submitted by 4 teams. The predictions were compared against the experimental growth scores in a regression type analysis. In the previous assessment paper for CALM1, a variant was labeled as “deleterious”, if its growth rate was below 0.3; “intermediate”, if between 0.3 and 0.8; and “neutral”, if above 0.8.^25^ In our analysis, we dropped the “intermediate” variants to create a binary classification dataset. In effect, the variants having growth rate below 0.3 were labeled “deleterious” (positive) and those with growth rate above 0.8 were labeled “neutral” (negative), respectively. Based on this criteria, 1,284 variants, were selected in the classification set, 21% of which were labeled “deleterious” (data prior). For the clinical analysis, we used the data prior to compute the clinically relevant thresholds and all class-prior dependent measures. The results of the clinical analysis with the general diagnostic (0.1) and screening (0.01) priors are also reported. The predicted growth rate was also used as a method score for the classification and clinical analysis. Our evaluation for the challenge is summarized in Figure S1 and Additional file 2: Table S2.

### Frataxin

Frataxin is a highly conserved protein found in prokaryotes and eukaryotes that is required for efficient regulation of cellular iron homeostasis. Humans with a frataxin deficiency have the cardio- and neurodegenerative disorder Friedreich’s Ataxia. The role of frataxin in cancer is still ambiguous; studies have shown that frataxin protects tumor cells against oxidative stress and apoptosis, but also acts as a tumor suppressor.^97, 98^

A library of eight missense variants, selected from the Catalog of Somatic Mutations in Cancer (COSMIC) database, were assessed by near and far-UV circular dichroism and intrinsic fluorescence spectra to determine thermodynamic stability at different concentration of denaturant. These data were used to calculate a ΔΔGH_2_0 value, the difference in unfolding free energy ΔΔGH_2_0 between the variant and wildtype proteins for each variant measured in kcal/mol.

In the CAGI5 challenge for Frataxin, participants were asked to submit predictions for the ΔΔGH_2_0 values of the 8 variants. A total of 10 different predictors were submitted by 6 teams. The predictions were compared against the experimental ΔΔGH_2_0 values in a regression type analysis. For the classification type analysis, variants having an experimental ΔΔGH_2_0 score ≤ −1.0 kcal/mol were labeled “destabilizing” (positive class), whereas those having score > −1.0 were labeled “neutral/stabilizing” (negative class). The choice of the threshold is consistent with the previous assessment paper for the challenge.^21^ Five out of the eight variants were labeled “destabilizing” in this manner. The predicted ΔΔGH_2_0 was also used as a method score for the classification type analysis. A clinical analysis was not performed for the challenge due to the small dataset size, which makes binning based local $lr^{+}$ and posterior estimates noisy. Our evaluation for the challenge is summarized in Figure S6 and Additional file 2: Table S2.

### PCM1

The PCM1 (Pericentriolar Material 1) gene is a component of centriolar satellites occurring around centrosomes in vertebrate cells. Several studies have implicated PCM1 variants as a risk factor for schizophrenia. Ventricular enlargement is one of the most consistent abnormal structural brain findings in schizophrenia.

The Katsanis lab assessed 38 missense mutations within PCM1 in a zebrafish model. Native zebrafish embryo PCM1 protein was suppressed by injecting morpholino (MO). The brain ventricle formation was measured for three groups suppressed PCM1 embryos: (1) injected with the human variant (MO+Var) or (2) injected with the wildtype mRNA (MO+WT) or (3) not injected with any mRNA (MO). The p-values for statistically different volume of brain ventricle between pairs of conditions were obtained using a Student’s t-test. When the p-value is:

- not statistically different from MO, but statistically significantly different from MO+WT, the variant is “pathogenic”.
- statistically different from MO, but not from MO+WT, the variant is “benign”*.*
- Statistically different from MO, and at the same time statistically significantly different from MO+WT, the variant is “hypomorphic” (partial loss of function).

In the CAGI5 challenge for PCM1 the participants were asked to submit the predictions for the p-values comparing ventricle size of MO+Var to MO and MO+WT groups. Additionally, the participants were also asked to submit the predictions for the class labels: pathogenic, benign and hypomorphic. A total of 6 different predictors were submitted by 5 teams.

Since no predictions for the relative change in the brain volume were solicited from the participants, a regression type analysis was not performed. For the classification type analysis, the p-value predictions were ignored since it is not obvious how two combine the two p-values into a single continuous method score for classification. Binary class labels were created from the three classes by merging the hypomorphic and pathogenic classes into a single pathogenic class and retaining the benign class as is. The merging was performed for both the true class labels derived from the experiment and the predicted class labels submitted by the participants. The merging resulted in 22 out of the 38 mutations (data prior: 58%) being assigned the pathogenic class based on the experiment. The evaluation was performed using the numeric class labels: 1 for pathogenic and 0 for benign. The numeric class label corresponding to the predicted class was interpreted as the method score. For the clinical analysis, we used the data prior to compute the clinically relevant thresholds and all class-prior dependent measures. The results of the clinical analysis with the general diagnostic (0.1) and screening (0.01) priors are also reported. Since the method scores were binary and discrete, local $lr^{+}$and posterior probability of pathogenicity are only defined at the two values taken by the method score, instead of being a continuous curve. Furthermore, the local $lr^{+}$ and the global$LR^{+}$ coincide in this case. Our evaluation for the challenge is summarized in Figure S4 and Additional file 2: Table S2.

### L-PYK

Pyruvate kinase (PYK) catalyzes the last step in glycolysis and is regulated by allosteric effectors. Defects in the glycolytic pathway due to PYK deficiency is a known cause for anemia. Isozymes of PYK expressed in the red blood cells (R-PYK) and the liver (L-PYK) are expressed from the same genes (pklr). The difference between R-PYK and L-PYK is minor and it appears to have no effect on enzyme function and regulation. However, L-PYK is easier to study in *E. coli* since 50% of R-PYK expressed in *E. coli* is truncated, whereas L-PYK is not similarly truncated. Several non-synonymous variants of R/L-PYK observed in PYK deficient patients fall in or near the allosteric effector binding sites. Therefore, modifications in allostery seem to be sufficient to cause disease. Two sets of variants were created by Aron Fenton at University of Kansas Medical Center. The first set of 113 variants were created by substituting the residues at nine sites in or near to the binding of the negative allosteric regulator, alanine. The second part of the challenge consisted of mutations to alanine at 430 sites throughout the protein. The variants were assayed in *E. coli* extracts for the effect on allosteric regulation of enzyme activity. The enzyme activity was recorded as a binary variable indicating presence (1) or absence (0). Allosteric effect was measured as the ratio (Qax) of apparent affinity in absence versus saturating presence of the effectors alanine and Fru-1,6-BP.

In the CAGI4 challenge for L-PYK the participants were asked to submit the predictions on the effect of mutations from the two sets on L-PYK enzyme activity and allosteric regulation. The prediction for enzymatic activity was interpreted as the probability of retaining enzymatic activity (a continuous score). A total of 5 different predictors were submitted by 4 teams for both the sets.

In our analysis, we disregarded the allosteric regulation predictions and only evaluated the predictors for the classification task of predicting enzymatic activity. We ran two separate classification analysis (LPYK1 and LPYK2) on the two sets. To make the interpretation of the positive class consistent with the other challenges we flipped the numeric labels so that 1 and 0 represents “absence” (positive) and “presence” (negative) of enzymatic activity, respectively. Presence or absence of enzymatic activity could not be measured for four variants from the second set, effectively reducing its size to 426. The percent of variants labeled as “absence” in the first and the second set was 20% and 10%, respectively. These percentages are the data priors for the two datasets. For the clinical analysis, we used the data prior, to compute the clinically relevant thresholds and all class-prior dependent measures. The results of the clinical analysis with the general diagnostic (0.1) and screening (0.01) priors are also reported. Our evaluation for the challenge is summarized in Figure S4 and Additional file 2: Table S2.

### p16

Coded by the CDKN2A gene, p16 is a tumor suppressor protein that acts as cyclin-dependent kinase inhibitor and is essential for regulating the cell cycle. Constitutional and inactivating p16 mutations are common in malignant melanoma. Saturation mutagenesis experiments were carried out to measure the cell proliferation rate on a few pivotal positions in the p16 protein and mutants at these positions were assayed along with some proband-related missense mutations (total 10 mutations). The proliferation rates of the mutation-like (positive) control cells was set as 100%. The proliferation rate for p16 wildtype (negative) control cells was approximately 50%. In this CAGI3 challenge, predictors were asked to assess the 10 p16 VUS for their ability to block cell proliferation. The challenge attracted 22 submissions from 10 groups.

In our analysis, we evaluated the methods on the regression-type prediction of the proliferation rates. Note that the original proliferation rates and their predictions were divided by 100 to give values between 0 and 1. For the classification type analysis, the variants with proliferation rates above 0.75 were labeled “pathogenic” and the remaining variants were labeled “benign”. The choice of the threshold is consistent with one of the three thresholds (0.65, 0.75 and 0.9) suggested by the data providers and used in the previous assessment paper for the challenge.^24^ Based on the threshold of 0.75, 5 out of the 10 variants were labeled “pathogenic”. The predicted proliferation rate was also used as the method score for the classification analysis. A clinical analysis was not performed for the challenge due to the small dataset size, which makes binning based local $lr^{+}$ and posterior estimates noisy. Our evaluation for the challenge is summarized in Figure S6 and Additional file 2: Table S2.

### p53 rescue

Known as the guardian of the genome, p53 is a central tumor suppressor protein that controls DNA repair, cell cycle arrest, and apoptosis (programmed cell death). Mutations in the p53 gene are the most recurrent genetic alterations in human cancers. Most of these alterations are of the missense type and show a very distinct distribution, localizing to the DNA-binding domain, and include several hotpots. Interestingly, a second mutation in p53 can in some cases rescue its function by altering a second amino acid that likely provides a structural change that compensates for the initial mutation. The aim of this challenge was to predict which second amino-acid change will rescue the p53 function. The dataset consisted of the exhaustive testing all 3,667 possible single amino acid change mutations in the entire core domain of p53 (194 amino acids from codon 96 to 289), in four different initial hit contexts; M237I, R248Q, R282W, Y220C. This amounts to a total of 14,668 mutations. The effect of the cancer rescue mutants was measured by wet-lab experimental assays of p53 function in yeast and/or human cell lines. A training set of 16,772 functionally characterized p53 mutants was also provided. In general, there are very few rescuing mutations–six mutations for M237I, one for R282W, one for Y220C and none for R248Q.

There were 8 predictions submitted from 5 different groups. Each submission provided a probability for rescue for each of the 14,668 mutations. We performed a classification type analysis to separate the “rescue mutations” (positives) from the remaining mutations (negatives). SWITCH was the best performing method with an AUC of 0.8. Of note, the approach for SWITCH uses both structural and conservation considerations as well as a more physics-based approach, which calculates stability of p53 by estimating the ΔΔG of the mutant vs. wildtype, looking for the changes that regain p53 stability. Regression and clinical analysis were not performed for the challenge. Our evaluation for the challenge is summarized in Figure S5 and Additional file 2: Table S2.

### BRCA

Mutations in the BRCA1 and BRCA2 genes increase the risk of breast and ovarian cancer. Myriad Genetics created the BRACAnalysis test in order to assess a woman’s risk of developing hereditary breast or ovarian cancer based on detection of mutations in the BRCA1 and BRCA2 genes. This test has become the standard of care in identification of individuals with hereditary breast and ovarian cancer (HBOC) syndrome. Myriad Genetics makes one of the following four classifications for a mutation:

1. Deleterious

2. Benign

3. Genetic Variant, Favor Polymorphism (VFP)

4. Variant of Unknown Significance (VUS).

These designations are based on a database of patient testing, including frequency of the variants in populations and segregation of variants with disease in families. Precisely, how Myriad Genetics assigns these designations, and their complete database of assignments, is proprietary. Nevertheless, using the BRACAnalysis test results from clinics, it was possible to determine these assignments for a set of 100 variants observed in patients. These variants and associated pathogenicity assessment were not found in the public domain.

In the CAGI 3 BRCA challenge, participants were asked to predict the probability that Myriad Genetics classified a variant to be deleterious for the 100 variants in the dataset. There were 14 predictions submitted from 5 different groups.

In our evaluation, we only considered deleterious and benign missense variants, i.e., variants labeled as VFP and VUS were removed, additionally, indels, truncated variants and intron mutations were also removed. The resulting set had 10 missense variants, 5 of which were deleterious and the other 5 were benign. Only a classification type analysis was performed. The top performing method^99^ had an AUC of 0.88. Our evaluation for the challenge is summarized in Figure S10B and Additional file 5: Table S5.

### ENIGMA

Breast cancer is the most prevalent cancer among women worldwide. The association between germline mutations in the BRCA1 and BRCA2 genes and the development of cancer has been well established, with mutations in these genes found in 1-3% of breast cancer cases. Testing for variation in these genes has emerged as a standard clinical practice, helping women to better understand and manage their heritable risk of breast and ovarian cancer. However, the increased rate of BRCA1/2 testing has led to an increasing number of variants of uncertain significance (VUS), and the rate of VUS discovery currently outpaces the rate of clinical variant interpretation. ENIGMA consortium (https://enigmaconsortium.org) is an international consortium focused on determining the clinical significance of sequence variants in BRCA1, BRCA2 and other known or suspected breast cancer related genes, providing expert input to global database and classification initiatives.

In the CAGI5 ENIGMA challenge, participants were asked submit predictions on 326 newly interpreted variants from the ENIGMA Consortium. Variants included in the dataset were classified according to the IARC 5-tier classification scheme using multifactorial likelihood analysis. The procedure assesses clinically calibrated bioinformatics information and clinical information (pathology, segregation, co-occurrence, family history, case-control) for each variant to produce a likelihood of pathogenicity. Likelihood values were calibrated against the features of known high-risk cancer-causing variants in BRCA1/2.^100, 101^ Each mutation was assigned to one of five classes depending in the pathogenicity likelihood, as shown in the table below. A combination of public and unpublished information was used to arrive at the final classifications, and all the classifications provided in the dataset for this challenge were either new or improved compared to what is in the public domain.^102^

Class                                          Probability of Pathogenicity

5: Pathogenic                           >0.99

4: Likely pathogenic                0.95-0.99

3: Uncertain                             0.05-0.949

2: Likely not pathogenic         0.001-0.049

1: Not pathogenic                   <0.001

Twelve predictions from 6 participating teams were submitted for this challenge. Four metrics were chosen for the assessment: ROC AUC, precision/recall AUC, precision and recall.^27^ The rank order was largely consistent between metrics. The best-performing method used feature categories including splice predictions, population frequencies, conservation scores, and clinical observation data, such as personal and family history and covariant information.^103^ The population frequencies, leveraged from gnomAD, were instrumental in many accurate predictions, as was the splicing information, a feature also used successfully by another team.

In our analysis for the challenge, we derived a binary class label from the original class label. Classes 5 and 4 were merged to create a single “pathogenic’ (positive) class, classes 1 and 2 were merged to create a single “benign” (negative) class, and the variants from class 3 were not included in the analysis. The resulting dataset had 321 variants, out of which 17 were labeled “pathogenic”. In absence of any continuous experimental measurement, we only perform the classification type analysis. Our evaluation for the challenge is summarized in Figure S10A and Additional file 5: Table S5.

### Annotate All Missense

dbNSFP is a database of human nonsynonymous single nucleotide variants (nsSNVs) and their functional predictions and annotations.^54, 104-106^ Version 3.5 compiles 18 functional prediction scores and 6 conservation scores, as well as other related information including allele frequencies observed in different large datasets, various gene IDs from different databases, functional descriptions of genes, gene expression and gene interaction information.

For this CAGI5 challenge, a large list of possible SNVs based on the human reference sequence was created from dbNSFP. This resulted in 81,084,849 possible protein-altering variants. Predictors were asked to predict the functional effect of each of these coding SNVs. For the vast majority of these missense and nonsense variants, the functional impact is not known, but experimental and clinical evidence is accruing rapidly. Rather than drawing upon a single discrete dataset as typical with CAGI, predictions were assessed by comparing with experimental or clinical annotations made available after the prediction submission date. If predictors provided their assent, predictions would also be incorporated into dbNSFP.

A test dataset of newly annotated missense variants was constructed from ClinVar and HGMD databases, considering only variants added to these databases between June 2018 (after the close of the annotate all missense CAGI5 challenge) and December 2020. In particular, the June 3, 2018 and December 26, 2020 releases of ClinVar were obtained and variants annotated as missense SNVs were extracted. Similarly, 2019.1 (first quarter of 2019) and 2020.4 (last quarter of 2020) releases of HGMD were obtained and restricted to missense SNVs. A set of newly annotated ClinVar missense variants were obtained by subtracting from the December 26, 2020 release any variants present in the June 3, 2018 release, except those with a clinical significance annotation of “Uncertain significance” in the June 3, 2018 release, as well as any variants present in HGMD 2019.1. Any variant with a review status of “no assertion provided”, ‘’no assertion criteria provided”, “no interpretation for the single variant” and “conflicting interpretations” was removed from the set. A set of newly annotated HGMD variants was generated by subtracting from the HGMD 2020.4 release any variants present in HGMD 2019.1, as well as any variants present in the June 3, 2018 ClinVar release, except those with a clinical significance annotation of “Uncertain significance.”  Subtraction was done based on the chromosome and position of the variant in each case. In total, there were 3,309 pathogenic (P+LP) ClinVar variants, 2,732 of which were likely pathogenic (LP), 10,677 disease mutations (DM; 1,141 of which DM?) from HGMD, and 23,096 benign variants (B+LB); 11,078 of which likely benign, (LB) from ClinVar. The newly annotated ClinVar and newly annotated HGMD variants were combined to generate two test sets, 1) AAM1All: containing only variants with confident assertions (“pathogenic” or “benign” in ClinVar or “DM” in HGMD) and 2) AAM2All: additionally, containing variants with less confident  assertions (“pathogenic,” “pathogenic/likely_pathogenic,” or “likely_pathogenic” in ClinVar; “DM” or “DM?” in HGMD; “benign”, “benign/likely_benign,” or “likely_benign” in ClinVar). We used these test sets to evaluate performance of the four submitted predictors for this challenge. Additionally, all tools (functional predictions and conservation scores) with results deposited in dbNSFP v3.5 were also evaluated, as a set of commonly available tools that could not have been trained on the test variants, since dbNSFP v3.5 was released in 2017. In all, the following tools were included in the evaluation: VEST, Turkey, Bologna, Condel, SIFT, PROVEAN, PolyPhen-2, LRT, MutationTaster, MutationAssessor, FATHMM, CADD v1.4, fitCons, DANN, MetaSVM, MetaLR, GenoCanyon, Eigen, M-CAP, REVEL, MutPred, GERP++, phyloP, phastCons and SiPhy. Some of these tools have multiple prediction scores, included in the analysis; for example, VEST has versions 3 and 4 predictors and FATHMM has version 2.3 and fathmm-mkl.

Only classification and clinical analysis was performed for this challenge. For the clinical analysis, we used the general diagnostic (0.1) and screening (0.01) priors, to compute the clinically relevant thresholds and all class-prior dependent measures. In addition to AAM1All and AAM2All, evaluation was performed on the following six data subsets created from the two sets. (1) AAM1BiClass: containing variants restricted to the “bi-class” genes that have both pathogenic and benign variants in AAM1All, (2) AAM2BiClass: containing variants restricted to the “bi-class” genes that have both pathogenic and benign variants in AAM2All, (3,4) AAM1CV and AAM2CV: composed of subsets of AAM1All and AAM2All data, respectively, restricted to the variants from ClinVar only and (5,6) AAM1HGMD and AAM1HGMD: containing subsets of AAM1All and AAM2All data, respectively, with the pathogenic variants only coming from HGMD. A total of 22,131 variants from 6,482 genes were present in the AAM1All dataset, out of which 7,429 variants came from 1,022 bi-class genes. A total of 37,082 variants from 7,723 genes were present in the AAM2All dataset, out of which 21,423 variants came from 2,074 bi-class genes. Since all the datasets created from AAM2All, include less confident variant classes, they are more difficult to predict compared to those created from AAM1All. Within all AAM1All (and AAM2All) generated datasets the bi-class gene dataset is the most difficult to predict. The evaluation for the challenge is summarized in Main file: Figure 3, Figures S7 and S9 and Additional file 4: Table S4.

## Expression and Splicing Challenges

### Vex-seq

In the CAGI5 challenge, Vex-seq, a barcoding approach, Variant exon sequencing (Vex-seq), was applied to assess the effect of around 2,000 natural single nucleotide variants and short indels on splicing of a globin mini-gene construct transfected into HepG2 cells. The results are expressed as ΔΨ (delta PSI, or Percent Spliced-In), between the variant Ψ and the reference Ψ. If ΔΨ is calculated from a reference exon that is always spliced in (Ψ(reference) = 100), and a variant exon that is only spliced-in in half of the transcripts observed for that variant (Ψ(variant) = 50), the ΔΨ would be 50. ΔΨ is bounded by −100 and 100. A training set of around 1,000 variants containing the ΔΨ values were provided to the participants. Participants were asked to predict the ΔΨ values for the remaining 1,098 test variants. A total of six groups participated in the challenge.

In our analysis, for this challenge, we created two datasets: one for over-splicing (VexSeq1) and the other for under-splicing (VexSeq2). For VexSeq1, a variant was assigned an “over-splicing” (positive) label, if its experimental ΔΨ value was more than one standard deviation (13.56) above the mean ΔΨ (−1.88) value, the remaining variants were considered as negatives. Similarly, for VexSeq2, a variant was assigned an “under-splicing” (positive) label, if its experimental ΔΨ value was more than one standard deviation below the mean ΔΨ value, the remaining variants were considered as negatives. Creating the class labels, using the mean and the standard deviation was done based on the previous assessment paper for the challenge.^57^ A regression type analysis was performed, comparing the predicted $\Delta\Psi$ value to the experimental values. The data for the regression analysis was identical for VexSeq1 and VexSeq2. Some participants submitted predicted ΔΨ in the range from −1 to 1. We multiplied the predictions by 100 in such cases. 6.5% and 7.5% of the variants were labeled as over-splicing and under-splicing in VexSeq1 and VexSeq2, respectively. The predicted $\Delta\Psi$ was also used as the method score for the classification type analysis. A clinical analysis was not performed for the challenge. Our evaluation for the challenge is summarized in Figure S11 and Additional file 6: Table S6.

### MaPSy

In the CAGI 5 challenge, the Massively Parallel Splicing Assay (MaPSy) approach was used to screen sets of 4,964 and 797 reported exonic disease mutations using a mini-gene system, assaying both *in vivo* via transfection in tissue culture, and *in vitro* via incubation in cell nuclear extract. The loss or gain of splicing efficiency was measured in terms of log_2_ allelic skew ratio computed from the read counts of input DNA and correctly spliced cDNA for the variant and the wildtype. The log_2_ ratios are expected to be 0 for a neutral mutation, negative for under-splicing and positive for over-splicing. The variants were categorized as exonic splicing mutations (ESMs) if they both changed the allelic ratio by 1.5‐fold or more and passed a two‐sided Fisher’s exact test with a false discovery rate (FDR) of 5% both *in vivo* and *in vitro*. The set of 4,964 variants along with the measurements of the log_2_ allelic skew ratios (*in vivo* and *in vitro*) and the ESM class labels were provided to the participants for training their models. The challenge was to predict the log_2_ allelic skew ratios (*in vivo* and *in vitro*) and the ESM class labels on the test set of 797 variants. The participants were asked to submit their predictions for the two log_2_ allelic ratios and the variants’ probability of being an ESM. A total of five groups participated in the challenge.

For this challenge, we created three datasets, MaPSy1, MaPSy2 and MaPSy3, to be analyzed separately. MaPSy1 and MaPSy2 included the log_2_ allelic skew ratios measured in vivo and in vitro, respectively, whereas MaPSy3 included the ESM class labels. A regression type analysis was performed on MaPSy1 and MaPSy2 for predicting the *in vivo* and *in vitro* log_2_ allelic skew ratios, respectively. A classification type analysis was performed on MaPSy3 for the predicting the ESM class label. The *in vivo* and *in vitro* allelic ratios did not agree (implying opposite effects on splicing) for some of the variants. The challenge assessors performed a “consistent” analysis where the disagreeing variants were relabeled “non ESM”. Following that approach, the ESM class label for such variants, if originally set to 1 (ESM), were changed to 0 (non ESM).^57^ In this manner, labels were changed for 19 variants. Only 30 out of the 797 variants were finally labeled ESM in MaPSy3. The predicted probability of ESM was used as the method score for the classification task. A clinical analysis was not performed for the challenge. Our evaluation for the challenge is summarized in Main file: Figure 4 and Additional file 6: Table S6.

### eQTL

Genome-wide association studies (GWAS) suggest that much of the variation underlying common traits and diseases maps within regions of the genome that do not encode protein. However, identifying the causal alleles responsible for variation in expression of human genes has been particularly difficult. In the CAGI4 eQTL causal SNPs challenge, a massively parallel reporter assay (MPRA) was applied to thousands of single nucleotide polymorphisms (SNPs) and small insertion/deletion polymorphisms in linkage disequilibrium (LD) with cis-expression quantitative trait loci (eQTLs). The results identify variants showing differential expression between alleles. The challenge is to identify the regulatory sequences and the expression-modulating variants (emVars) underlying each eQTL and estimate their effects in the assay.

The CAGI4 eQTL challenge comprised two parts. In the first, 3,006 potential regulatory sequences and variants (2,811 SNVs and 195 indels) associated with a distinct subset of 1,050 eQTLS were provided. Participants were asked to predict the level of transcriptional activity for each allele and to determine for each variant whether at least one of the alleles is a “regulatory hit” (positive class), based on significant activation of reporter gene expression. 12% of the variants were labeled as regulatory hit. A sample dataset of 3,044 variants associated with 1,052 eQTLs was provided for training. In the second part of the challenge, 401 regulatory sequences (370 SNVs and 31 indels) associated with a third distinct subset of 1,055 eQTL were provided. Participants were given variants that were confirmed regulatory hits and asked to predict the difference between the transcriptional activity of the two alleles, both quantitatively as the log_2_ allelic skew (the log_2_ ratio of expression level of the alternative allele relative to the reference allele) and qualitatively as expression-modulating variant, “emVar” (positive class). 26% of the variants were labeled as emVar. Seven groups participated in this challenge, submitting 20 predictions for the first part, and 13 submissions for the second. The prior assessment of this challenge identified chromatin accessibility and transcription factor binding as features leading to the most accurate results.^59^

For the first part of the challenge (eQTL1), we evaluated the methods only on the classification task of predicting if a variant is a regulatory hit, using the predicted probability of regulatory hit as the method score. For the second part of the challenge (eQTL2), we evaluate the methods on the regression task of predicting the log_2_ allelic skew and the classification task of predicting if a variant is an emVar. The predicted probability of emVar (different from the predictions for log_2_ allelic skew) is used as the method score for the classification task. A clinical analysis was not performed for the challenge. Our evaluation for the challenge is summarized in Figures S12A and S12B and Additional file 6: Table S6.

### Regulation-Saturation

Gene regulatory variants are known to play an important role in a number of common human diseases, including diabetes, neuropsychiatric disorders, autoimmune disorders, cardiovascular disease, and cancer. These variants modulate the strength of interactions between enhancers and promoters and the transcription factors (TFs) that bind them and alter the cell-specific transcriptional control of gene regulatory networks central to the proper development and functioning of human cells and tissues. Although we have a good basic understanding of the general molecular mechanisms of these interactions, quantitative and predictive models of cell-specific enhancer and promoter function are currently under active development.

In this CAGI5 challenge, 17,500 single nucleotide variants (SNVs) in 5 human disease associated enhancers (IRF4, IRG6, MYC, SORT1, ZFAND3) and 9 promoters (TERT, LDLR, F9, HBG1, PKLR, MSMB, HBB, HNF4A, GP1BB) were assessed in a saturation mutagenesis massively parallel reporter assay (MPRA) in different cell lines; see Kircher et al.^107^ for a more detailed description of the MPRA experimental methods. Promoters were cloned into a plasmid upstream of a tagged reporter construct, and reporter expression was measured relative to the plasmid DNA to determine the impact of promoter variants. Enhancers were placed upstream of a minimal promoter and assayed similarly. A multiple linear regression model fitting the reporter’s log expression level with binary (dependent) variables, one corresponding to each variant, is used to estimate the contribution of a variant as its (fitted) coefficient. A confidence score was derived for each coefficient after scaling its p-value on a $\log10$ scale and normalizing to a 0-1 range. Effectively, a confidence score of 1 corresponds to a p-value of ≤10-50, 0.5 to 10-25 and 0 to a p-value of 1. The coefficient served as a continuous measurement capturing a variant’s effect on expression. A ternary class variable was derived for each variant taking value −1 (decrease expression) or 1 (increase expression), if the coefficient is negative or positive, respectively, and the confidence is greater than 0.1 (p-value of 10^−5^), otherwise the variable was set to 0 (no effect on expression). Participants were given the impact of the variants (coefficients and ternary labels) in selected subsets from each region to train their models, consisting of around 25% of the variants. The remaining variants were used for evaluation. The challenge is to predict the functional effects of these variants (coefficients and the ternary labels) in the regulatory regions as measured from the reporter expression. The participants were only required to submit discrete values: −1 (decrease expression), 0 (no effect on expression) and 1 (increase expression) for each variant, along with a probability that the discrete values are correctly assigned. However, we were, additionally, able to procure the continuous prediction for a variant’s effect on expression, obtained during the previous assessment of the challenge for the best methods from the top three performing groups.^60^

According to the data providers, the dataset incorrectly included positions 20bp up and downstream of each construct due to technical reasons. These variants were identified by the positions listed ahead were removed from the training and test datasets. F9:X 138612621; GP1BB:22 19710788; HBG1:11 5271309; HNF4A:20 42984159; IRF6:1 209989736; MSMB:10 51548987; PKLR:1 155271656; SORT1:1 109817273; HBB:11 5248439; IRF4:6 396142; LDLR:19 11199906; MYCrs6983267:8 128413073; ZFAND3:6 37775274.

Seven groups participated, with a total of 23 submissions. All top performing models for variant impact prediction used machine learning based ANN (or gkm-SVM) DNA sequence features trained on chromatin accessibility or chromatin state data.^60^ These models consistently outperformed models using sequence features derived from other sources (evolutionary conservation, kmers, or more generic sequence features, e.g., GC content). The machine learning-based models also outperformed models using chromatin accessibility, chromatin state, or TF ChIP-seq data without using epigenomic data to derive DNA sequence-based models. High prediction accuracy was obtained when machine learning-based DNA sequence features were combined with proper importance weighting derived from another layer of machine learning on a subset of the mutation data used as training for each cell type.

In our analysis, the classification tasks corresponded to predicting a set of binary labels derived from the ternary labels and the regression analysis corresponded to the prediction of the fitted coefficients, measuring a variants’ effect on expression. We evaluated the performance on the enhancer and promoter variants separately. From a total of 13,790 variants available in the test set, 6,295 and 6,868 corresponded to the enhancers and promoters, respectively. From each set, we created two binary classification datasets to separately evaluate the performance on predicting the increase and decrease in expression. For the increase in expression the variants with the ternary class label 1 were considered as positives and the remaining variants as negatives. Similarly, for decrease in expression, the variants with the ternary class label −1 were considered as the positives (relabeled as 1) and the remaining variants as negatives. In total we perform four separate analyses: RegSatEnh1 (increase), RegSatEnh2 (decrease), RegSatProm1 (increase) and RegSatProm2 (decrease), each with both regression and classification type evaluation. Given the enhancers or the promoters, the regression type task in both the increase and decrease of expression analysis is the same: evaluate the predictions for a variant’s contribution to the reporter’s expression level. The two analyses differ only w.r.t to the classification task since only the class labels are different. The proportion of positives for the increase in expression were 0.09 and 0.06 for the enhancers and promotors, respectively. The proportion of positives for the decrease in expression were 0.14 and 0.13 for the enhancers and promotors, respectively. The discrete predictions were used for both the regression and classification type analyses for most methods, except the three methods, for which continuous predictions were procured. A clinical analysis was not performed for the challenge. Our evaluation for the challenge is summarized in Main file: Figure 5 and Additional file 6: Table S6.

## Complex disease challenges

This work re-assessed only a single complex disease challenge.

### Crohn’s disease

Crohn’s disease (CD; MIM #266600) is a chronic inflammatory bowel disease (IBD) characterized by relapsing inflammation that can involve any part of the gastrointestinal tract and also extra-intestinal manifestations. It is caused by the complex interplay between an overly active immune system and environmental triggers in genetically susceptible individuals. Results from twin and familial aggregation studies,^108^ as well as evidence from GWAS,^109, 110^ have shown that genetic factors play an important role in CD etiology. To date, 163 genetic susceptibility loci have been identified for IBD with 30 loci exclusive to CD, 23 to ulcerative colitis (UC), and 110 shared by the two.^110^ Early-onset cases of IBD, with an age of onset before 10, often show a more severe disease course with a higher risk of complications, and genetic factors likely play a larger role in these individuals.^111^

Three successive iterations of this challenge were performed. The 2011 (CAGI2) dataset had 56 exomes (42 cases, 14 controls), all of German ancestry.^112^ During assessment, substantial batch effect was discovered in the data as a side effect of sample preparation and sequencing.^29^ The 2013 (CAGI3) dataset had 66 exomes (51 cases, 15 controls). Although these samples were also of German ancestry, cases were selected from pedigrees of German of families with multiple occurrences of Crohn’s disease. As such, some of these cases were related. This led to a substantial difference in clustering between cases and controls, suggesting the presence of sampling bias.^29^ The 2016 (CAGI4) challenge had 111 unrelated German ancestry exomes (64 cases, 47 controls). For the CAGI4 challenge, submitting groups were allowed to use the data from the CAGI2 and CAGI3 Crohn’s challenges for training. In all iterations of the challenge, groups were asked to report a probability of Crohn’s disease (between 0 and 1) for each individual and a standard deviation representing their confidence in that prediction. For the CAGI4 challenge, teams were also asked to predict whether age of onset was greater or less than 10 years of age. The problems with batch effects and sampling bias were no longer present in the CAGI4 Crohn’s challenge.^29^ This was the challenge selected for further analysis in this study.

We analyzed the CAGI4 Crohn’s data on classification problem of separating the cases (positives) from the controls (negatives). In addition to the ROC curve, AUC and the local $lr^{+}$ curve, we also give the RR (relative risk) curve and the kernel density estimation-based distribution of the method scores for the cases and controls. A class-prior of 1.3% is used to compute the RR curve with Equation 31 (see Methods). The choice of the prior was based on the recent data on the prevalence of inflammatory bowel disease in US adults.^66^ Our evaluation for the challenge is summarized in Main file: Figure 6 and Additional file 7: Table S7.

# Non-analyzed challenges

Summaries of challenges that were not analyzed for this work are presented below in alphabetical order.

**Asthma twins (CAGI2).** The dataset includes whole genomes of 8 pairs of discordant monozygotic twins (randomly numbered from 1 to 16) that is, in each pair identical twins one has asthma and one does not. In addition, RNA sequencing data for each individual is provided. One of the twins in each pair suffers from asthma while the other twin is healthy.

There were 6 submissions from 6 groups. All predicted the correct twin pairs but the asthma correction rate was 63%, no better than random. In the genomic data, the number of errors was greater than the number of variants. This sequencing error rate might have masked the differences between the twins in the genomic data. Further, the RNA sequencing data appeared to correlate with the twins, rather than with the disease status. The experimental dataset remains unpublished, and thus the results are not further discussed here.

**Bipolar exomes (CAGI4).** This challenge involved the prediction of which of a set of individuals have been diagnosed with bipolar disorder, given exome data. 500 of the 1000 exome samples were provided for training. Nine groups participated in this challenge, providing 29 submissions. No participant was very successful, with the highest AUC being 0.64.^29^ Although not impressive, the best-scoring method is interesting. While most participants used similar approaches to those deployed for Crohn’s, this one used linear genotype status as an input vector to a three-layer neural network, trained on the data provided, and used no information about the disease or known GWAS loci. The result suggests that more sophisticated machine learning approaches have potential in this area. A caveat is that the case and control data were from different sources, so it is possible that the method identified some underlying sequence features not related to the disease.

**Breast cancer pharmacogenomics (CAGI2).** Cancer tissues are specifically responsive to different drugs. For this experiment, predictors are asked to predict the response of each of 54 breast cancer cell lines to a panel of 54 drugs. Data about the tissues include transcriptional profiling, SNP data and copy number profiles measured for cells grown in the absence of any treatment. The prediction requested was GI50 values with standard deviation.

Three groups participated, providing 3 submissions. The assessors used RMSE and Kendall’s tau to evaluate predictions. RMSE was used to measure how well each submitted prediction estimated the overall level of sensitivity to a particular drug, while Kendall's tau was used to measure the quality of cell line rankings from least to most sensitive, as reported by each method. All three submissions performed significantly better than random on Tamoxifen, Bortezomib, and Iressa. Additionally, submission SID#16A had the lowest RMSE on 10 of the 15 drugs. Kendall's tau was low overall (<0.3) and no algorithm was able to accurately rank the cell lines from least to most sensitive.

**CHEK2 (CAGI1, CAGI5).** Cell-cycle-checkpoint kinase 2 (CHEK2; OMIM #604373) is a protein that plays an important role in the maintenance of genome integrity and in the regulation of the G2/M cell cycle checkpoint. CHEK2 has been shown to interact with other proteins involved in DNA repair processes such as BRCA1 and TP53. These findings render CHEK2 an attractive candidate susceptibility gene for a variety of cancers. The challenges in both CAGI1 and CAGI5, involved classifying variants as occurring in breast cancer cases or controls.

In CAGI1, predictors were provided with 41 rare missense, nonsense, splicing, and indel CHEK2 variants. Ten groups participated in this challenge, making a total of 16 submissions. Assessment showed several methods performing better than the baseline method (Align-GVGD), which had been trained on this dataset. Functional contribution to the predictions is particularly helpful when evolutionary information is not discriminative enough. Participants tended to not properly consider the likely distribution of neutral mutations. A probability of 0.5 would indicate that the mutation is neutral (equal in both populations) while a probability of less than 0.5 would be indicative of a variant that is actually protective.

In CAGI5, data involved the targeted resequencing of CHEK2 from approximately 1000 Latina breast cancer cases and 1000 ancestry matched controls. Fifty-three variants in the list, observed between 1 and 20 times, were provided for this challenge. Eight groups participated, with a total of 18 submissions. While group 5, submission 1, appeared to do best overall, it had many false positives. Additionally, most of the variants were found in cases, and methods that favored this performed better.^113^

**Clotting disease (DVT or PE) exomes** **(CAGI5).** African Americans have a 30-60% higher incidence of developing venous thromboembolisms (VTE), which includes deep vein thrombosis (DVT) and pulmonary embolism (PE) than people of European ancestry.^114^ The risk factors for VTE are complex and include environmental risk factors (e.g., vessel injury; and blood stasis) and genetic risk factors, including common and/or rare variants that predispose to hypercoagulation.^115^ In this challenge, participants are provided with exome data and clinical covariates for a cohort of African Americans who have been prescribed Warfarin, an anticoagulant, either because they had experienced a VTE event or had been diagnosed with atrial fibrillation (which predisposes to clotting). The challenge requested participants to distinguish between these conditions.

Seven groups participated in this challenge, providing 16 submissions. Assessment was complicated by two factors. First, the warfarin doses of study individuals were known to participants, and VTE patients are usually given high doses, providing a strong predictive signal that a number of participants exploited. In hindsight, as noted by the assessors, given the strong genetic relationship between warfarin dosage and genetics, it may have been better for the challenge to not provide warfarin dosage to the participants and to remove genes related to warfarin pharmacokinetics and pharmacodynamics from the exomes.^116^ Second, unlike Crohn’s disease and Bipolar disorder, studies in Europeans in the UK Biobank have calculated the heritability on the liability scale in Europeans to be 0.14 and disease prevalence to be 2%, which indicate that the theoretical maximum AUROC that could be achieved in predicting VTE from coding regions is a low 0.62.^117, 118^ The best AUC from methods that did not appear to use warfarin dose information was 0.65, while a previously published baseline method developed for European populations^119^ did better than any submitted method that did not use warfarin dose in their predictions, with an AUC of 0.71.

**FCH (CAGI3).** Familial combined hyperlipidemia (FCH; OMIM 14380) the most prevalent hyperlipidemia, is a complex metabolic disorder characterized by variable occurrence of elevated low-density lipoprotein cholesterol (LDL-C) level and high triglycerides (TG)—a condition that is commonly associated with coronary artery disease (CAD).

The challenge involved exome sequencing data for 5 subjects in an FCH family and comprised two parts. In the first, participants were given which family members have elevated LDL and asked to predict which variant(s) confer the elevated LDL phenotype. In the second part of the challenge, the task was to predict which individuals have abnormally high TG and which individuals have abnormally high HDL levels.

There were 21 submissions from 11 groups. The assessor considered the first part of the challenge very simple, as the presumably causal mutation is listed in OMIM and most people would check LDLR. Several submissions did well in this task, with three groups uniquely predicting the most probable diagnostic mutation. Two submissions only listed the correct mutation, while a third listed two others but with negligible confidence placed on them.

The second part of the challenge was considered hard, and there were no correct submissions. The assessor commented that predicting the abnormal father’s TG and HDL is very hard, so mostly required that the unaffected daughter would be predicted correctly. There were three reasonable submissions. Combining two sub-challenges was difficult. Only one submission did reasonably in both cases. Judging solely on the first sub-challenge, three submissions did well.

**HA (CAGI3).** Hypoalphalipoproteinemia (HA; OMIM #604091) is characterized by severely decreased serum high-density lipoprotein cholesterol (HDL-C) levels and low apolipoprotein A-1 (APOA1). Low HDL-C is a risk factor for coronary artery disease. The dataset for this challenge comprises of exome sequencing data for 4 subjects from the same family, one of which has HA.

The challenge attracted 18 submissions from 12 groups. While 3 submissions confidently identified the proband, no group provided the right answer with high confidence. One group had good correlation between probability of illness and actual disease state, by making a series of ‘bets’ about likely priors. Here again, data quality complicated interpretation and assessment.

**Johns Hopkins clinical panel (CAGI4).** The Johns Hopkins challenge, provided by the Johns Hopkins DNA Diagnostic Laboratory (http://www.hopkinsmedicine.org/dnadiagnostic), comprised of exonic sequence for 83 genes associated with one of 14 disease classes, including 5 decoys.^61^ Participants were tasked with identifying the disease class for each of 106 patients; 43 of these patients had received a molecular diagnosis in the clinical pipeline.

Five groups participated, providing a total of 5 submissions. The most successful CAGI method correctly matched 36 of the previously diagnosed patients to their disease class. More interestingly, 39 of the 63 undiagnosed cases were successfully matched by at least one participating group, indicating successful identification of causative variants. Some of these may have been highlighted in the John Hopkins pipeline, but did not have strong enough evidence to meet the ACMG/AMP guidelines.^32^ Guidelines also require the official pipeline only search for causative variants in genes consistent with the specific disease a physician requested a test for. For a number of undiagnosed cases, CAGI participants found high-confidence deleterious mutations in genes that were not in the selected panel, suggesting physicians may have misdiagnosed the symptoms.^61^ However, because of the IRB guidelines under which the pipeline operates, it has not been possible to further investigate or even publicly report these cases. Ensuring appropriate consents and approvals are in place in advance of a challenge, could maximize the use of clinical data, and allow for a more in-depth and critical analysis of challenge results.

**Intellectual disability panel (CAGI5).** In the ID challenge, 150 individuals with ID and/or Autism Spectrum Disorder (ASD) were assessed through sequencing of 74 genes involved in ID with or without autistic features. Predictors were tasked with matching patients with one or more of 7 phenotypes and identifying causative or contributing variants for each patient.

Five groups participated in this challenge, submitting a total of 15 predictions. The phenotype matching in this challenge had a poor overall performance, with the top method achieving 0.78 for the ID phenotype. While the Hopkins panel was testing for monogenic diseases with Mendelian inheritance, the ID challenge addressed complex disease. The genetic bases of neurodevelopmental disorders (NDDs) are not fully understood but are characterized by strong clinical comorbidity as well as genetic heterogeneity, involving the interplay of de novo, rare, and many common variants which affect phenotype variability and disease severity. Despite these difficulties, some groups made plausible predictions on novel variants that had not been reported to the patient due to being predicted neutral by the majority of standard computational methods.^62^ In two of these cases, the proband phenotype was partially consistent with the clinical observations, and segregation analysis showed the variants to be absent from the mother and healthy sibling, suggesting they might be transmitted *in cis* from the other parent. However, the father was not available for further investigation.

**Mouse exomes (CAGI2).** The challenge involved identifying the causative variants leading to one of four morphological phenotypes arising spontaneously in inbred mouse lines (L11Jus74, Sofa, Frg and Stn. Predictors were given SNVs and indels found from exome sequencing. Causative variants had been identified for the L11Jus74 and Sofa phenotypes by the use of traditional breeding crosses,^120^ and the predictions were compared to these results, which were unpublished at the time of the CAGI submissions. The L11Jus74 phenotype is caused by two SNVs (chr11:102258914A>T and chr11:77984176A>T), whereas a 15-nucleotide deletion in the Pfas gene is responsible for the Sofa phenotype.^9^ The predictions for Frg and Stn phenotypes could not be compared to experimental data, as the causative variants could not successfully be mapped by linkage.

There were 2 submissions from 2 groups. The approach of the first team consisted of two steps: (1) searching the JAX Mice Database (https://mice.jax.org) for chromosome regions where the phenotypes are known to map; (2) examining the effects of missense variants located in the found regions with the help of MutPred^99^ and SNPs&GO.^121^ In addition, variants in the proximity of splice sites, determining a frameshift or a stop gain/loss in the coding sequence were included in the submission. Altogether the group submitted seven candidate variants for L11Jus74, and four variants for the Sofa phenotype. None of the predicted variants coincided with the causative variants identified. The second team also utilized the JAX Mice Database and assigned predicted effects for all types of variants. For the L11Jus74 phenotype, they only considered variants in chromosome 11, assuming that the phenotype name implied a causal variant in that region. Their submission included 82 candidate variants for L11Jus74, and 31 for the Sofa phenotype. They were able to identify the linkage matching variants for both phenotypes and assigned these with the highest probabilities. However, because of the large number of possible variants included, the absolute probabilities are low (p = 0.0195 and 0.0586 for L11Jus variants; p = 0.0541 for the Sofa variant). These limited results indicate that causative monogenic-like variants can be identified with current methods, though perhaps not unambiguously.

**MR-1 (CAGI2, CAGI3).** *Shewanella oneidensis* strain MR-1 (formerly known as *S. putrefaciens*) is a model organism for studying metal reduction, as MR-1 can utilize a wide range of metal ions and solid metals as electron acceptors and also grows aerobically. MR-1 is in the same division of bacteria as *E. coli* (the γ-Proteobacteria), but they are not closely related. Of the ~4,500 proteins in MR-1, only about a third have orthologs in *E. coli*.

The Arkin Lab at UC Berkeley created a large number of *S. oneidensis* MR-1 transposon insertions with known location and with a known tag or barcode. These insertions were pooled together into two pools, and the pools grown under a given (stress) condition for ~6-8 generations. The abundance of each tagged strain was measured with microarray at the beginning and at the end of the experiment. The fitness of the strain is the log2 ratio of these abundances. (This is not the same scale as fitness in population genetics.) The data is normalized so that the median strain has a fitness of 0. The fitness value of a gene is computed as the average of the values for the insertions in that gene. In this experiment it is assumed that the insertions of a given gene deactivate that gene. A study of MR-1 gene-phenotype relationships for 121 conditions has already been published.^122^ The CAGI challenge involved predicting results under eight more conditions.

Despite being offered in two successive rounds of CAGI, this challenge did not attract any submissions.

**MRN complex (CAGI3).** Genomes are subject to constant threat by damaging agents that generate DNA double-strand breaks (DSBs). The Mre11–Rad50–Nbs1 (MRN) complex plays important roles in detection and signaling of DSBs, as well as in the repair pathways of homologous recombination and non-homologous end-joining. The importance of Mre11-Rad50-Nbs1 complex in the cellular response to DNA double-strand breaks was initially revealed by ataxia telangiectasia-like disorder and Nijmgen breakage syndrome.

In this challenge, mutation screening of MRE11 and NBS1 genes was conducted from a series of approximately 1,300 breast cancer cases and 1,100 controls. There were 42 mutations listed for MRE11, and 44 mutations for NBS1, with more added during a short (one week) window in an optional second challenge (9 variants for MRE11, 1 for NBS1). Thirteen groups participated in the primary challenge, making a total of 23 submissions. Additional 17 submissions were made from 9 groups for the optional challenge. Assessment employed a logistic regression likelihood ratio test of the status of each subject (case/control) against the predicted probability of pathogenicity of the variant(s) that they carried. Predictions were also be assessed by calculated odds ratios and ROC areas.

Assessment revealed that method performance differed sharply on the two proteins, even though they were part of the same complex. Additionally, participants tended to not properly consider the likely distribution of neutral (p = 0.5) or protective (p < 0.5) mutations, which formed the majority in this challenge. Furthermore, a recent study for breast-cancer risk genes in over 113,000 women, revealed no significant association between NBS1 (also known as NBN) or MRE11 and breast cancer.^87^

**NPM-ALK (CAGI4).** Nucleoplasmin-anaplastic lymphoma kinase (NPM-ALK) is an oncogenic fusion protein found exclusively in a specific type of T-cell lymphoid malignancy, namely ALK-positive anaplastic large cell lymphoma (ALCL).^123^ Aberrantly activated NPM-ALK, specifically constitutive activation of the ALK tyrosine kinase, causes cell transformation through activation of several biological pathways related to cell proliferation, cell-cycle control and apoptosis. Small-molecule inhibitors of ALK are among the most promising drugs in several high-risk cancers, since ALK activation by mutation, amplification, or gene rearrangement is highly oncogenic. However, inhibitor efficacy can be hampered by several resistance mechanisms including point mutations in ALK.^124, 125^ An alternative approach involves inhibiting the molecular chaperone Hsp90, required for ALK folding, stability and/or activity.

In this experiment, NPM-ALK constructs with mutations in the kinase domain were assayed in extracts of transfected Hek-293T cells. ALK kinase activity was assessed by Western blotting using site-specific antibodies against phosphorylated ALK (Tyr1604) and STAT3 (Tyr705), one of ALK's downstream targets. Binding to Hsp-90 was assessed by immunoblotting and measured as the interaction density (band density) of each mutant relative to wildtype NPM-ALK. 23 variants (single amino acid, multiple amino acid substitutions and deletions) were assessed this way. The challenge involved predicting both the kinase activity and the Hsp90 binding affinity of the mutant proteins relative to the reference (wildtype) NPM-ALK fusion protein.

There were 4 submissions from 4 groups for this challenge. Assessment showed that predictors performed better than baseline tools, with the effect of short deletions being easier to predict than other mutation types.

**PGP (CAGI1-CAGI4).** A rather different class of challenge using large scale genome data used information from the Personal Genome Project (PGP). Participants in the project make their full sequence and phenotypic profile data publicly available. The four CAGI challenges were based on prerelease samples from this resource. The first two challenges, in 2010 and 2011, asked CAGI participants to predict which of 32 binary traits each individual has, given complete genome sequence. Using precision as the metric, results were quite poor, although for unclear reasons, the AUC values were much better, with a best AUC over 0.8. The second pair of challenges required matching each genome to a set of 239 self-reported binary phenotypes. Here results were slightly better than random (6 correct matches in the first round and 5 out of 23 in the second), but this is clearly a difficult task. Although the full 239 traits were available, participants seem to have gained most from a few strong genome/phenotype relationships, such ancestry, rare blood type and eye color.^73^ There were also some discrepancies observed between provided, self-reported traits and information from genomic data. Although the results are not the impressive, these challenges inspired one group to develop an interesting comprehensive Bayesian approach that may have broader application.

**RAD50 (CAGI2).** RAD50 is a component of the MRN (MRE11-NBS1-RAD50) complex, which plays a central role in double-strand break repair, DNA recombination, maintenance of telomere integrity and meiosis. RAD50 may be required to bind to DNA and hold the other two protein components of MRN in close proximity. Mutations in RAD50 are observed in a variety of cancers (stomach, intestinal, endometrial), and it is considered a candidate intermediate-risk breast cancer susceptibility gene. For this challenge, predictors are provided with a list of 69 RAD50 variants observed from sequencing RAD50 gene in about 1,400 breast cancer cases and 1,200 ethnically matched controls. These variants were observed between 1 and 20 times. The challenge is to predict the probability of the variant occurring in a case individual.

Eight groups participated in this challenge, submitting a total of 14 predictions. Assessment revealed no evidence in favor of pathogenicity from truncating variants, posing a problem for evaluating RAD50 pathogenicity and the quality of these predictions. However, limiting analysis to rare missense variants in the RAD50 DNA-binding domain (P-loop hydrolase and Zn hook) significantly improved predictor performance (AUROC for the top-performing methods increased by 20-25%), suggesting that incorporating gene-specific information could substantially improve results over typical methods that train on genome-wide mutations data. Furthermore, a recent study for breast-cancer risk genes in over 113,000 women, revealed no significant association between RAD50 and breast cancer.^87^

**riskSNPs (CAGI2, CAGI3).** Data from genome wide association studies (GWAS) are providing extensive information on the relationship between genetic variation and the risk of complex trait disease, such that there are now over 1000 reliable associations between the presence of SNPs at a particular locus and risk of a common disease (https://www.ebi.ac.uk/gwas/). Each association implies that a variant in that locus influences a molecular process affecting disease risk. The goal of these challenges is to investigate the community’s ability to identify underlying molecular mechanisms, given GWAS results. Since the correct mechanisms are unknown, there is no ranking of accuracy. In this sense, the challenge is different from the others in CAGI, aiming to assess the value of crowdsourcing in solving a pressing problem in data interpretation. Specifically, the goals are to ascertain which mechanisms appear most relevant, the degree of consensus between methods, and what fraction of loci can be assigned plausible mechanisms. Participants were provided with candidate SNPs for disease associated loci discovered in the Wellcome Trust Case Control Consortium (WTCCC1)^65^ and follow-up studies of seven complex diseases.

In all, SNPs in 178 loci were included in the CAGI2 challenge. Participants were asked to consider whether each candidate SNP might influence disease risk via any of the following mechanisms: missense SNPs (those that result in an amino acid substitution in a protein, thus potentially affecting *in vivo* function) - 4 predictor groups contributed using a total of 7 methods; expression (altering RNA level by a variety of possible mechanisms - two groups submitting), splicing (two groups submitting), and microRNA binding sites on messenger RNA (1 submission). In the second iteration of this challenge (CAGI3), 6 groups participated submitting a total of 13 predictors.

Missense methods are also used in a number of other CAGI challenges. Broadly, the methods use information on relative conservation of amino acid type at the substituted position, analysis of the effect of the substitution on protein structure and function, or a combination of both approaches. Methods for identifying expression effects either made use of information on known functional sites such as transcription factor binding positions or information from large scale studies of the association between the presence of SNPs and altered levels of expression. For splicing effects, one group restricted predictions to direct impact on splice junctions, yielding a very small number of possible mechanism SNPs. The second group used a more comprehensive approach, including possible enhancer sites and effects on cryptic splicing sites. The single microarray binding site method also makes use of database information.

There are two primary conclusions from this crowd-sourcing experiment. First, the results suggest both missense effects and changes in gene expression levels play a substantial role in molecular level mechanisms underlying these diseases. The exact extent is not yet clear, both because of limited expression predictions, and because the precise numbers depend on which set of SNPs are considered candidates. More data are also needed to assess the relative roles of splicing and microarray binding, as well as other factors. Although there is considerable variation in the missense results, a consensus view is encouraging. Consistency is not the same as accuracy, and the results suggest that large scale testing and benchmarking of missense analysis methods is needed to establish accuracy measures. Results for expression are intriguing in that the two methods used are based on different principles and produce rather different results.

**SCN5A (CAGI2).** Brugada syndrome (BrS) (OMIM #601144) is a rare clinical condition characterized by atypical right bundle branch block (RBBB) and elevated ST‐segments in right precordial leads in the absence of structural heart disease. Though most individuals with BrS are asymptomatic, the disease manifests at young age (20‐40 y) and men are more likely affected than women. Common symptoms are syncope and cardiac arrest or sudden death at rest or during sleep. BrS is inherited as an autosomal‐ dominant trait, with incomplete penetrance. Mutations in nine genes encoding ion channel subunits or gene products affecting ion channel function have been associated with BrS or proposed as risk factors (SCN5A, SCN1B, SCN3B, CACNA1C, CACNB2, KCND3, KCNE3, GPD1L, and MOG1). Mutations in SCN5A represent the majority with about 300 mutations in SCN5A linked to the syndrome. On a functional level, BrS mutations in SCN5A lead to loss of Na^+^ current through several mechanisms.

In this study, novel mutations in SCN5A were identified in two independent families with BrS and their effects on Nav1.5 channel function were investigated.

The mutant proteins were generated in the laboratory, heterologously expressed in CHO‐K1 cells and analyzed using the patch‐clamp technique. In these experiments, parameters such as current densities and channel kinetics (activation, inactivation, recovery from inactivation) were analyzed, comparing mutant channels to wildtype channels. Thus, the change induced by the mutant as a percentage change as compared to the wildtype channel was measured.

The challenge involved predicting the effect of 3 mutants on Nav1.5 function with respect to current densities, expressed as the percentage of current density reduction compared to the wildtype channel with a standard deviation. The predictions were assessed against the values obtained for each mutation in the patch‐clamp experiments.

Four groups participated in this challenge, submitting a total of 7 predictions. However, the dataset (3 variants) was too small to draw any significant conclusions regarding performance.

**SickKids clinical genomes (CAGI4, CAGI5).**  In the SickKids4 challenge, participants were provided full sequence data and phenotypic profiles in the form of Human Phenotype Ontology (HPO) terms^126^ for 25 undiagnosed patients from the SickKids Genome Clinic Project (https://www.sickkids.ca/), and asked to identify diagnostic variants and also to provide secondary findings – putative variants relevant to other diseases other than the reason for clinical presentation. Proposed rare disease causative mutations for two of the cases were deemed diagnostic by the referring physicians. This was the first instance of the CAGI community directly contributing in the clinic. Four groups participated in this challenge, submitting a total of 4 predictions. Prioritized variants were located in genes that had partial overlap with the clinical phenotype and were successfully validated. In two instances, the patient's referring clinical geneticist re-assessed the patient in light of the proposed disease gene and concluded that it was a good fit for the patient's phenotype. This was the first instance of CAGI participants providing a clinical diagnosis.^127^

SickKids5 contained WGS data and associated phenotypic profiles for 24 undiagnosed SickKids patients, and additionally required that participants match each genome to a patient phenotype list. This situation is artificially more difficult than encountered in the clinic but has the advantage of providing a clearer test of the methods – genome/phenotype matches above random must be due to correct identification of causative variants. Eight groups participated in this challenge, submitting a total of 9 predictions. No group did better than random in this assignment. However, two of the nominated diagnostic variants predicted with the highest probability for correct genome-patient matches, while not meeting ACMG criteria, were considered reasonable candidates for phenotype expansions, again potentially resolving previously intractable cases. In three of those cases, the referring physician accepted the proposed variants as causative, again resolving previously intractable cases. However, as seen in the other similar challenges, while these and other proposed variants may be correct, they are not supported by additional evidence as required by the ACMG/AMP guidelines for clinical assignment.^32^

**TP53 Splicing (CAGI3).** This challenge involved 3 TP53 splicing mutations implicated in cancer, assayed using minigene constructs to experimentally determine if each mutation influences splicing fidelity in HEK293T cells. The aim of the challenge was to predict the outcome of these experiments.

Five groups participated in this challenge, submitting a total of 5 predictions. The best-performing method, with an accuracy of 67%, used VEP (Variant Effect Predictor),^128^ followed by manual inspection.

**Warfarin exomes (CAGI4).** Warfarin is the most commonly prescribed anticoagulant for preventing thromboembolic events. Warfarin has a twenty-fold inter-individual dose variability and a narrow therapeutic index, and it is responsible for a third of adverse drug event hospitalizations in older Americans.^129^ Both clinical modifiers and genetic polymorphisms are known to affect an individual’s stable therapeutic warfarin dose.^130^ A dose estimator (IWPC) based on the status of SNPs in two genes as well as age, height, weight, race and two other prescribed drugs has been developed for Europeans; however, these algorithms are less predictive in diverse populations.^131^ In practice, physicians probably utilize a trial and adjust approach.

In this challenge, exome data and clinical covariates were provided for 53 African American individuals, with the aim to predict the therapeutic dose of warfarin. Exomes from an additional 50 African including warfarin doses were provided for use in training. Three groups participated with a total of 9 submissions. Results here were disappointing, with a maximum $R^{2}$ value of 0.25, compared with 0.35 obtained with the Caucasian standard IWPC method.^130^ The small sample size was likely a limiting factor in method performance.^29^

# Implementation Details

### Experimental-Max

When experimental assays use replicates, the uncertainty in the measurements can be quantified as the standard deviation of the replicates. The mean of the replicates is used as the experimental value, against which the predictions are evaluated. The uncertainty in the measurements poses an upper limit on the performance of the methods. To quantify this upper limit, we generate predictions reflecting the experimental uncertainty by sampling from a Gaussian distribution with the replicate mean and standard deviation as the parameters. Predictions are generated by this simulation 1000 times and the mean performance over these predictions is reported as the Experimental-Max performance. In the case where a method’s experimental value prediction is also used to create a score for the classification task, the Experimental-Max estimate of the classification performance measures is also computed. A summary of the estimates of a measure, in terms of mean, standard deviation, median, 5^th^ and 95^th^ percentile are stored, to be used in the figures and tables. In particular, the confidence intervals are derived from the 5^th^ and 95^th^ percentile and the AUC values in the figures are reported along with the 1.96 standard deviation.

### ROC from discrete class labels

In a few cases the methods submit a discrete class label instead of a continuous score. To plot the ROC curve in this case, we convert the predicted class labels to numeric scores: 0 (negative) and 1 (positive). The ROC curve is constructed from the numeric scores using the standard corrections for scores containing ties (see Handling ties in scores).

### log-log AUC

As argued in Methods, AUC, as a measure, is not calibrated appropriately for assessing a predictor’s performance in the clinical context. We use Truncated AUC as the main measure to this end; see Methods. Additionally, we use log-log AUC as another measure in the clinical context. Loosely, we define it as the area under the curve formed by plotting log TPR against log FPR. Intuitively, log-scale stretches the small values of TPR and FPR and consequently, enhancing the contribution of low TPR and FPR regions in the area computation. This is not to mean that a low TPR at a given FPR value contributes to improved log-log AUC. As one would expect, a higher TPR at a given value of FPR still contributes to a larger area. However, a small increase in TPR at low TPR values leads to a higher increase in the area compared to the same increase at high TPR values. Similarly, a small decrease in FPR at small FPR values leads to a higher increase in the area compared to the same decrease at high FPR values.

There are some difficulties in practically computing log-log AUC that stem from the fact that log function maps the interval, $[0,1]$, the range of TPR and FPR, to an unbounded interval $(-\infty, 0].$ This would lead to an infinite area under the log-scaled curve, unless the TPR and FPR values are both bounded above 0. We bound the TPR and FPR values around the lowest positive values that they can take on a given dataset. For a dataset with $n_{+}$ positives and $n_{-}$ negatives, $1/n_{+}$and $1/n_{-}$ are the smallest positive values that TPR and FPR can take at any given threshold. We restrict the range of log TPR and log FPR to $\log1/n_{+}$ and $\log1/n_{-}-\log2$, respectively; i.e., if TPR at a given threshold is $0$, then log TPR is assigned the value $\log1/n_{+}$ and similarly, if FPR at a given threshold is $0$, then log FPR is assigned the value $\log1/n_{-}-\log2$. The factor of $\log2$ is subtracted for computing the minimum log FPR to account for the area under the standard ROC curve between FPR values of 0 and $1/n_{-}$, which is non-zero when a positive TPR is achieved at 0 FPR. When converted to log-scale this region has infinite length. Instead of completely removing this region from the area computation, we bound this region to a length of $\log2$ to the left of $\log1/n_{-}.$ The choice $\log2$ comes from the distance between the smallest positive value of FPR and the second smallest positive value of FPR ($2/n_{-})$ on a log-scale; i.e., $\log2=\log2/n_{-}-\log1/n_{-}$.

Note that to correctly compute the area under the log-scaled curve, we use log TPR = $\log1/n_{+}$ as the reference line, instead of log TPR = 0; in other words, the area is computed between the log-scaled curve and the line log TPR = $\log1/n_{+}$. In order to ensure that the measure has a range of $\left[ 0,1 \right]$ (similar to the standard AUC), we normalize the area thus computed by dividing by the maximum area for the perfect classifier. The maximum area is given by the $\left( \log n_{+} \right)\times\left( \log{2n}_{-} \right)=\left( \log1- log 1/n_{+} \right)\times\left( \log1-(log {1/n}_{-}-\log2) \right).$ For convenience, we use the log function with base 10. For example, this allows us to convert the log TPR value of -2 as a TPR value of 0.01.

Unlike AUC and truncated AUC, the log-log AUC for the random classifier is not a constant. It is a function of the number of positives and negatives in the dataset. The width of the log TPR and log FPR axes is $\log n_{+}$ and $\log{2n}_{-}$, respectively. If $n_{+}>2n_{-}$, the log-log ROC curve of a random classifier intersects the log TPR axis. In this case the unnormalized log-log AUC is ${0.5\left( \log2n_{-} \right)}^{2}+ \log2n_{-}\left( \log n_{+}-\log2n_{-} \right).$ After normalization it is $1-0.5(log 2n_{-})/(log n_{+})$, which evaluates to a value above 0.5. However, if $n_{+}<2n_{-}$, the log-log ROC curve of a random classifier intersects the log FPR axis. In this case the unnormalized log-log AUC is ${0.5\left( \log n_{+} \right)}^{2}.$ After normalization it is $0.5(log n_{+})/(\log{2n}_{-}),$which evaluates to a value below 0.5. In the case when $n_{+}=2n_{-}$, the log-log AUC of the random classifier is the same as that standard AUC, i.e., 0.5.

In comparison to Truncated AUC, log-log AUC is more aggressive in enhancing the importance of very small FPR and TPR range. Furthermore, the calibration of the log scaled curve is dependent on the number of positives and negatives in the dataset. Smaller FPR and TPR ranges get further enhanced with a larger dataset size. The appropriate level of calibration of the ROC curve in the clinical context requires further research. Potential approaches include using a power function with fractional power such as square root, cube root or the fourth root to scale FPR and TPR.

### Bootstrap

To estimate the variability of the performance measures, 1000 bootstrap samples were generated from the dataset. For all the classification and clinical measures, the positive and negative variants were resampled separately and then combined to create a single bootstrap sample. A summary of the bootstrap estimates of the measures in terms of mean, standard deviation, median, 5^th^ and 95^th^ percentile are stored, to be used in the figures an tables. In particular, the confidence intervals are derived from the 5^th^ and 95^th^ percentile and the AUC values in the figures are reported along with the 1.96 standard deviation.

## Handling infinity and indeterminate values

Some of the classification score dependent measures, considered in our analysis can take infinite value in theory and when computed on real data (e.g., LR^+^, LR^-^, DOR, local lr^+^). To obtain finite bootstrap summaries, we replace the (positive) infinite values by 1000, as a conservative approximation. If a finite value greater than 1000 was achieved by the measure on any other score, the infinite values of the measure are replaced by the maximum finite value, instead of 1000.

Furthermore, measures such as LR^-^ and DOR when computed from real data achieve indeterminate value of 0/0 at the smallest score. This is because TPR and FPR at the smallest score are both equal to 1 and consequently, FNR and TNR are both equal to 0. Since the ratio FNR/TNR appears in the formulation of LR^-^ and DOR, the two measures are indeterminate at the smallest score. In this case, we replace the indeterminate value at the smallest score by the value of the measure computed at the second smallest score (strictly smaller than the smallest score) to enforce continuity.

## Handling ties in scores

Ties in scores, predictions and observed experimental values need to be handled explicitly, while computing some measures. This includes Spearman’s correlation, Kendall’s tau, TPR, FPR, posterior probability of pathogenicity $(\rho)$, local lr^+^, RR and other measures that are derived from these measures. Spearman’s correlation is defined as the linear correlation between the observed and predicted values’ ranks. As per the standard approach to handle ties, the initial ranks of the tied scores are modified before calculating the correlation. The modified rank of a given set of tied predictions (or observations), is obtained by averaging their original ranks. The standard correction of Kendall’s tau for ties is given by Kendall’s tau-b (formula in Methods). An efficient algorithm ($O(n\log n)$) to compute Kendall’s tau-b was implemented.^79^

As per the standard approach, post-correction TPR (FPR) at a tied score value, is given by the maximum of the pre-correction TPR (FPR) values computed at all data points tied at that score. The pre-correction TPR (FPR) value at a data point is obtained by first ordering all data points in the descending order of its score value and then counting the number of positive (negative) labels encountered traversing from left to right on reaching the given data point and dividing by the total number of positive (negative) labels in the dataset. Correcting TPR and FPR for ties, gives the corrected ROC curve. The AUC, LR^+^, LR^-^, DOR, PPP, PPV and MCC are computed from the corrected TPR and FPR.

Computation of local lr^+^, class prior adjusted posterior and RR, relies on the computation of the unadjusted posterior reflecting the data prior (see Methods). Consequently, correcting the unadjusted posterior for ties also corrects these measures. The unadjusted posterior at a score value is computed as the average class label (proportion of positive labeled points), where 1 and 0 are the positive and negative class labels, respectively, lying within a window around the given score. To correct for ties, the class labels for a set of data point with equal scores are replaced by a soft class label equal to the average class label in the set. Using the new class labels to compute the unadjusted posterior corrects for ties. Note that the correction is only required if a window around a score can potentially contain a non-trivial subset of a set of tied scores, which is indeed the case when a minimum number of data points, in addition to window width, is used to construct the window.

# Supplementary Figures

The evaluation for each challenge is contained in one or more sheets in Additional files 2-7: Tables S2-S7; see Supplementary Tables and Analyzed Challenges. A summary of each sheet is visually presented as a column of plots in the figures below and in the Main file. A description of the contents of each plot type is given below. In the following text, we use the term “selected methods” to refer to a subset of methods used to summarize the performance achieved on a given challenge. Loosely, the selected methods are obtained by first ranking each submitted method based on one or more measures from (Pearson’s correlation, Kendall’s Tau, AUC and Truncated AUC), depending on the type of analyses applicable to the challenge. Then each method’s final rank is calculated as its average rank on the applicable measures; see Supplementary Tables. The selected methods are picked as the top two methods based on the final ranking. In case of Annotate all Missense challenge figures, four selected methods are picked: two from the meta predictors and two from the non-meta predictors. The top-ranking method, among the selected methods is referred to as the “primary selected method” and the others are collectively referred to as the “secondary selected methods”.

The plots displayed for a challenge depend on the type of analysis applicable to the challenge; see Supplementary Tables and Analyzed Challenges. The confidence intervals and the 1.96 standard deviations displayed in the figures are computed with 1000 bootstrap samples. The prior used for the clinical analysis, corresponds to the data prior (see Supplementary Tables) for all the Biochemical effect challenges. In case of Annotate all Missense, figures are displayed with two priors: 0.1 and 0.01 corresponding to the diagnostic and screening setting, respectively.

**Scatter plot:** The scatter plot is displayed for any challenge with regression analysis. It displays the continuous experimental values (y-axis) measured in a challenge against the predicted values (x-axis) by the primary selected method. A light grey, solid perfect prediction line, $x=y$, is drawn for an easy comparison. If a classification type analysis is also applicable to a challenge and the ground truth class labels are defined by a class boundary separating the experimental measurements, the class boundary is displayed as a horizontal grey dashed line. The points on either side of the line correspond to the positive (purple) and negative (yellow) class. The scatter plot may include points (grey) not included in the classification analysis. To show how the predictions would separate the positives from the negatives a vertical grey dashed line is also displayed at the class boundary. Note that, though it is natural to use the class boundary as a threshold for the predictions, it is not the only possible threshold. When performing a clinical analysis, the evidence thresholds are more relevant. In all challenges with a clinical analysis, we display the reachable evidence thresholds and their 90% confidence interval below the scatter plot. Since the evidence thresholds are computed w.r.t. a class prior, the prior is also displayed.

**Correlation bar plot:** A correlation bar plot is displayed for any challenge with a regression analysis. Kendall’s tau and Pearson’s correlation is displayed for all selected methods and, if available, the baseline method and the Experimental-Max. 90% confidence interval are displayed for the correlations. The correspondence between the bar colors and the methods is given in the ROC curve legend.

**ROC plots:** An ROC plot is displayed for any challenge with classification analysis. ROC curves are displayed for all selected methods and, if available, the baseline method and the Experimental-Max. The corresponding AUC value along with 1.96 standard deviation is displayed in the legend. If clinical analysis is applicable to the challenge, Truncated ROC curves (see Methods) and log-log ROC curves (see Implementation Details) are also displayed in two separate plots. The corresponding Truncated AUCs and the log-log AUCs, along with 1.96 standard deviation, are also given in the legend. The dashed grey line in all ROC plots corresponds to the random classifier. For the primary selected method, points corresponding to the (FPR, TPR) values at the evidence thresholds, reached by the method, are displayed on the Truncated ROC curve and are annotated as Sup(porting), Mod(erate) or Str(ong). Since the evidence thresholds, and consequently the (FPR TPR) values they achieve, are computed w.r.t. a class prior, the class prior is also displayed in the plot.

**Posterior and** $\mathbf{l}\mathbf{r}^{\mathbf{+}}$**plot:** For all the challenges with a clinical analysis, a plot with posterior probability of pathogenicity (red) and the local $lr^{+}$(blue) curves of the primary selected method is displayed. If a clinical analysis in not applicable, but classification is, only the $lr^{+}$ curve is displayed. Both curves are plotted as a function of the predictions by the method. A smoothed version of the curves, derived by fitting a neural network with two hidden neurons, is also provided in cases where the original curve is jagged. For all the biochemical effect challenges, the posterior curve is computed w.r.t. the data prior; see Supplementary Table. In case of Annotate all Missense, two posterior curves corresponding to the diagnostic and screening priors are displayed; see Methods. To prevent the figure from being too crowded the $\mathrm{lr}^{+}$ curve is displayed on a separate plot in that case. For the Biochemical challenges, where the two curves are displayed in the same plot, the posterior values are read w.r.t. the left axis and the $\mathrm{lr}^{+}$ values, w.r.t to the right axis. The reached evidence thresholds along with their confidence intervals are displayed on the posterior curve. The posterior value at which an evidence threshold is displayed gives the minimum value of the posterior required to achieve that level of evidence. The $\mathrm{lr}^{+}$value where a vertical line drawn from an evidence threshold intersects the$\mathrm{lr}^{+}$curve gives the minimum value of $\mathrm{lr}^{+}$ required to achieve that level of evidence. For most Biochemical challenges the posterior and $lr^{+}$curves increase in the left direction because, in those cases, low prediction scores correspond to the positive class. For other challenges, with curves increasing to the right, high prediction scores correspond to the positive class. This has implication on how the evidence threshold should be interpreted. When the curves are increasing to the left the all the variants having a prediction below an evidence threshold meet that level of evidence. Whereas if the curves increase to the right the variants with a prediction above an evidence threshold meet that level of evidence. The percentage listed next to an evidence threshold is the PPP value at that threshold computed w.r.t. the same prior as that used for computing the threshold and the posterior; see Methods. This value gives the percent of variants reaching that evidence level, assuming the prior used in the computation.


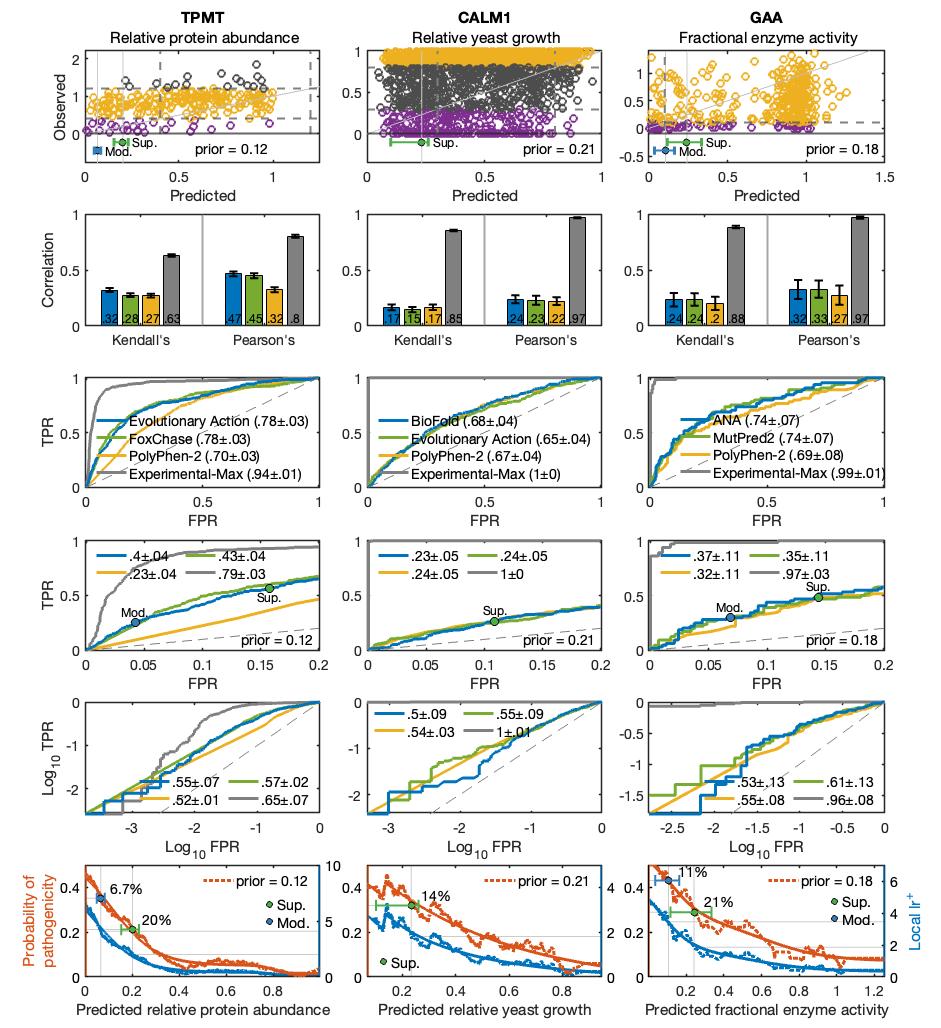
**Figure S1:** Summary of evaluation for TPMT (left), CALM1 (middle) and GAA (right) sheets in Additional file 2: Table S2. The selected methods are picked based on Pearson’s correlation, Kendall’s Tau, AUC and Truncated AUC. (1) Row 1 contains the scatter plots of the experimental values versus their predictions by the primary selected method. The horizontal grey dashed line is the class boundary separating the experimental values into positives (purple) and negatives (yellow). In case of CALM1 there are two class boundaries to additionally separate the neutrals; see Analyzed challenges. A vertical grey dashed line is also drawn at the class boundary. A solid, light grey perfect prediction line, $x=y$, is drawn for easy comparison. Below the scatter plot, the thresholds for the clinical evidence levels, reachable by the primary selected method, along with their 90% confidence intervals are displayed. The prior used to determine the thresholds is also displayed. (2) Row 2 displays Kendall’s $\tau$ and Pearson’s correlation for the selected methods (primary: blue, secondary: green), PolyPhen-2 and Experimental-Max. (3-5) Rows 3, 4 and 5 contain the ROC, Truncated ROC, and log-log ROC, respectively, for the selected methods, PolyPhen-2, Experimental-Max and the random classifier (dashed, grey line). The corresponding AUC, Truncated AUC and log-log AUC values, along with their 1.96 standard deviation, are also displayed. For the primary selected method (blue), the FPR, TPR values corresponding to the reachable clinical evidence thresholds are displayed as points on its Truncated ROC curve. The corresponding class prior is also displayed. (6) Row 6 contains the posterior probability of pathogenicity (red) and the local $lr^{+}$ (blue) curve of the primary selected method. A smoothed version of both curves is also displayed. The posterior curve is read w.r.t. the left y-axis, whereas $lr^{+}$ is read w.r.t. the right y-axis. The reachable evidence thresholds and their 90% confidence intervals are displayed on the posterior curve. The PPP value at an evidence threshold, giving the proportion of variants satisfying the threshold, is displayed as a percentage. The prior used to compute the posterior curve, evidence thresholds and PPP is also displayed.


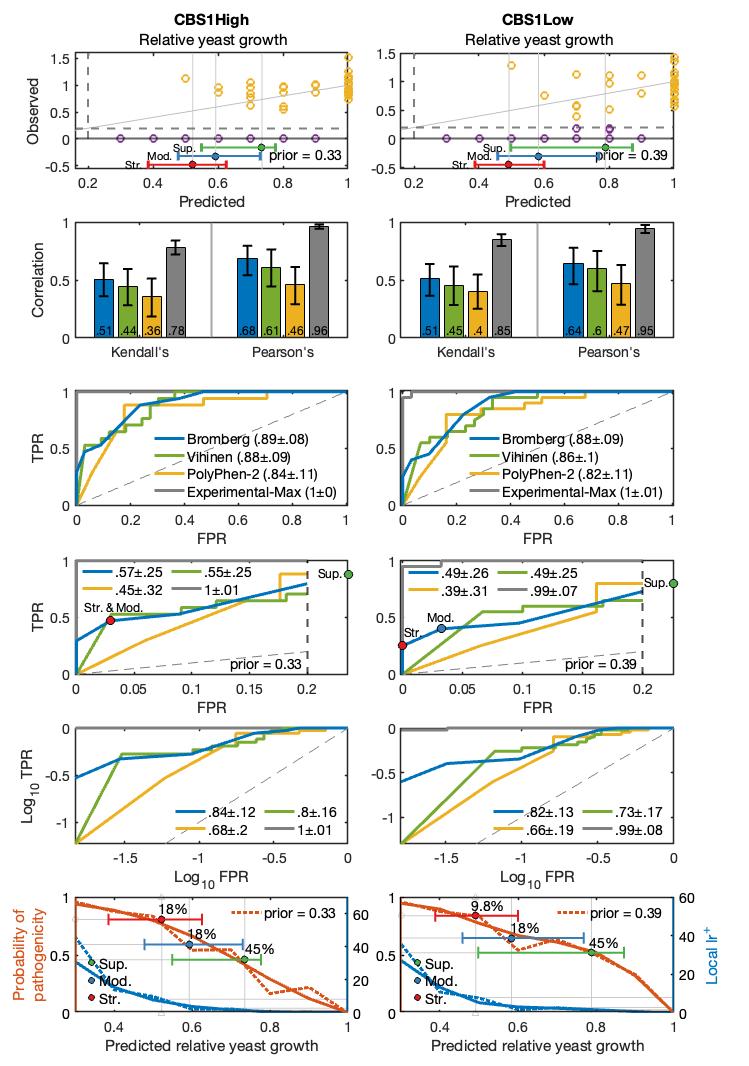


**Figure S2A:** Summary of evaluation for CBS1High (left) and CBS1Low (right) sheets in Additional file 2: Table S2 for the CAGI 1 CBS challenge. The selected methods are picked based on Pearson’s correlation, Kendall’s Tau, AUC and Truncated AUC. (1) Row 1 contains the scatter plots of the experimental values versus their predictions by the primary selected method. The horizontal grey dashed line is the class boundary separating the experimental values into positives (purple) and negatives (yellow). A vertical grey dashed line is also drawn at the class boundary. A solid, light grey perfect prediction line, $x=y$, is drawn for easy comparison. Below the scatter plot, the thresholds for the clinical evidence levels, reachable by the primary selected method, along with their 90% confidence intervals are displayed. The prior used to determine the thresholds is also displayed. (2) Row 2 displays Kendall’s $\tau$ and Pearson’s correlation for the selected methods (primary: blue, secondary: green), PolyPhen-2 and Experimental-Max. (3-5) Rows 3, 4 and 5 contain the ROC, Truncated ROC, and log-log ROC, respectively, for the selected methods, PolyPhen-2, Experimental-Max and the random classifier (dashed, grey line). The corresponding AUC, Truncated AUC and log-log AUC values, along with their 1.96 standard deviation, are also displayed. For the primary selected method (blue), the FPR, TPR values at the reachable clinical evidence thresholds are displayed as points on its Truncated ROC curve. The corresponding class prior is also displayed. (6) Row 6 contains the posterior probability of pathogenicity (red) and the local $lr^{+}$ (blue) curve of the primary selected method. A smoothed version of both curves is also displayed. The posterior curve is read w.r.t. the left y-axis, whereas $lr^{+}$ is read w.r.t. the right y-axis. The reachable evidence thresholds and their 90% confidence intervals are displayed on the posterior curve. The PPP value at an evidence threshold, giving the proportion of variants satisfying the threshold, is displayed as a percentage. The prior used to compute the posterior curve, evidence thresholds and PPP is also displayed.


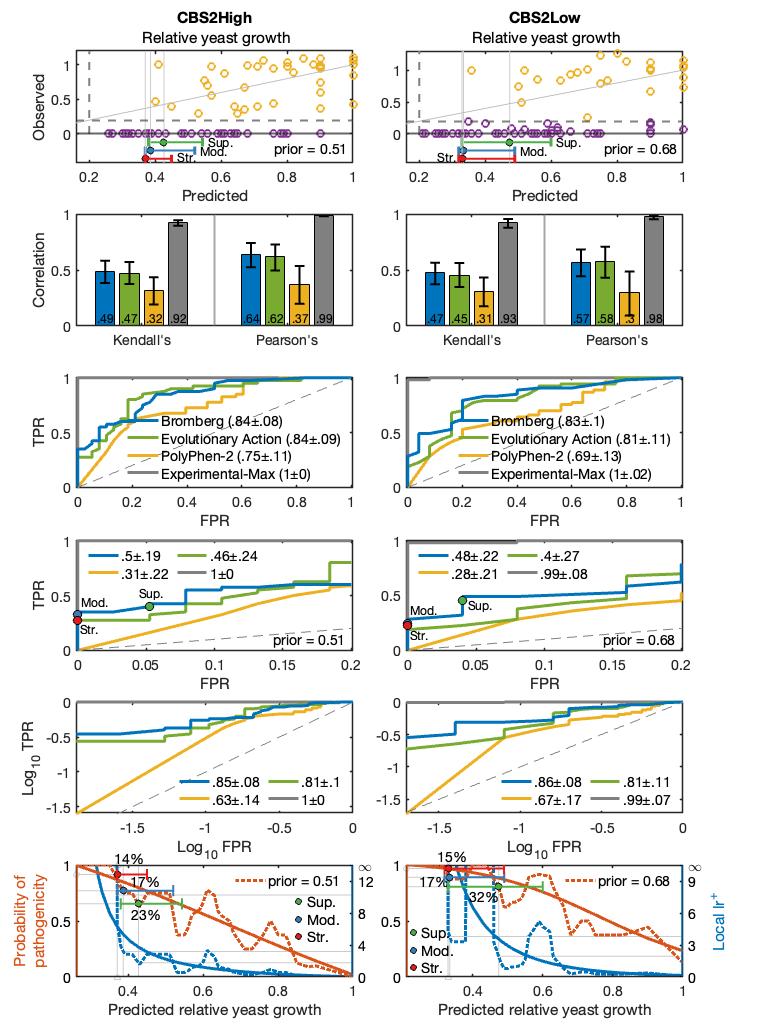


**Figure S2B:** Summary of evaluation for CBS2High (left) and CBS2Low (right) sheets in Additional file 2: Table S2 for the CAGI 2 CBS challenge. The selected methods are picked based on Pearson’s correlation, Kendall’s Tau, AUC and Truncated AUC. (1) Row 1 contains the scatter plots of the experimental values versus their predictions by the primary selected method. The horizontal grey dashed line is the class boundary separating the experimental values into positives (purple) and negatives (yellow). A vertical grey dashed line is also drawn at the class boundary. A solid, light grey perfect prediction line, $x=y$, is drawn for easy comparison. Below the scatter plot, the thresholds for the clinical evidence levels, reachable by the primary selected method, along with their 90% confidence intervals are displayed. The prior used to determine the thresholds is also displayed. (2) Row 2 displays Kendall’s $\tau$ and Pearson’s correlation for the selected methods (primary: blue, secondary: green), PolyPhen-2 and Experimental-Max. (3-5) Rows 3, 4 and 5 contain the ROC, Truncated ROC, and log-log ROC, respectively, for the selected methods, PolyPhen-2, Experimental-Max and the random classifier (dashed, grey line). The corresponding AUC, Truncated AUC and log-log AUC values, along with their 1.96 standard deviation, are also displayed. For the primary selected method (blue), the FPR, TPR values at the reachable clinical evidence thresholds are displayed as points on its Truncated ROC curve. The corresponding class prior is also displayed. (6) Row 6 contains the posterior probability of pathogenicity (red) and the local $lr^{+}$ (blue) curve of the primary selected method. A smoothed version of both curves is also displayed. The posterior curve is read w.r.t. the left y-axis, whereas $lr^{+}$ is read w.r.t. the right y-axis. The reachable evidence thresholds and their 90% confidence intervals are displayed on the posterior curve. The PPP value at an evidence threshold, giving the proportion of variants satisfying the threshold, is displayed as a percentage. The prior used to compute the posterior curve, evidence thresholds and PPP is also displayed.

**
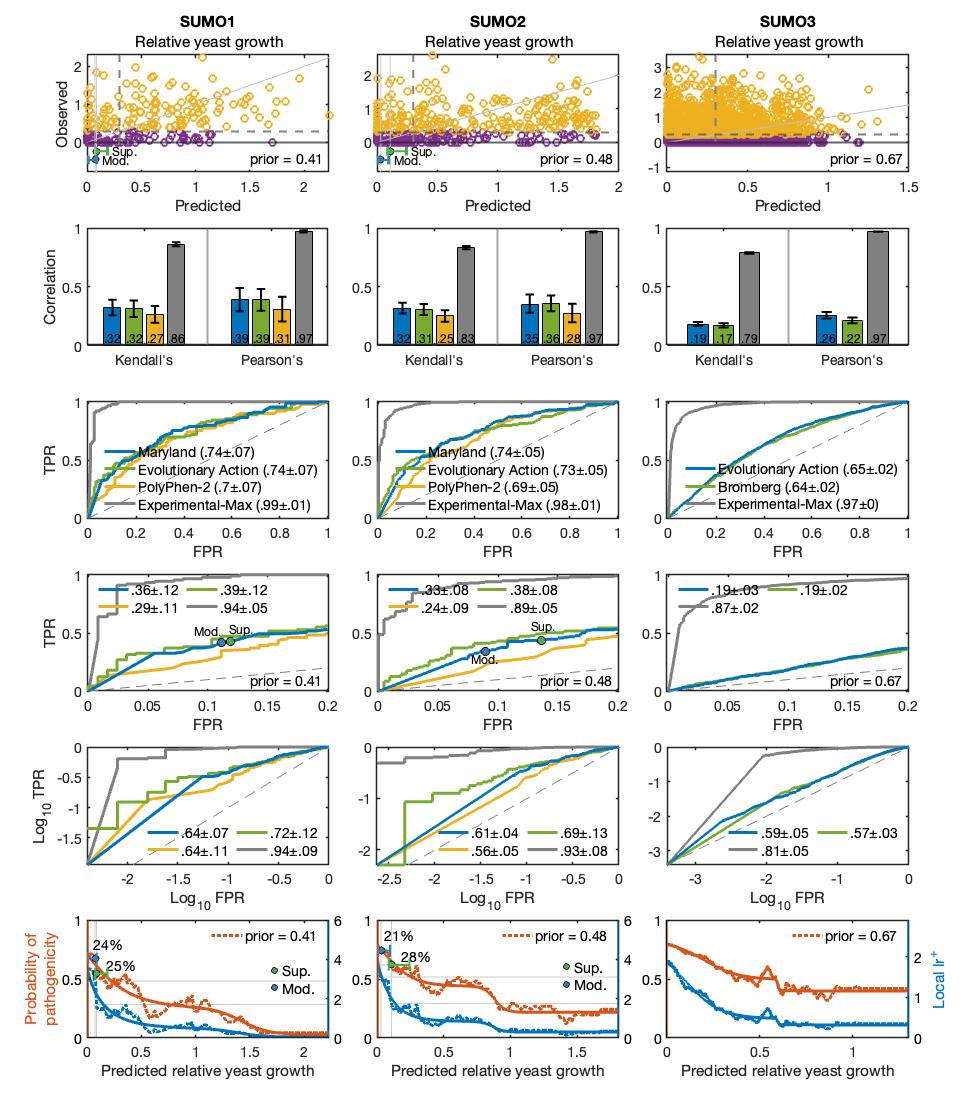
Figure S3:** Summary of evaluation for SUMO1 (left), SUMO2 (middle) and SUMO3 (right) sheets in Additional file 2: Table S2 for the three datasets in the SUMO challenge. The selected methods are picked based on Pearson’s correlation, Kendall’s Tau, AUC and Truncated AUC. (1) Row 1 contains the scatter plots of the experimental values versus their predictions by the primary selected method. The horizontal grey dashed line is the class boundary separating the experimental values into positives (purple) and negatives (yellow). A vertical grey dashed line is also drawn at the class boundary. A solid, light grey perfect prediction line, $x=y$, is drawn for easy comparison. Below the scatter plot, the thresholds for the clinical evidence levels, reachable by the primary selected method, along with their 90% confidence intervals are displayed. The prior used to determine the thresholds is also displayed. (2) Row 2 displays Kendall’s $\tau$ and Pearson’s correlation for the selected methods (primary: blue, secondary: green), PolyPhen-2 and Experimental-Max. (3-5) Rows 3, 4 and 5 contain the ROC, Truncated ROC, and log-log ROC, respectively, for the selected methods, PolyPhen-2, Experimental-Max and the random classifier (dashed, grey line). The corresponding AUC, Truncated AUC and log-log AUC values, along with their 1.96 standard deviation, are also displayed. For the primary selected method (blue), the FPR, TPR values at the reachable clinical evidence thresholds are displayed as points on its Truncated ROC curve. The corresponding class prior is also displayed. (6) Row 6 contains the posterior probability of pathogenicity (red) and the local $lr^{+}$ (blue) curve of the primary selected method. A smoothed version of both curves is also displayed. The posterior curve is read w.r.t. the left y-axis, whereas $lr^{+}$ is read w.r.t. the right y-axis. The reachable evidence thresholds and their 90% confidence intervals are displayed on the posterior curve. The PPP value at an evidence threshold, giving the proportion of variants satisfying the threshold, is displayed as a percentage. The prior used to compute the posterior curve, evidence thresholds and PPP is also displayed.


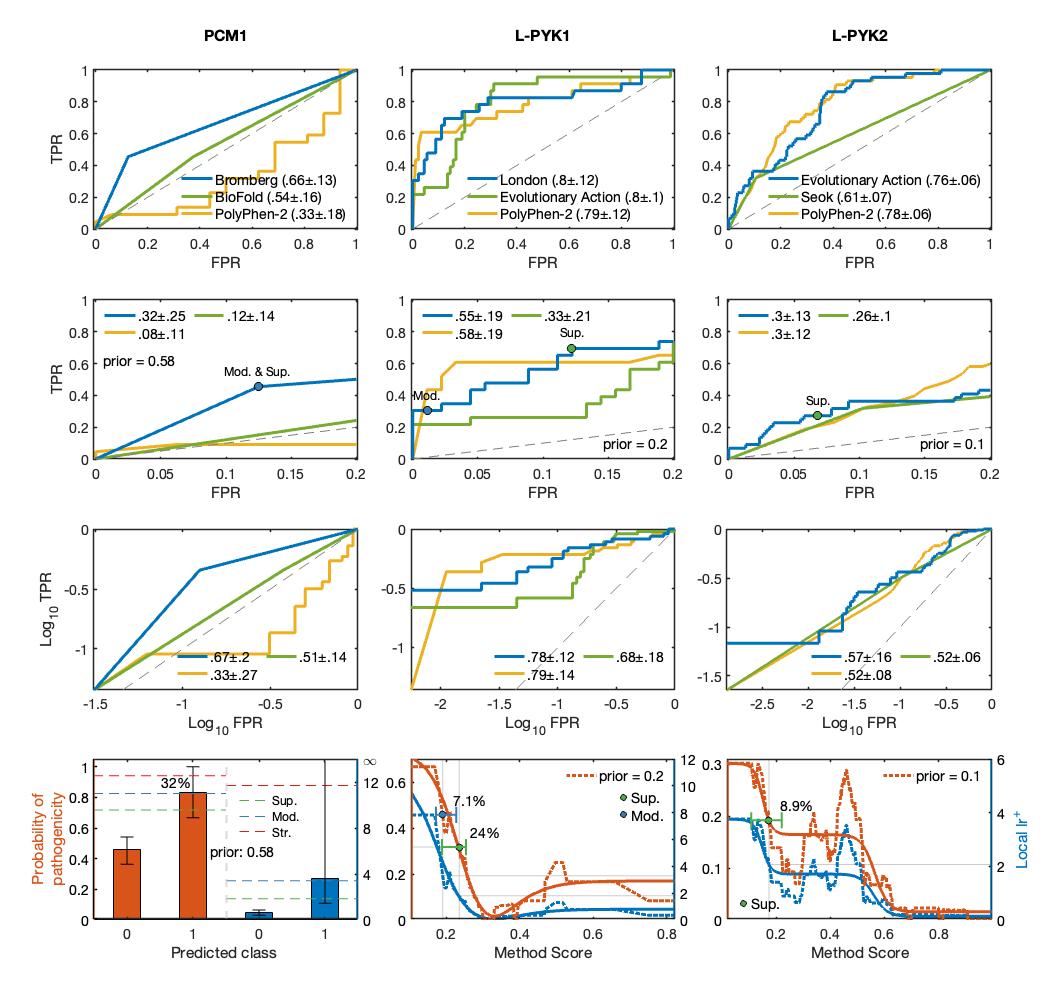


**Figure S4:** Summary of evaluation for PCM1 (left), LPYK1 (middle) and LPYK2 (right) sheets in Additional file 2: Table S2 for the PCM1 challenge and the two datasets from the L-PYK challenge. The selected methods are picked based on AUC and Truncated AUC. (1-3) Rows 1, 2 and 3 contain the ROC, Truncated ROC, and log-log ROC, respectively, for the selected methods (primary: blue, secondary: green), PolyPhen-2 and the random classifier (dashed, grey line). The corresponding AUC, Truncated AUC and log-log AUC values, along with their 1.96 standard deviation, are also displayed. For the primary selected method (blue), the FPR, TPR values at the reachable clinical evidence thresholds are displayed as points on its Truncated ROC curve. The corresponding class prior is also displayed. (4) For LPYK1 and LPYK2, row 4 contains the posterior probability of pathogenicity (red) and the local $lr^{+}$ (blue) curve of the primary selected method. A smoothed version of both curves is also displayed. Since continuous scores for the classification task in the PCM1 challenge were not available, the posterior and the $lr^{+}$ values are displayed as barplots at the predicted class labels. Note that the local $lr^{+}$ and$LR^{+}$ are the equivalent in this case. The posterior curve is read w.r.t. the left y-axis, whereas $lr^{+}$ is read w.r.t. the right y-axis. For LPYK1 and LPYK2, the reachable evidence thresholds and their 90% confidence intervals are displayed on the posterior curve. For PCM1, in absence of continuous scores, there are only two possible values for the evidence thresholds: 0 and 1. Consequently, a confidence interval for the evidence threshold does not make sense. To see the clinical relevance of the variants predicted to be positive, we display the posterior and $lr^{+}$ cutoffs for the supporting, moderate and strong evidence levels. The variants do attain posterior and $lr^{+}$ values above the respective cutoffs for supporting and moderate evidence. However, the 90% confidence intervals for the posterior and $lr^{+}$ indicates that the evidence levels might not always be attained. The PPP value at an evidence threshold, giving the proportion of variants satisfying the threshold, is displayed as a percentage in all the plots. The prior used to compute the posterior curve, evidence thresholds and PPP is also displayed.


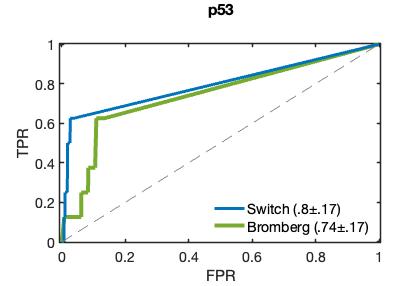


**Figure S5:** Summary of evaluation for p53 sheet in Additional file 2: Table S2. The selected methods are picked based on AUC. ROC curves for the selected methods (primary: blue, secondary: green) are displayed. The corresponding AUC values, along with their 1.96 standard deviation, are also displayed. No reasonable baselines were available as this challenge is about the classifying variants as rescue mutations. The local $lr^{+}$ curve and quantities from the clinical analysis were not reported because of small number of positives, which leads to a high variance in the binning-based estimates.


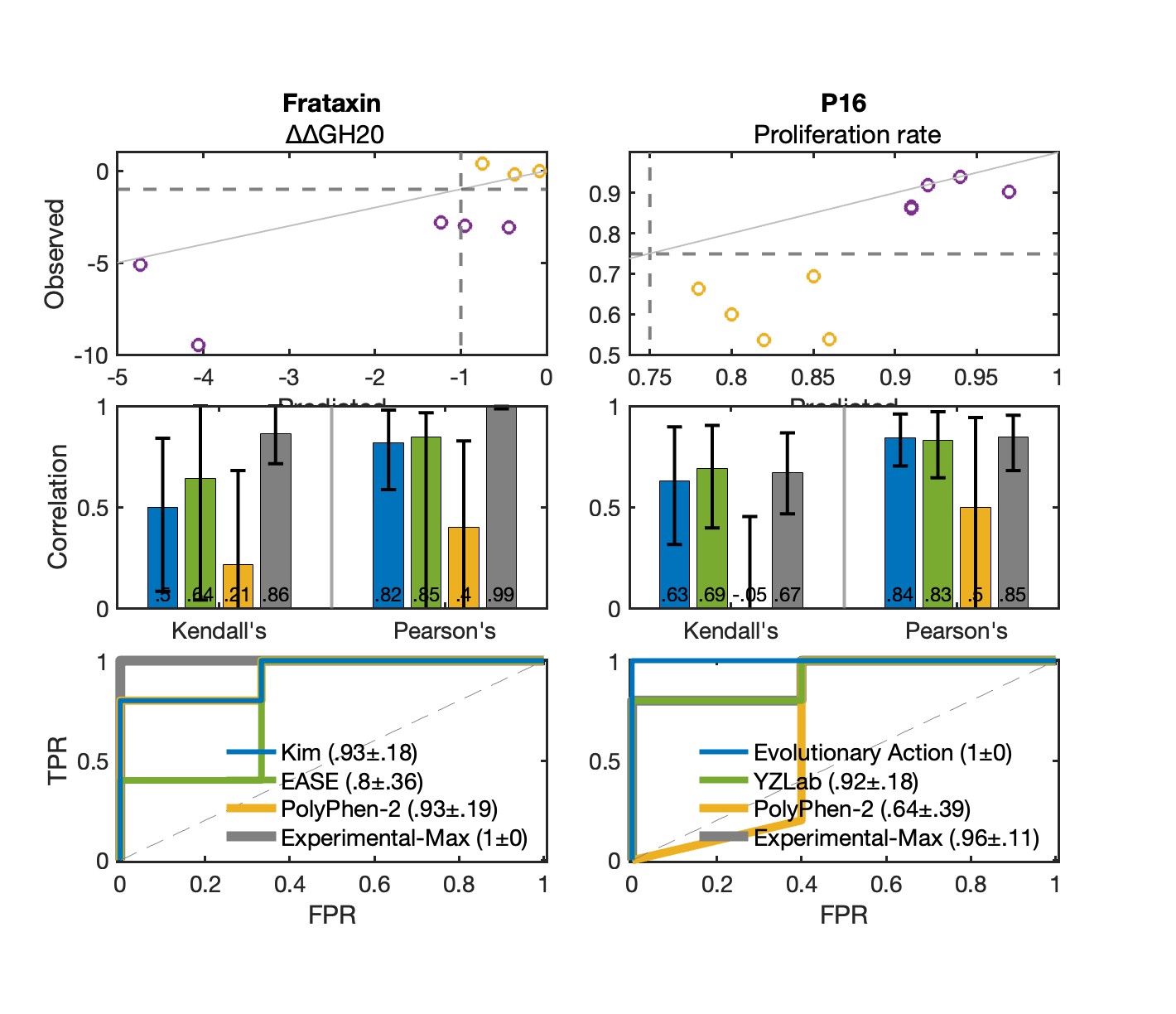
**Figure S6:** Summary of evaluation for Frataxin (left) and p16 (right) sheets in Additional file 2: Table S2. The selected methods are picked based on Pearson’s correlation, Kendall’s Tau and AUC. (1) Row 1 contains the scatter plots of the experimental values versus their predictions by the primary selected method. The horizontal grey dashed line is the class boundary separating the experimental values into positives (purple) and negatives (yellow). A vertical grey dashed line is also drawn at the class boundary. A solid, light grey perfect prediction line, $x=y$, is drawn for easy comparison. (2) Row 2 displays Kendall’s $\tau$ and Pearson’s correlation for the selected methods (primary: blue, secondary: green), PolyPhen-2 and Experimental-Max. (3) Row 3 contains the ROC curve for the selected methods, PolyPhen-2, Experimental-Max and the random classifier (dashed, grey line). The corresponding AUC values, along with their 1.96 standard deviation, are also displayed. The local $lr^{+}$ curve and quantities from the clinical analysis were not reported because of a small dataset size, which leads to a high variance in the binning-based estimates.


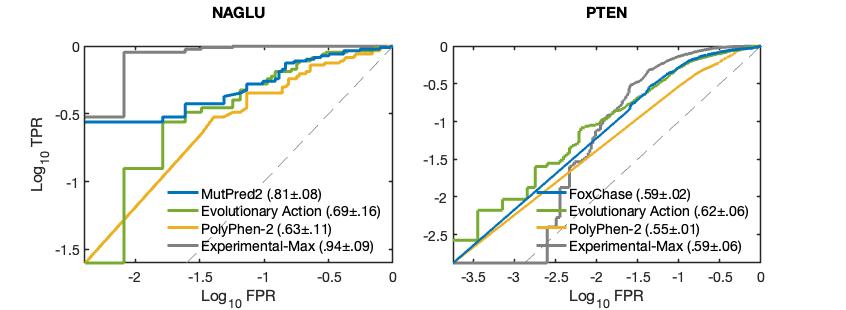


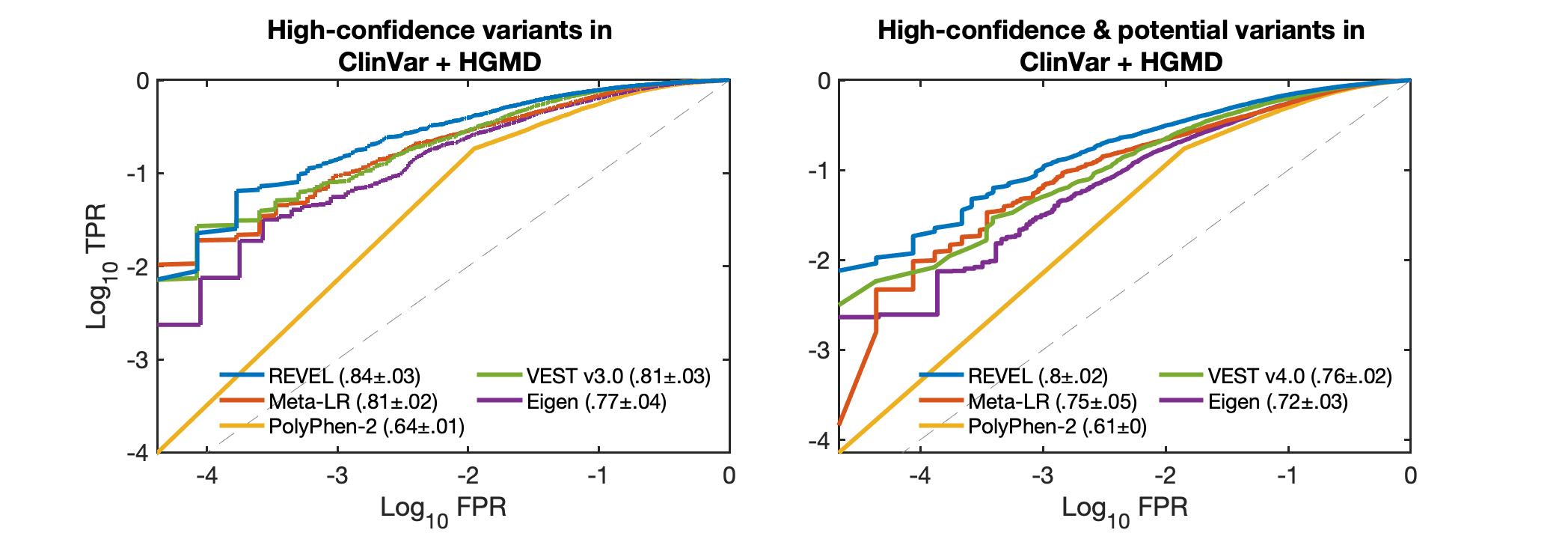


**Figure S7:** log-log ROC curves for NAGLU (top left) and PTEN (top right) sheets in Additional file 2: Table S2 and AAM1All (bottom left) and AAM2All (bottom right) sheets for the Annotate all Missense in Additional file 4: Table S4. For NAGLU and PTEN, the two selected methods are picked based on Pearson’s correlation, Kendall’s Tau and AUC and Truncated AUC. The plot displays log-log ROC for the selected methods (primary: blue, secondary: green), PolyPhen-2, Experimental-Max and the random classifier (dashed, grey line). For the Annotate all Missense sheets, the four selected methods (two meta and two non-meta predictors) are picked based on AUC and Truncated AUC. The plot displays log-log ROC for the four selected methods (primary: blue), PolyPhen-2 and the random classifier (dashed, grey line). The corresponding log-log AUC values, along with their 1.96 standard deviation, are also displayed. The remaining plots for NAGLU, PTEN, AAM1All and AAM2All are given in the Main file: Figures 2 and 3.

**
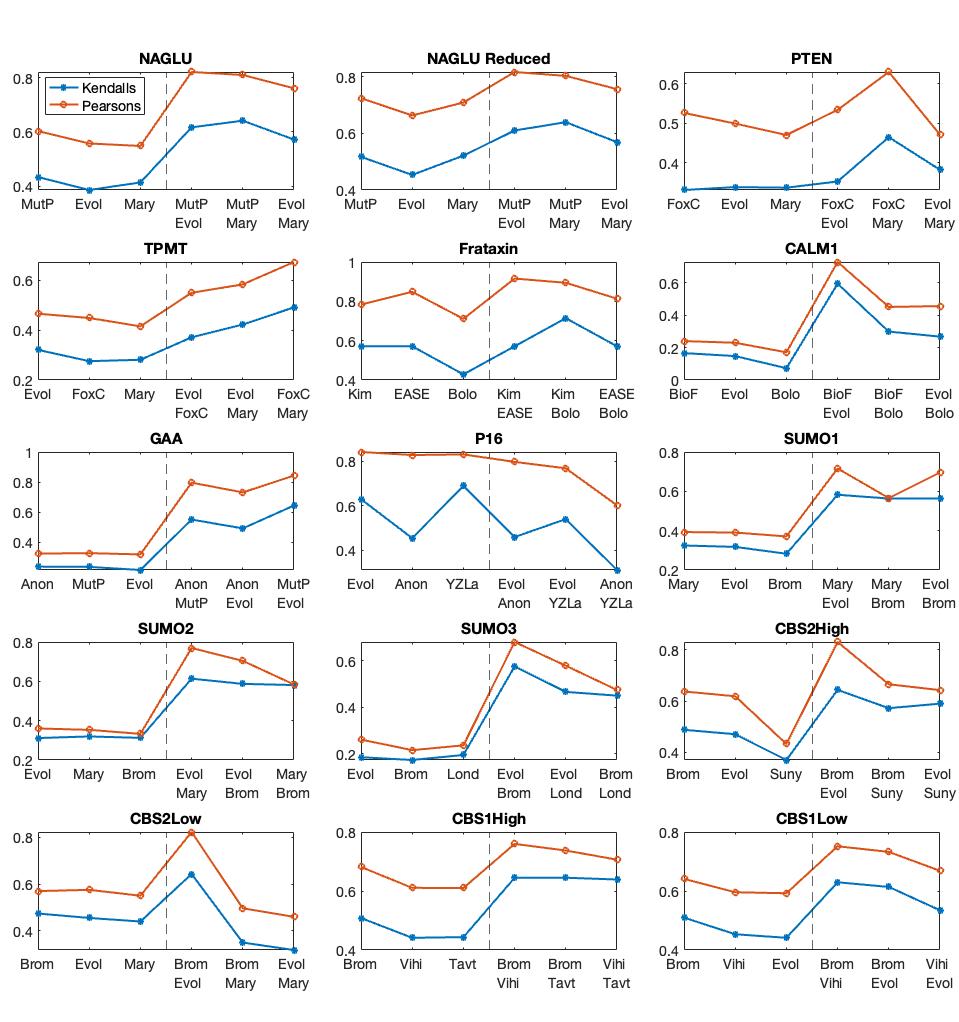
**

**Figure S8**: Comparison of prediction vs. experimental-value correlation and prediction vs. prediction correlation for the Biochemical challenge sheets with regression analysis in Additional file 2: Table S2. Three selected method are picked from each sheet; see Supplementary Tables. The first four letters of the method names are used for abbreviation. Each plot displays the prediction vs. experimental-value correlations (Pearson’s correlation and Kendall’s Tau) for the selected methods on the left of the grey, dashed line. The pairwise correlations between pairs of predictions from the selected methods is displayed to the right of the line. As a general trend, it seems that the predictions are more correlated amongst each other as compared to how they are correlated with the experimental value. Removing ten of the hard to predict variants from the 163 NAGLU variants significantly reduces the difference between correlations on the left and the right (2^nd^ plot). This suggests the difference between the two correlations is disproportionately due to a small number of variants.


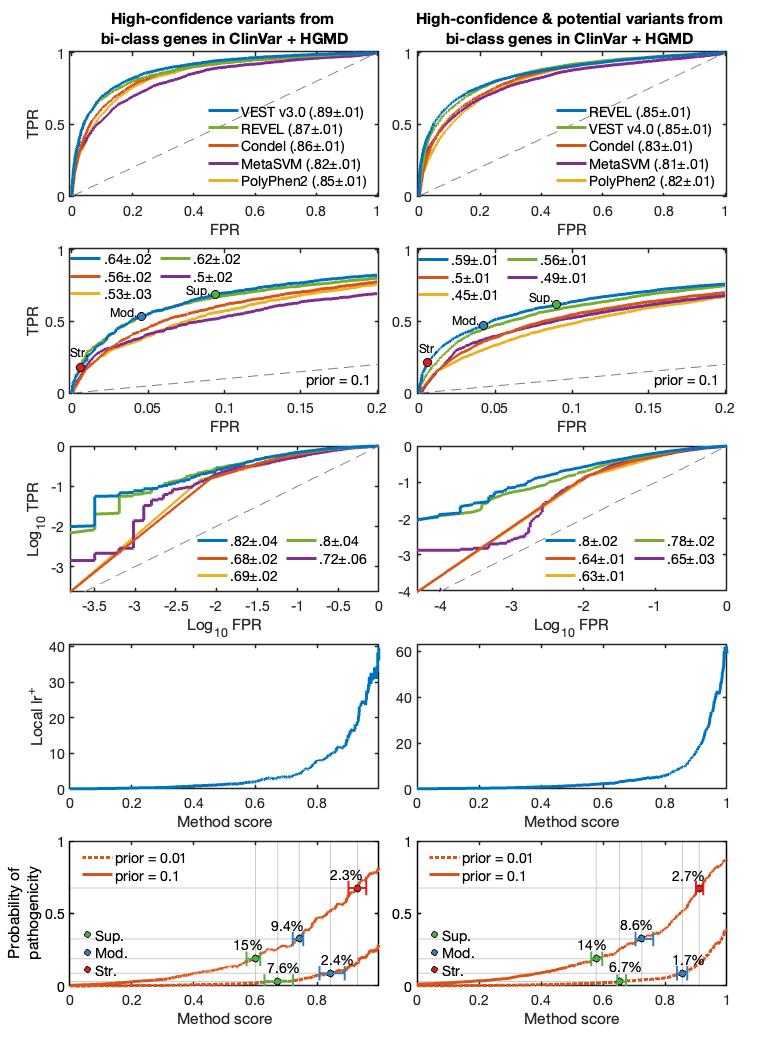


**Figure S9:** Summary of evaluation for AAM1BiClass (left), AAM2BiClass (right) sheets in Additional file 4: Table S4 for the Annotate all Missense challenge. The selected methods are picked based on AUC and Truncated AUC. (1-3) Rows 1, 2 and 3 contain the ROC, Truncated ROC, and log-log ROC, respectively, for the four selected methods (two meta-predictors and two non-meta predictors), PolyPhen-2 and the random classifier (dashed, grey line). The corresponding AUC, Truncated AUC and log-log AUC values, along with their 1.96 standard deviation, are also displayed. For the primary selected method (blue), the FPR, TPR values at the reachable clinical evidence thresholds are displayed as points on its Truncated ROC curve. The corresponding class prior is also displayed. (4) Row 4 contains the local $lr^{+}$curve of the primary selected method. (5) Row 5 contains the posterior probability of pathogenicity curves of the primary selected method at the screening (0.01) and diagnostic (0.1) prior. The reachable evidence thresholds and their 90% confidence intervals are displayed on the curves. The PPP value at an evidence threshold, giving the proportion of variants satisfying the threshold, is displayed as a percentage. The two priors used to compute the posterior curve, evidence thresholds and PPP are also displayed. Observe that the primary selected method on the confident set of variants is VEST3 whose ROC AUC decreased from 0.91 (Main file: Figure 3) to 0.88 as opposed to REVEL’s that decreased from 0.92 (Main file: Figure 3) to 0.87. In the second column, REVEL and VEST4 remained the primary selected methods on a larger set of confident and potential variants, with an identical ROC AUC (0.85), but with REVEL achieving 3 percentage points higher performance in the low false positive rate region (Truncated AUC). In terms of clinical performance, the test data differences translated to a lower $\mathrm{lr}^{+}$ at the extreme end of the prediction range and slightly more stringent thresholds, although there is almost no difference in the fraction of variants classified for Supporting, Moderate, or Strong evidential support (compare Main file: Figure 3). This suggests that the primary selected methods in this challenge are appropriately robust to be applied on a broad set of genes.


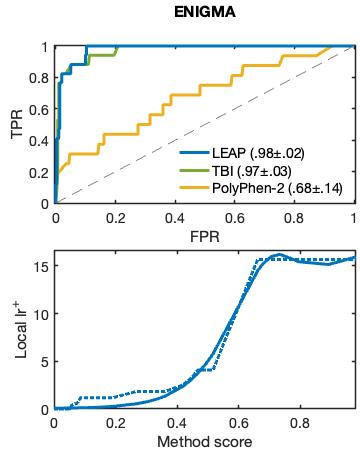


**Figure S10A:** Summary of evaluation for the ENIGMA sheet in Additional file 5: Table S5. The selected methods are picked based on AUC. (1) The top plot contains ROC curves for the selected methods (primary: blue, secondary: green), PolyPhen-2 and the random classifier (grey dashed line). The corresponding AUC values, along with their 1.96 standard deviation, are also displayed. (2) The bottom plot contains the local $lr^{+}$ curve of the primary selected method, with and without smoothing.

**
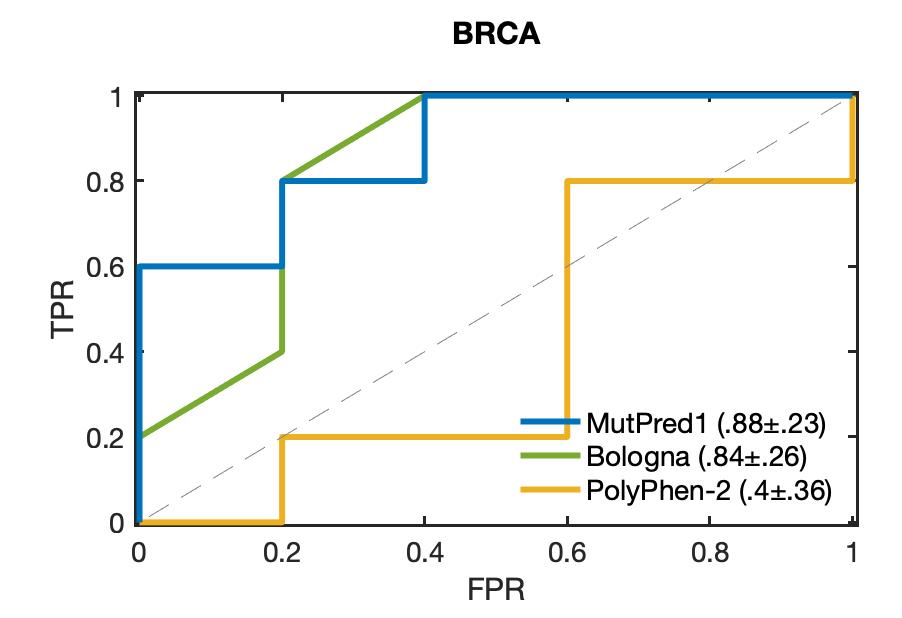
**

**Figure S10B:** Summary of evaluation for the BRCA sheet in Additional file 5: Table S5. The selected methods are picked based on AUC. ROC curves for the selected methods (primary: blue, secondary: green), PolyPhen-2 and the random classifier (grey dashed line) are displayed. The corresponding AUC values, along with their 1.96 standard deviation, are also displayed. The local $lr^{+}$ curve was not reported because of small number of positives, which leads to a high variance in the binning based estimates.

**
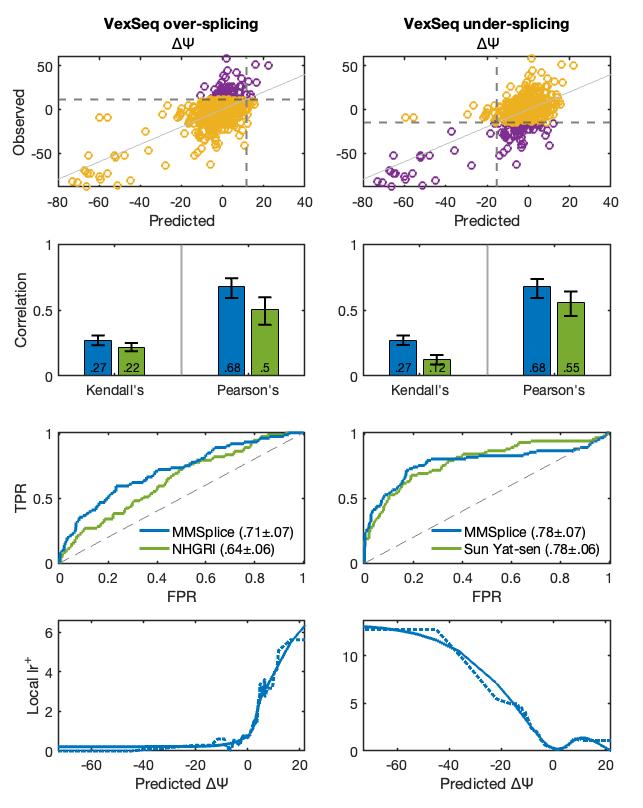
**

**Figure S11:** Summary of evaluation for VexSeq1 (left) and VexSeq2 (right) sheets in Additional file 6: Table S6 for the Vex-seq challenge. The selected methods are picked based on Pearson’s correlation, Kendall’s Tau, and AUC. (1) Row 1 contains the scatter plots of the experimental values versus their predictions by the primary selected method. The horizontal grey dashed line is the class boundary separating the experimental values into positives (purple) and negatives (yellow). A vertical grey dashed line is also drawn at the class boundary. A solid, light grey perfect prediction line, $x=y$, is drawn for easy comparison. (2) Row 2 displays Kendall’s $\tau$ and Pearson’s correlation for the selected methods (primary: blue, secondary: green). (3) Rows 3 contains the ROC for the selected methods and the random classifier (dashed, grey line). The corresponding AUC values, along with their 1.96 standard deviation, are also displayed. (4) Row 6 contains the local $lr^{+}$ curve of the primary selected method. A smoothed version of both curves is also displayed. In spite of decent Pearson’s correlation, the performance on predicting $\Delta\Psi$ is not very impressive as demonstrated by a relatively small Kendall’s $\tau.$ Pearson’s correlation being sensitive to outliers, achieves a high value due to the few points on the lower right. The classification performance is stronger for under-splicing, compared to over-splicing, with the top ROC AUC of 0.78 and a maximum lr+ of 13.

**
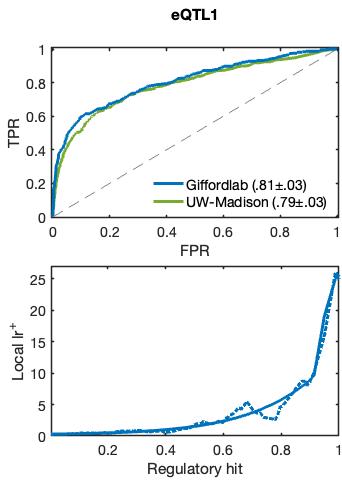
**

**Figure S12A:** Summary of evaluation for the eQTL1 sheet in Additional file 6: Table S6 corresponding to the first part of the eQTL challenge for predicting regulatory hits. The selected methods are picked based on AUC. (1) The top plot contains ROC curves for the selected methods (primary: blue, secondary: green) and the random classifier (grey dashed line). The corresponding AUC values, along with their 1.96 standard deviation, are also displayed. (2) The bottom plot contains the local $lr^{+}$ curve of the primary selected method, with and without smoothing. As the ROC curve and the local $lr^{+}$curve shows, the performance by the primary selected method is good.


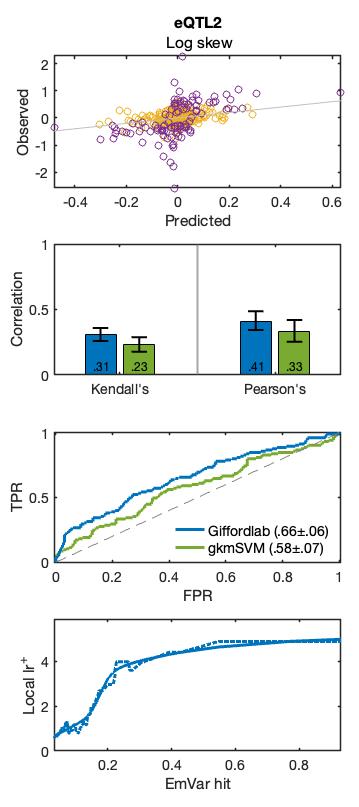


**Figure S12B:** Summary of evaluation for eQTL2 sheet in Additional file 6: Table S6 for the second part of the eQTL challenge. The selected methods are picked based on Pearson’s correlation, Kendall’s Tau, and AUC. (1) Row 1 contains the scatter plots of the experimental log_2_ allelic skew values versus their predictions by the primary selected method. The positives (*emVar*) and negatives are shown in purple and yellow, respectively. A solid, light grey perfect prediction line, $x=y$, is drawn for easy comparison. (2) Row 2 displays Kendall’s $\tau$ and Pearson’s correlation for the selected methods (primary: blue, secondary: green). (3) Rows 3 contains the ROC for the selected methods and the random classifier (dashed, grey line). The corresponding AUC values, along with their 1.96 standard deviation, are also displayed. (4) Row 6 contains the local $lr^{+}$ curve of the primary selected method. A smoothed version of both curves is also displayed. The performance in the log skew prediction task is modest (maximum Pearson’s corr.: 0.41, Kendall’s$\tau:0.31).$ The performance in the *emVar* classification task is poor (maximum ROC AUC is 0.66). Few variants produce greater than a 2-fold expression changes, and experimental uncertainty is often close to that, making it difficult to draw firm conclusions.

**Figure. S13***: Examples of structure-based explanations of variant impact.* (A) In the p16 challenge, the G23S substitution results in unfavorable electrostatic interactions.

B -D: Analysis of a p53 cancer driver rescue mutation. (B) In wildtype p53, R175 affects coordination of the zinc ion (grey sphere) and is mostly buried, with its head group making electrostatic interactions with the side-chain of D184 and the main-chain carbonyl of M237. (C) The M237I cancer driver mutation sterically interferes with R175, disrupting the conformation required for zinc coordination. Steric clashes are indicated by red disks. (D) A large-scale experimental scan for rescue mutations found that replacement of R175 with a small or medium sized amino acid (R175A, R175V, R175S, R175T, R175P) restored function in the presence of M237I. As the figure shows, these R175 mutations relieve the steric clashes. The top-performing method identified the rescue mutations, using a combination of sequence conservation and stability analysis.

**
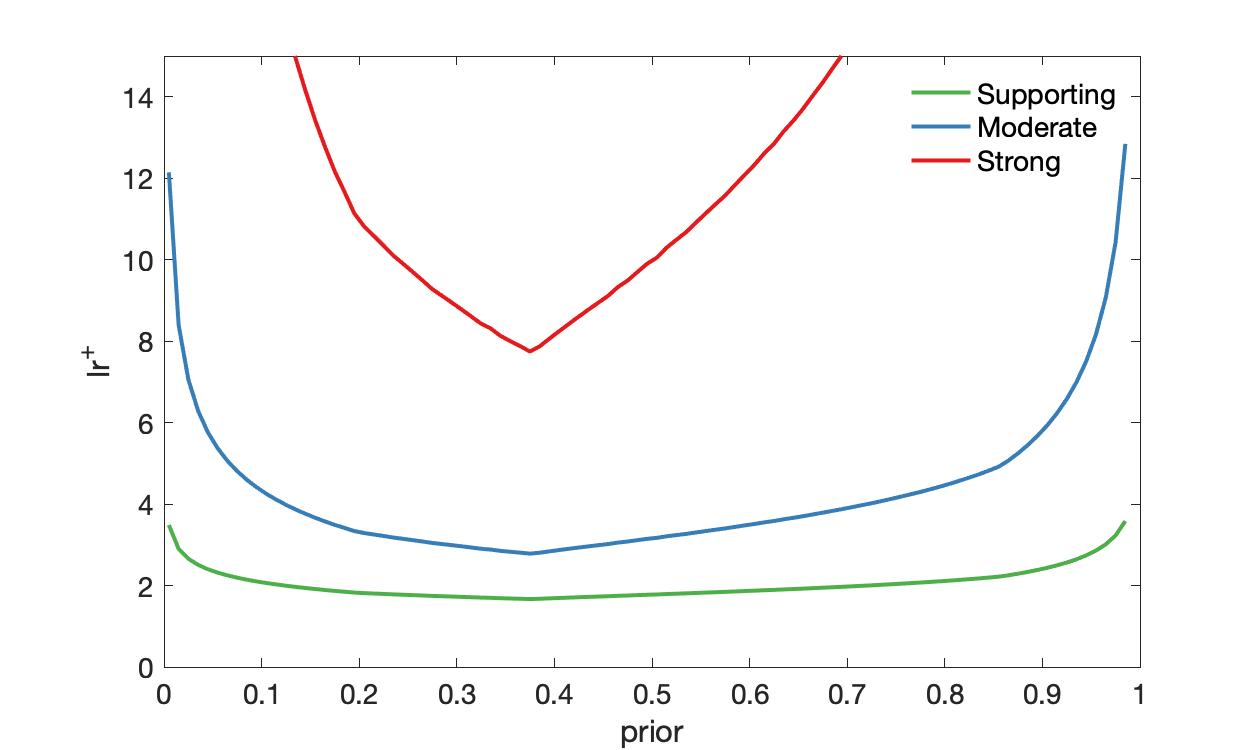
**

**Figure S14:** The local $lr^{+}$ cuttoffs for a variant to qualify as having Supporting, Moderate or Strong evidence for pathogenicity at different priors. The class prior was first selected as each of the values on the axis. The constant *c* was subsequently determined using the approach described in Methods. The values plotted as Supporting, Moderate, and Strong evidence, correspond to the eighth, fourth and square root of *c*, respectively.

# Supplementary Tables

**Table S1**. **CAGI challenges in CAGI1 through CAGI5.** Missense, SNVs and non-coding challenges involve individual genes, and often also include nonsense variants. Rare disease or Mendelian phenotypes indicate the involvement of a single gene, complex traits the involvement of multiple genes. SNVs: single nucleotide variants. Indels: insertions, deletions. WGS: whole genome sequencing.

| **Challenge** | **Edition** | **Genetic scale** | **Phenotypic characterization** | **# variants, traits or genomes** | **# sub-miss-ions** |
| --- | --- | --- | --- | --- | --- |
| Annotate all missense | CAGI5 | Missense | Rare disease | 81,084,849 | 5 |
| Asthma discordant  monozygotic twins | CAGI2 | WGS | Complex trait, multiomics | 8 | 6 |
| Bipolar disorder^29, 132-134^ | CAGI4 | Exomes | Complex trait | 1,000 | 29 |
| BRCA1 & BRCA2^37, 134^ | CAGI3 | Missense, indels, non-coding (splicing) | Cancer | 100 | 14 |
| Breast cancer pharmacogenomics | CAGI2 | Other (multimodal) | Cancer | 54 | 3 |
| CALM1^25, 135, 136^ | CAGI5 | Missense | Rare disease | 1,813 | 7 |
| CBS^37, 134, 137^ | CAGI1 | Missense | Rare disease | 51 | 20 |
| CBS^37, 134, 137, 138^ | CAGI2 | Missense | Rare disease | 84 | 20 |
| CHEK2^37, 41, 134^ | CAGI1 | Missense | Cancer | 41 | 16 |
| CHEK2^113, 135, 136, 139^ | CAGI5 | Missense | Cancer | 53 | 18 |
| Clotting disease^116, 135, 140^ | CAGI5 | Exomes | Complex trait | 103 | 16 |
| Crohn's disease^29, 141^ | CAGI2 | Exomes | Complex trait | 48 | 33 |
| Crohn's disease^29, 141^ | CAGI3 | Exomes | Complex trait | 66 | 61 |
| Crohn's disease^29, 134, 141, 142^ | CAGI4 | Exomes | Complex trait | 111 | 46 |
| ENIGMA BRCA1 and BRCA2^27, 102, 103, 135, 143, 144^ | CAGI5 | Missense | Cancer | 430 | 10 |
| eQTL causal SNPs^59, 145, 146^ | CAGI4 | Regulatory | Complex trait | 9,116 | 33 |
| FCH | CAGI3 | Exomes | Rare disease | 5 | 21 |
| Frataxin^21, 135, 136, 139, 147, 148^ | CAGI5 | Missense | Cancer | 8 | 12 |
| GAA^92, 135, 139^ | CAGI5 | Missense | Rare disease | 357 | 26 |
| HA | CAGI3 | Exomes | Rare disease | 4 | 18 |
| Hopkins clinical panel^61, 149^ | CAGI4 | Gene panel | Rare disease | 106 | 5 |
| ID Panel^28, 150, 151^ | CAGI5 | Gene panel | Rare disease | 146 | 15 |
| MaPSy^57, 152-154^ | CAGI5 | Non-coding (regulatory) | Complex trait & rare disease | 4,964 | 14 |
| Mouse exomes | CAGI2 | Exomes | Rare disease | 4 | 2 |
| MRE11^37^ | CAGI3 | Missense | Cancer | 42 | 23 |
| NAGLU^22, 37, 41, 138, 155^ | CAGI4 | Missense | Rare disease | 163 | 17 |
| NPM-ALK^37^ | CAGI4 | Missense | Cancer | 43 | 4 |
| NBS1^37^ | CAGI3 | Missense | Cancer | 44 | 23 |
| p16^24, 37, 41, 134, 138^ | CAGI3 | Missense | Cancer | 10 | 22 |
| p53 reactivation | CAGI2 | Missense | Cancer | 14,668 | 11 |
| PCM1^135, 139, 156, 157^ | CAGI5 | Missense | Complex trait | 38 | 7 |
| PGP^73^ | CAGI1 | WGS | Complex trait & Mendelian | 10 | 2 |
| PGP^73^ | CAGI2 | WGS | Complex trait & Mendelian | 10 | 4 |
| PGP^73^ | CAGI3 | WGS | Complex trait & Mendelian | 77 | 16 |
| PGP^73^ | CAGI4 | WGS | Complex trait & Mendelian | 23 | 5 |
| PTEN^20, 135, 139^ | CAGI5 | Missense | Cancer | 2,924 | 16 |
| Pyruvate kinase^37, 138, 158-160^ | CAGI4 | Allostery missense | Rare disease | 113 | 5 |
| RAD50 ^37, 41, 134^ | CAGI2 | Missense | Cancer | 69 | 14 |
| Regulation saturation^60, 161, 162^ | CAGI5 | Non-coding (regulatory) | Complex trait | 17,500 | 23 |
| riskSNPs | CAGI2 | SNVs | Complex trait | 58,424 | 7 |
| riskSNPs | CAGI3 | SNVs | Complex trait | 110,477 | 13 |
| SCN5A^37^ | CAGI2 | Missense | Rare disease | 3 | 7 |
| *Shewanella oneidensis*  strain MR-1 | CAGI2, CAGI3 | Other (transposon insertion) | Other | 8 | 0 |
| SickKids^26, 127^ | CAGI4 | WGS | Rare disease | 25 | 4 |
| SickKids^26, 135, 163^ | CAGI5 | WGS | Rare disease | 24 | 9 |
| SUMO ligase^23, 37, 138, 155^ | CAGI4 | Missense | Cancer, rare disease | 682 | 16 |
| TP53 splicing | CAGI3 | Non-coding (splicing) | Cancer | 3 | 5 |
| TPMT^20, 135, 139^ | CAGI5 | Missense | Cancer | 3,736 | 16 |
| Vex-seq^57, 153, 164-166^ | CAGI5 | Non-coding (splicing) | Complex trait & rare disease | 2,059 | 12 |
| Warfarin exomes^29^ | CAGI4 | Exomes | Complex trait | 103 | 9 |

**Table S3 (Additional file 3):**

Summary of results on fully analyzed biochemical effect challenges. The sheet “Full” shows the performance of primary selected method, the baseline model (PolyPhen-2) and Experimental-Max (if available) on seven performance measures for all sheets in Additional file 2: Table S2. The primary selected method is obtained as the first predictor from the ranking procedure described in the next section. The measures include AUC, Truncated AUC, Person’s correlation, Spearman’s correlation, Kendall’s tau, R-squared and Coverage (defined in the next section).  The sheet “Clinical” includes a binarized information on whether the primary selected method, for each sheet (Additional file 2: Table S2) with clinical analysis, reaches supporting, moderate, or strong evidential support in the clinic for three different class priors: data prior, 0.1 and 0.01 (see next section). The sheet “Reduced” shows the summary for 10 challenges (copied from “Full”) used in the paper to report average performance over all challenges. Only one sheet was selected for any challenge with multiple sheets (CBS1, CBS2 and SUMO) in Additional file 2: Table S2. Any sheet without a regression analysis (PCM1, L-PYK1, L-PYK2 and P53) were further excluded from the reduced set. Truncated AUC was not evaluated for two of challenges (Frataxin and P16) in the reduced set, since a clinical analysis was not performed due to small dataset sizes.

**Tables S2, S4, S5, S6 and S7 (Additional files 2, 4, 5, 6, 7, respectively):**

The table below describes the content of Tables S2, S4, S5, S6 and S7.

|  | **Data/challenges** | **Excel sheet names with analysis type** |
| --- | --- | --- |
| Table S2 | Biochemical Effect | \| Regression, Classification, Clinical \| NAGLU, PTEN*, TPMT*, CBS1High, CBS1Low, CBS2High, CBS2Low, SUMO1*, SUMO2*, SUMO3*, CALM1, GAA \| \| --- \| --- \| \| Regression, Classification \| Frataxin*, p16* \| \| Classification,  Clinical \| PCM1, LPYK1, LPYK2 \| \| Classification \| p53* \| |
| Table S4 | Annotate all Missense | \| Classification, Clinical \| AAM1All, AAM2All, AAM1BiClass, AAM2BiClass, AAM1CV, AAM2CV, AAM1HGMD, AAM2HGMD \| \| --- \| --- \| |
| Table S5 | Cancer | \| Classification \| ENIGMA, BRCA \| \| --- \| --- \| |
| Table S6 | Expression and Splicing | \| Regression, Classification \| RegSatEnh1, RegSatEnh2, RegSatProm1, RegSatProm2, VexSeq1, VexSeq2, eQTL2 \| \| --- \| --- \| \| Regression \| MaPSy1, MaPSy2 \| \| Classification \| eQTL1, MaPSy3 \| |
| Table S7 | Complex disease | \| Classification \| Crohns \| \| --- \| --- \| |

* These Biochemical effect sheets involve genes implicated in cancer.

A short description of the sheet without self-explanatory names is given below. For details see Analyzed Challenges.

- AAM1All: Annotate all missense data with pathogenic and benign (P, B) variants from ClinVar and disease mutations (DM) from HGMD.
- AAM2All: Annotate all missense data with pathogenic, likely pathogenic, benign and likely benign (P, LP, B, LB) variants from ClinVar and disease mutations and questionable disease mutations (DM, DM?) from HGMD.
- AAM1BiClass: Annotate all missense data with pathogenic and benign (P, B) variants from ClinVar and disease mutations (DM) from HGMD restricted to the bi-class genes
- AAM2BiClass: Annotate all missense data with pathogenic, likely pathogenic, benign and likely benign (P, LP, B, LB) variants from ClinVar and disease mutations and questionable disease mutations (DM, DM?) from HGMD, restricted to the bi-class genes.
- AAM1CV: Annotate all missense data with pathogenic and benign (P, B) variants from ClinVar.
- AAM2CV: Annotate all missense data with pathogenic, likely pathogenic benign and likely benign (P, LP, B, LB) variants from ClinVar.
- AAM1HGMD: Annotate all missense data with benign (B) variants from ClinVar and disease mutations (DM) from HGMD.
- AAM2HGMD: Annotate all missense data with benign and likely benign (B, LB) variants from ClinVar and disease mutations and questionable disease mutations (DM, DM?) from HGMD.
- CBS1High, CBS1Low: the data from CAGI 1 CBS challenge with relative yeast growth measured in high and low pyridoxine concentration, respectively.
- CBS2High, CBS2Low: the data from CAGI 2 CBS challenge with relative yeast growth measured in high and low pyridoxine concentration, respectively.
- SUMO1, SUMO2, SUMO3: The data from the three subsets of variants created for the SUMO challenge.
- LPYK1, LPYK2: the data from the two subsets of variants created for the L-PYK challenge for predicting the absence of enzymatic activity.
- RegSatEnh1, RegSatEnh2: contains data from the Regulation-Saturation challenge restricted to the enhancers. In RegSatEnh1 the positive label corresponds to increased expression, whereas in RegSatEnh2 it corresponds to decreased expression.
- RegSatProm1, RegSatProm2: contains data from the Regulation-Saturation challenge restricted to the promoters. In RegSatProm1 the positive label corresponds to increased expression, whereas in RegSatProm2 it corresponds to decreased expression.
- MaPSy1, MaPSy2: the data from the MaPSy challenge for separate analysis of allelic ratios measured in vivo and in vitro, respectively.
- MaPSy3: the data from the MaPSy challenge for ESM prediction.
- eQTL1: the data for the first subset of variants from the eQTL challenge corresponding to the prediction of regulatory hit.
- eQTL2: the data for the second subset of variants from the eQTL challenge corresponding to the prediction of emVar hit and log skew allelic ratio.
- VexSeq1, VexSeq2: contains data from the VexSeq challenge. In VexSeq1 the positive label corresponds to over-splicing, whereas in VexSeq2 it corresponds to under-splicing$.$

The table below gives the measures corresponding to the three analysis types that are reported in the tables. Only the measures corresponding to the applicable analysis type is reported in a given sheet. A subset of the Reported measures are used as Selection measures to determine the order in which methods are displayed in the tables as well as for picking the methods displayed in the figures. Coverage (COV), the proportion of variants for which a method makes a prediction, is additionally reported in all sheets.

| **Analysis type** | **Reported measures** | **Selection measures** |
| --- | --- | --- |
| Regression | R-squared ($R^{2})$, RMSE, Pearson’s correlation ($r$), Spearman’s rank correlation, Kendall’s Tau ($\tau$) | Pearson’s correlation, Kendall’s Tau ($\tau$) |
| Classification | AUC | AUC |
| Clinical | Truncated AUC, log-log AUC, TPR, FPR, $LR^{+}$, $LR^{-}$, DOR, MCC, PPV, PPP | Truncated AUC |

Each clinical measure, except Truncated AUC and Log-log AUC, is computed w.r.t. a threshold for the method score, corresponding to one of the three evidence levels: supporting, moderate and strong. The value of an evidence threshold depends on the prior (proportion of pathogenic variants) as discussed in the Methods section. We consider the following three priors for all biochemical effect sheets with clinical analysis.

1. **Data prior**: The proportion of pathogenic variants present in the dataset. The value of the data prior is determined from the challenge specific class boundary used to create binary classes (ground truth for classification) from the experimental values. For details on how the class boundaries were determined for each challenge refer to Analyzed Challenges.
2. **0.1**: An approximation of the proportion of pathogenic variants encountered in a clinic in a diagnostic setting; see Methods.
3. **0.01**: An approximation of the proportion of pathogenic variants encountered in a clinic in a screening setting; see Methods.

For each of three class priors, three evidence thresholds are determined, giving a total of nine thresholds. Clinical measures are computed for all nine thresholds. In case of Annotate all missense sheets, only 0.1 and 0.01 are considered as the class priors. This gives a total of six thresholds for which the clinical measures are reported. The class priors used to compute the evidence thresholds, are also listed in the sheets containing the clinical measures. $LR^{+}$and $LR^{-}$ reported in the sheets are global. Clinically relevant local $lr^{+}$values are not reported in the sheets, since the local $lr^{+}$ values at the clinical thresholds are completely determined by the assumed prior as given in Figure S14.

**Coverage (Cov):**

We define coverage of a method as the percent of variants for which the method makes a prediction. If both regression and classification analyses are applicable to a sheet and the size of the classification data is different from that of the regression data, then the coverage is computed relative to the size of the classification set; i.e., the proportion of variants in the classification set for which the method makes a prediction. The variants for which an experimental value could not be recorded are excluded from the regression and classification set and consequently do not affect the coverage calculation.

**Methods reported:**

A sheet contains evaluation of one representative method from each group that made a submission for the corresponding CAGI challenge. Additionally, performance of baseline methods and Experimental-Max (if applicable) were also reported. Each method takes two columns in the sheet: one containing the measures evaluated for the method on the entire data set and the other containing confidence intervals (CI) of those measures from 1000 bootstrap samples. In case of Experimental-Max, the first column contains the mean of each measure computed with 1000 Experimental-Max predictions, which are also used to compute the confidence interval. The coverage (COV) of the method is reported without a confidence interval.

The order in which the methods are reported in the sheet is determined as follows. All available methods, including multiple submissions from the same group, baseline methods and Experimental-Max, are first ranked separately on the four selection measures: Pearson’s correlation, Kendall’s $\tau$, AUC and Truncated AUC. In this manner, each method gets four rank values. An average of the four ranks is taken to determine the method's final rank. Additionally, each method is assigned a nominal value by taking an average over the four measures. The nominal values are used to resolve ties in the average ranks. Any ties still present are resolved randomly. If a group has multiple methods, the representative method is picked as the method with the highest rank in that group. The representative methods, except any baselines and Experimental-Max, are listed first in the order of their ranks left to right, followed by the baselines and then the Experimental-Max.

If any method has a coverage below 0.9, it is initially excluded from the main ranking. All such methods are ranked separately in the same manner as described above in a secondary ranking. Then the main ranking is extended to include these methods at its end. This process ensures that any low coverage method, in spite of better performance on the four measures, appears after all the high coverage methods. Any baseline methods and the Experimental-Max still appear at the end.

If regression is not applicable to a sheet, the two correlation coefficients are excluded from the ranking. Similarly, if classification is not applicable, AUC and Truncated AUC are excluded. If classification is applicable, but the clinical analysis is not, then Truncated AUC is excluded.

PolyPhen-2 is used as a baseline for all biochemical effect challenges, except P53, the two cancer challenges and Annotate all missense. SIFT was used as an additional baseline for Annotate all missense. No reasonable baselines were available for expression, splicing and complex disease challenges. In addition to the methods submitted for the Annotate all missense, all available predictors from dbNSFP v3.5 were also analyzed.

**Dataset properties reported**

In each sheet there is a small table, below the performance evaluation table, giving the size and the “data prior” (proportion of positives in the data). If the sheet also had a regression type analysis, the size of the data used for regression evaluation might be different from the size of the data for classification evaluation. If the sizes are indeed different, then regression data size is reported separately from the classification data size.
