# Supplementary material for: CAGI, the Critical Assessment of Genome Interpretation, establishes progress and prospects for computational genetic variant interpretation methods
Source: Genome Biol. 2024 Feb 22;25:53. doi: 10.1186/s13059-023-03113-6 (PMC10882881; doi:10.1186/s13059-023-03113-6)
Supplement: Supplementary file 8 — Additional file 8. Review history. [file 13059_2023_3113_MOESM8_ESM.docx]

Review History

**First round of review**

**Reviewer 1**

**Are you able to assess all statistics in the manuscript, including the appropriateness of statistical tests used?** Yes, and I have assessed the statistics in my report.

**Comments to author:**

This paper presents the accumulated insights from the CAGI community experiments (ten years), where the quality of genetic variant interpretation is assessed based on people blindly submitting predictions in various categories. Subsequently these are being compared by independent assessors to known results from work being done elsewhere. Altogether there has been 738 submissions to the five rounds of challenges, clearly a theme of very high importance for today's clinical genomics agenda and similarly for basic research.

Overall, I find the paper impressive. It has been a major joint effort to boil down all the experiments to key observations and a brief walkthrough in relation to perspectives and future developments.

I have a few suggestions for improvement. The Introduction describes the area in terms of different routes to interpretation. I think the text in this part of the paper could introduce the area and efforts slightly better. As it reads it is a bit biased towards methods that directly aim for predicting e.g., pathogenicity. If we look at the use of bioinformatics tools in this area, tools which are not initially made to assess pathogenicity directly are presumably by far the most used for this purpose. Tools aiming at predicting functional features, say an intron splice site, or a signal peptide cleavage site, used manually, are frequently used. The text could mention them better and split the "computational methods" in two (as it also is done later in the paper).

Figure 1 provides a graphical overview of the 50 challenges and their subtypes, and it is a good way to kick this paper off. However, the figure can be improved, in B) there is no text on the y-axes, and in C) it is unfortunate that the colors are reused in the two pie charts. Another improvement could be to color the challenges in D) using the colors from one of the pie charts. Then the reader would also be informed of the development over time in the types of challenges.

The paper is (likely unintentionally) quite reductionist in its presentation of "pathogenicity" as a local genome phenomenon. In the Introduction it is in my view not stressed sufficiently that a genome often contains compensatory variation that lowers or eliminates the risk that a variant otherwise confers on an individual. This contributes significantly to the difficultly of the problem, also clinically. More and more reports on compensation are being published, like the work describing human essential gene knockouts being well and reasonably alive (https://www.science.org/doi/10.1126/science.aac8624). In the Discussion it is mentioned that CAGI also plans to increase evaluation of the combinatorial effect of variants in clinical applications with whole-genome studies for both rare and complex phenotypes, and it would be good to have the non-local aspects of the problem mentioned in the Introduction as well.

In the Figure 2 caption, it is indicated that the showcased challenges are "representative"? I find this confusing and suggest that the word is removed. In the CAGI domain there is a general lack of representative data for benchmarking as the text later in the paper also correctly states. The challenges are based on what is available and the assessors are normally good at pointing at problems in the underlying data foundation for predicting outliers etc. When discussing subjective choices in metrics the paper is excellent in adding disclaimers.

In conclusion, I find the paper of high value and technically sound. It was a pleasure to read it.

**Reviewer 2**

**Are you able to assess all statistics in the manuscript, including the appropriateness of statistical tests used?** No, I do not feel adequately qualified to assess the statistics.

**Comments to author:**

Please consider the following questions:

- Are the methods appropriate to the aims of the study, are they well described, and are necessary controls included? If not, please specify what is required.

Yes

- Are the conclusions adequately supported by the data shown? If not, please explain

Yes

- Are sufficient details provided to allow replication and comparison with related analyses that may have been performed? If not, please specify what is required.

Yes

- Does the work represent a significant advance over previously published studies?

Yes

- Is the paper of broad interest to others in the field, or of outstanding interest to a broad audience of biologists?

Yes

advancing computational prediction of genetic variant impact, especially in the context of disease. Their involvement in the five editions of the CAGI community experiment, where they made blind predictions and underwent rigorous evaluation by independent assessors, exemplifies their commitment to excellence.

Their findings reveal the immense potential of current methods, despite their imperfections, in revolutionizing both research and clinical applications. Notably, their expertise in missense variant interpretation has led to increasingly accurate estimations of biochemical effects, especially for clinical pathogenic variants, including challenging cases and cancer-related variants.

Furthermore, their exploration of methods for regulatory variants and complex trait disease risk has opened doors to auxiliary clinical applications. Their cutting-edge approach, coupled with the integration of large and robust datasets, promises even more groundbreaking progress in the field.

In summary, your colleague's work not only reflects their exceptional skills and dedication but also offers profound contributions to the advancement of precision medicine and genetic research. Their achievements are a testament to their invaluable expertise and potential to shape the future of computational genomics.

**Authors Response**

**Point-by-point responses to the reviewers’ comments:**

Thank you for the reviews of our paper. We are pleased to see positive news. As requested, we have addressed the comments of reviewer 1 and have made the appropriate changes to fit the journal’s requirements, as detailed below. We believe the paper is now in a suitable form for publication.

**Responses to reviewers:**

*Reviewer comment: This paper presents the accumulated insights from the CAGI community experiments (ten years), where the quality of genetic variant interpretation is assessed based on people blindly submitting predictions in various categories. Subsequently these are being compared by independent assessors to known results from work being done elsewhere. Altogether there has been 738 submissions to the five rounds of challenges, clearly a theme of very high importance for today's clinical genomics agenda and similarly for basic research. Overall, I find the paper impressive. It has been a major joint effort to boil down all the experiments to key observations and a brief walkthrough in relation to perspectives and future developments.*

Response: We thank the reviewer for these comments. It was indeed a major effort!

*Reviewer comment: I have a few suggestions for improvement.*

Response: We have addressed each of these as detailed below.

*Reviewer comment: The Introduction describes the area in terms of different routes to interpretation. I think the text in this part of the paper could introduce the area and efforts slightly better. As it reads it is a bit biased towards methods that directly aim for predicting e.g., pathogenicity. If we look at the use of bioinformatics tools in this area, tools which are not initially made to assess pathogenicity directly are presumably by far the most used for this purpose. Tools aiming at predicting functional features, say an intron splice site, or a signal peptide cleavage site, used manually, are frequently used. The text could mention them better and split the "computational methods" in two (as it also is done later in the paper).*

Response: We have added the following text after the ‘Computational methods…’ sentence, emphasizing that there are two types of methods. ”Some computational approaches aim to directly relate sequence variation to disease or other phenotypes; for example, using evolutionary conservation.11 Other methods suggest impact on disease only secondarily as they aim to relate genetic variants to functional properties such as effects on protein stability,12 intermolecular interactions,13 splicing,14 expression,15 or chromatin organization.16

*Reviewer comment: Figure 1 provides a graphical overview of the 50 challenges and their subtypes, and it is a good way to kick this paper off. However, the figure can be improved, in B) there is no text on the y-axes, and in C) it is unfortunate that the colors are reused in the two pie charts. Another improvement could be to color the challenges in D) using the colors from one of the pie charts. Then the reader would also be informed of the development over time in the types of challenges.*

Response: A new version of Figure 1 is included. Axes have been added in panel B) and non-overlapping colors used in panel C). Genetic scale and phenotypic characterization is provided for each challenge in D) according to the coloring scheme given in C).

*Reviewer comment: The paper is (likely unintentionally) quite reductionist in its presentation of "pathogenicity" as a local genome phenomenon. In the Introduction it is in my view not stressed sufficiently that a genome often contains compensatory variation that lowers or eliminates the risk that a variant otherwise confers on an individual. This contributes significantly to the difficultly of the problem, also clinically. More and more reports on compensation are being published, like the work describing human essential gene knockouts being well and reasonably alive (https://www.science.org/doi/10.1126/science.aac8624). In the Discussion it is mentioned that CAGI also plans to increase evaluation of the combinatorial effect of variants in clinical applications with whole-genome studies for both rare and complex phenotypes, and it would be good to have the non-local aspects of the problem mentioned in the Introduction as well.*

Response: We believe that reviewer is referring to non-local effects in rare ‘monogenic’ disease, since of course complex trait disease is inherently non-local and methods explicitly incorporate many variants. For cancer it, is also recognized that the disease phenotype is typically driven by effects from multiple variants. As the reviewer notes, we do acknowledge the issue for rare disease in the Discussion. The reviewer requests it also be address in the Introduction. However, the Introduction is intended to be fairly streamlined and does not go in to this level of detail. Thus, we have instead added to text to ‘Diversity of methods’ section, after ‘The relationship between fitness, pathogenicity and function is complex, perhaps limiting performance. To partly address this, some methods also exploit functional roles of specific sequence positions, particularly by utilizing UniProtKB annotations and predicted structural and functional properties as follows:

”Current methods typically address the effect of single variants in isolation from possible epistatic factors although many apparently monogenic diseases are influenced by modifier variants. For example, severity of cystic fibrosis is affected by several genes beyond CFTR,4 and studies of loss of function variants in general populations revealed cases where a strong disease phenotype is expected but not observed, implying the presence of compensating variants.42”

*Reviewer comment: In the Figure 2 caption, it is indicated that the showcased challenges are "representative"? I find this confusing and suggest that the word is removed. In the CAGI domain there is a general lack of representative data for benchmarking as the text later in the paper also correctly states. The challenges are based on what is available and the assessors are normally good at pointing at problems in the underlying data foundation for predicting outliers etc. When discussing subjective choices in metrics the paper is excellent in adding disclaimers.*

Response: Thank you. ‘representative’ has been replaced by ‘example’

*Reviewer comment: In conclusion, I find the paper of high value and technically sound. It was a pleasure to ready it.*

Response: We thank the reviewer for these supportive words and for the useful feedback.
